# Phosphonic acid tagged carbon quantum dots encapsulated in SBA-15 as a novel catalyst for the preparation of *N*-heterocycles with pyrazolo, barbituric acid and indole moieties

**DOI:** 10.1038/s41598-022-24553-3

**Published:** 2022-12-02

**Authors:** Milad Mohammadi Rasooll, Hassan Sepehrmansourie, Mahmoud Zarei, Mohammad Ali Zolfigol, Sadegh Rostamnia

**Affiliations:** 1 Department of Organic Chemistry, Faculty of Chemistry, Bu-Ali Sina University, Hamedan, 6517838683 Iran; 2 Department of Chemistry, Faculty of Science, University of Qom, Qom, 3716146611 Iran; 3 Organic and Nano Group (ONG), Department of Chemistry Iran University of Science and Technology (IUST), PO Box 16846-13114, Tehran, Iran

**Keywords:** Heterogeneous catalysis, Environmental chemistry

## Abstract

Herein, we have presented a new insight for the synthesis of a hybrid heterogeneous catalyst. For this purpose, phosphonic acid tagged carbon quantum dots of CQDs-N(CH_2_PO_3_H_2_)_2_ encapsulated and assembled in channels of SBA-15 using a post-modification strategy. The mesoporous catalyst of functionalized carbon quantum dots (CQDs) was characterized by several techniques. CQDs-N(CH_2_PO_3_H_2_)_2_/SBA-15 as an excellent catalyst was applied for the preparation of novel pyrazolo[4′,3′:5,6]pyrido[2,3-*d*]pyrimidine derivatives by using pyrazole, barbituric acid and indole moieties at 100 °C under the solvent-free condition. The present work shows that a significant increase in the catalytic activity can be achieved by a rational design of mesoporous SBA-15 modified with CQDs for the synthesis of biological active candidates. The synthesized compounds did not convert to their corresponding pyridines via an anomeric-based oxidation mechanism.

## Introduction

In recent years, composite materials have become well-known as a catalyst, absorbent and sensor in modern sciences^1–3^. The research and development in designing a composite with high selectivity, performance and sensitivity is an urgent need to expand new frontiers of knowledge^4,5^. Quantum dots based on carbon, nitrogen and graphene as nano-materials with small size 10 nm have exhibited great potential uses in photocatalysis, biosensing, heavy metal elements sensing and biomolecule/drug delivery^6–9^. Despite the success of existing materials and technologies, the stability of CDs especially in harsh chemical and physical conditions and weak thermal stability is still highly desirable. Also, mesoporous silica catalysts are much more functional due to their easy and fast separation from reaction media, which is high surface area, pore volume and tunable pore size^10–12^. Besides, a composite material is composed of quantum dots and mesoporous silica as a new property which is investigated as a catalyst, sensor, extraction and determination^13–16^. However, carbon quantum dots (CQDs) encapsulated in the channel of SBA-15 would produce improved or new properties. Therefore, the application of composite materials in the field of catalyst knowledge has a great interest due to their ability to catalyze a wide range of organic synthesis compounds. In this field, we have applied carbon quantum dots (CQDs) and mesoporous silica as a catalyst for the preparation of pyridines, 1,4-dihydropyridines, 4*H*-pyrans and spiropyran compounds^17–19^.

**Figure 1:**
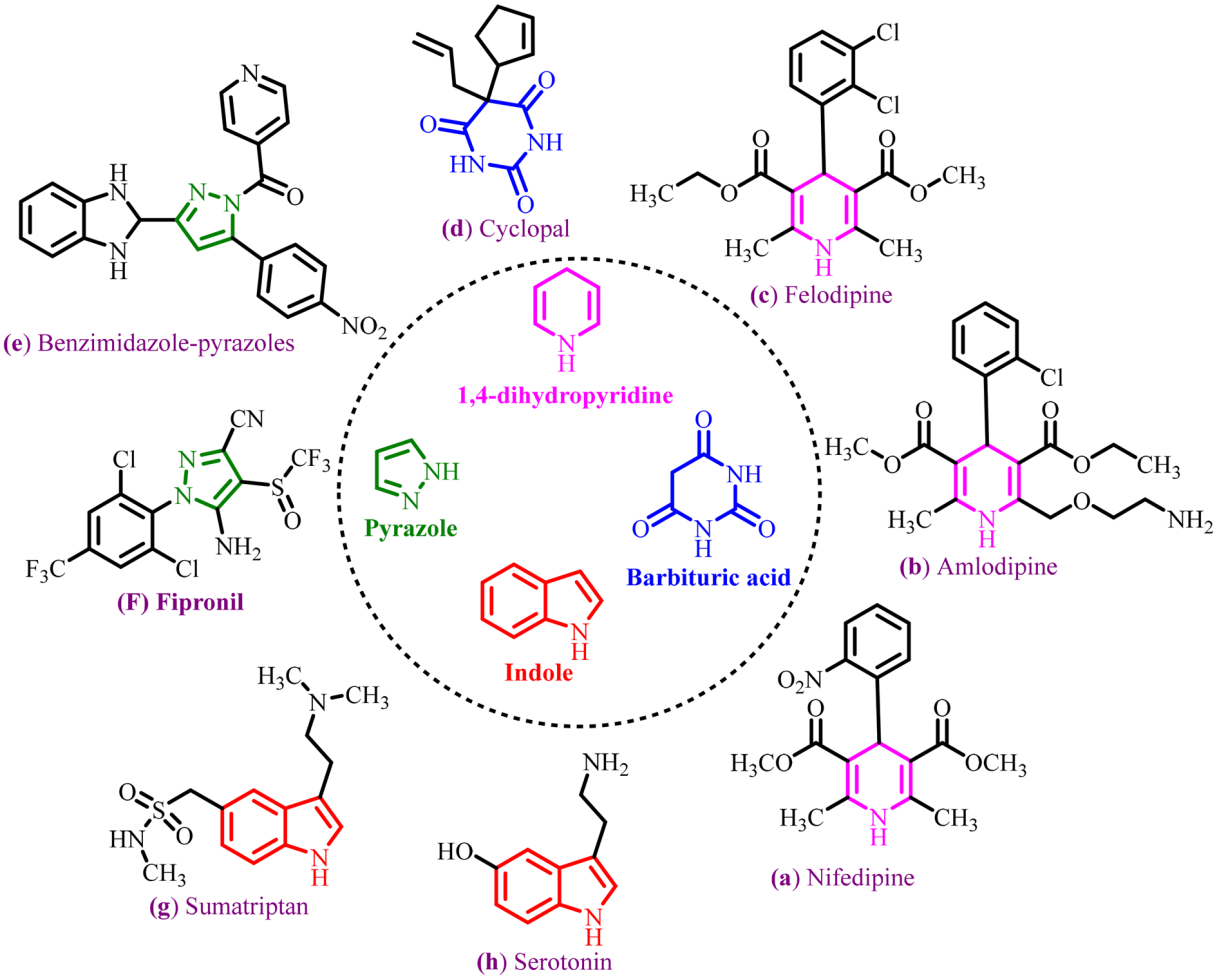
Diversity of pyrrole, barbituric acid and indole based on 1,4-dihydropyridines (1,4-DHPs) structure with biological properties.

Multicomponent reactions (MCRs) as a versatile method have been also of great interest for the preparation of a wide range of *N*-heterocycle compounds with biological activity^20–29^. Therefore, *N*-heterocycles such as 1,4-dihydropyridines (1,4-DHPs) scaffolds make a variety in the drug structures such as Nifedipine (a), Amlodipine (b) and Felodipine (c) (Fig. 1)^30–32^. Also, the biological significance of 1,4-DHPs was investigated in antitumor, anti-ischemic, analgesic, anti-Alzheimer, anti-hypertensive, insecticidal, cardiovascular, anti-inflammatory and anti-microbial conditions^33–35^. Furthermore, diversity of pyrrole, barbituric acid and indole-based scaffolds such as Cyclopal (d), Benzimidazole-pyrazoles (e), Fipronil (f) Sumatriptan (g) and Serotonin (h) are important in the areas of medicine and agriculture^36–39^. However, some of these methods are accompanied by restrictions including the use of expensive starting materials, harsh reaction conditions, long reaction times and low yields. Therefore, the development of new, efficient and versatile catalysts is still on demand.

**Figure 2:**
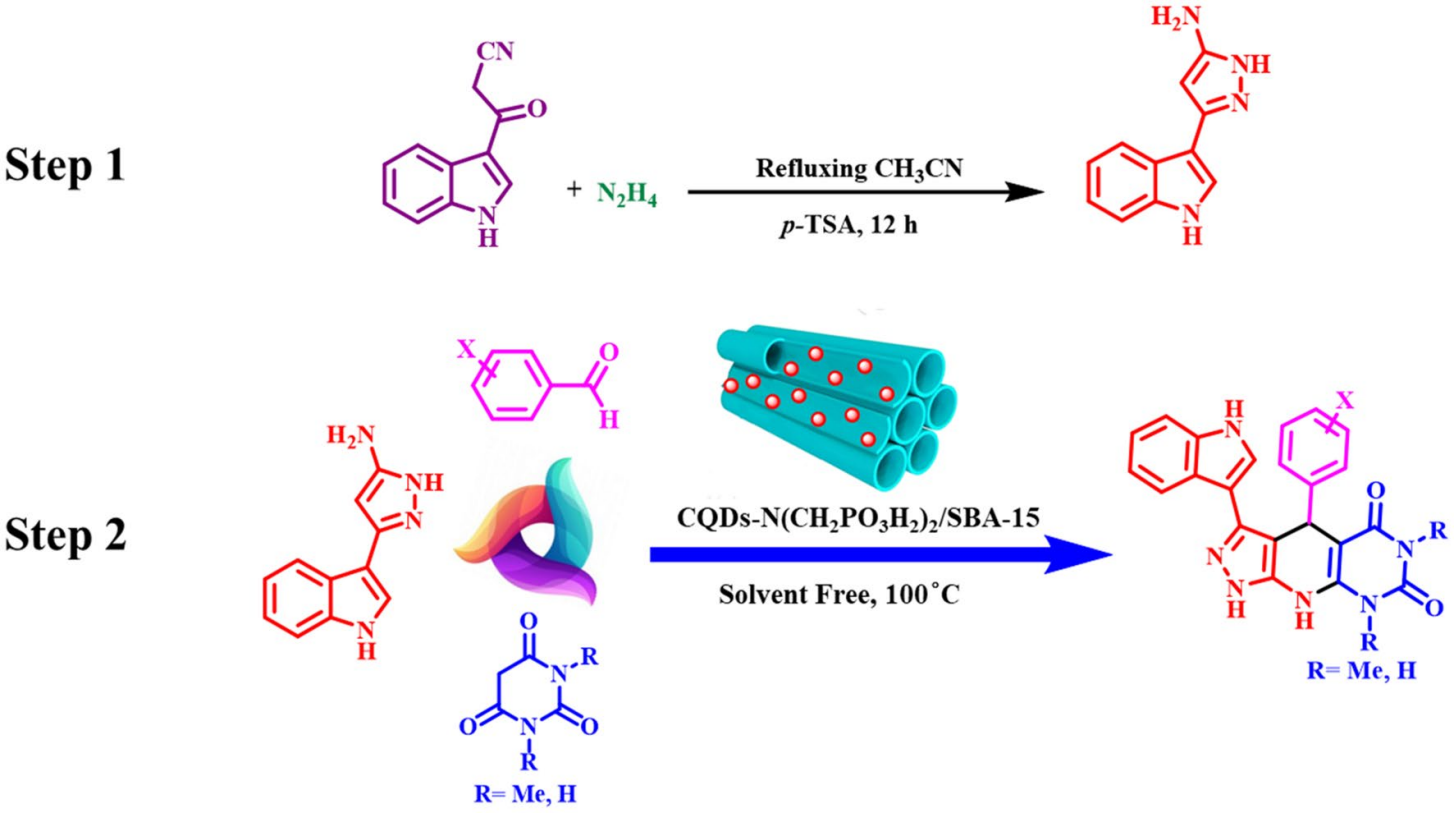
Synthesis of pyrazolo[4′,3′:5,6]pyrido[2,3-*d*]pyrimidine derivatives using CQDs-N(CH_2_PO_3_H_2_)_2_/SBA-15.

In this strategy, we introduced a convenient method to immobilize phosphonic acid tagged carbon quantum dots (CQDs) in the channels of SBA-15. Then, it was used in the synthesis of *N*-heterocycle compounds with pyrazole, barbituric acid and indole moieties. The novel pyrazolo[4′,3′:5,6]pyrido[2,3-*d*]pyrimidine derivatives were synthesized in the presence of CQDs-N(CH_2_PO_3_H_2_)_2_/SBA-15 as an excellent mesoporous heterogeneous catalyst (Fig. 2).

## Experimental

### Synthesis of CQDs-N(CH_2_PO_3_H_2_)_2_ and SBA-15

Phosphonic acid tagged carbon quantum dots of CQDs-N(CH_2_PO_3_H_2_)_2_ and SBA-15 were synthesized according to our previously reported works^19,40–44^.

### Synthesis of CQDs-N(CH_2_PO_3_H_2_)_2_/SBA-15 composite

For this purpose, in a 100 mL round-bottomed flask, a mixture of CQDs-N(CH_2_PO_3_H_2_)_2_ (1 g) and SBA-15 (1 g) and toluene (50 mL) was stirred under reflux conditions for 12 h. After this time, the reaction mixture slowly cooled down to room temperature and participate was separated by a centrifuge (2 × 4000 rpm). Then, the residual small amount of toluene was evaporated and the brown participate was triturated with EtOH (2 × 5 mL) and dried under a powerful vacuum at 70 °C. We have synthesized CQDs-N(CH_2_PO_3_H_2_)_2_/SBA-15 composite under the ambient and air atmosphere. Therefore, the environment of the reaction does not affect the synthesis of catalyst.

### General procedure for the preparation of *N*-heterocycle compounds with pyrazole barbituric acid and indole tags using CQDs-N(CH_2_PO_3_H_2_)_2_/SBA-15

In the first step, the 5-(1*H*-Indol-3-yl)-2*H*-pyrazol-3-ylamine was synthesized according to the procedure reported in the literature (Fig. 2)^45^. In the second step, in a 10 mL round-bottomed flask, a mixture of aldehydes (1 mmol), pyrimidine-2,4,6(1*H*,3*H*,5*H*)-trione derivatives (1 mmol) and as-synthesized 5-(1*H*-indol-3-yl)-1*H*-pyrazol-3-amine (1 mmol) were mixed in the presence of 10 mg of CQDs-N(CH_2_PO_3_H_2_)_2_/SBA-15 as a novel heterogeneous catalyst. Then, the mixture temperature was slowly raised up to 100 °C and it was stirred at 100 °C under the solvent-free condition for appropriate time (Tables 2 and 3). After the completion of the reactions which were monitored by the TLC technique, 10 mL of hot EtOH was added to the reaction mixture and the solid catalyst was separated by centrifugation (4000 rpm for 10 min). Finally, after the evaporation of the solvent at room temperature, the pure product was obtained and washed with cool ethanol for several times.

## Results and discussion

**Figure 3:**
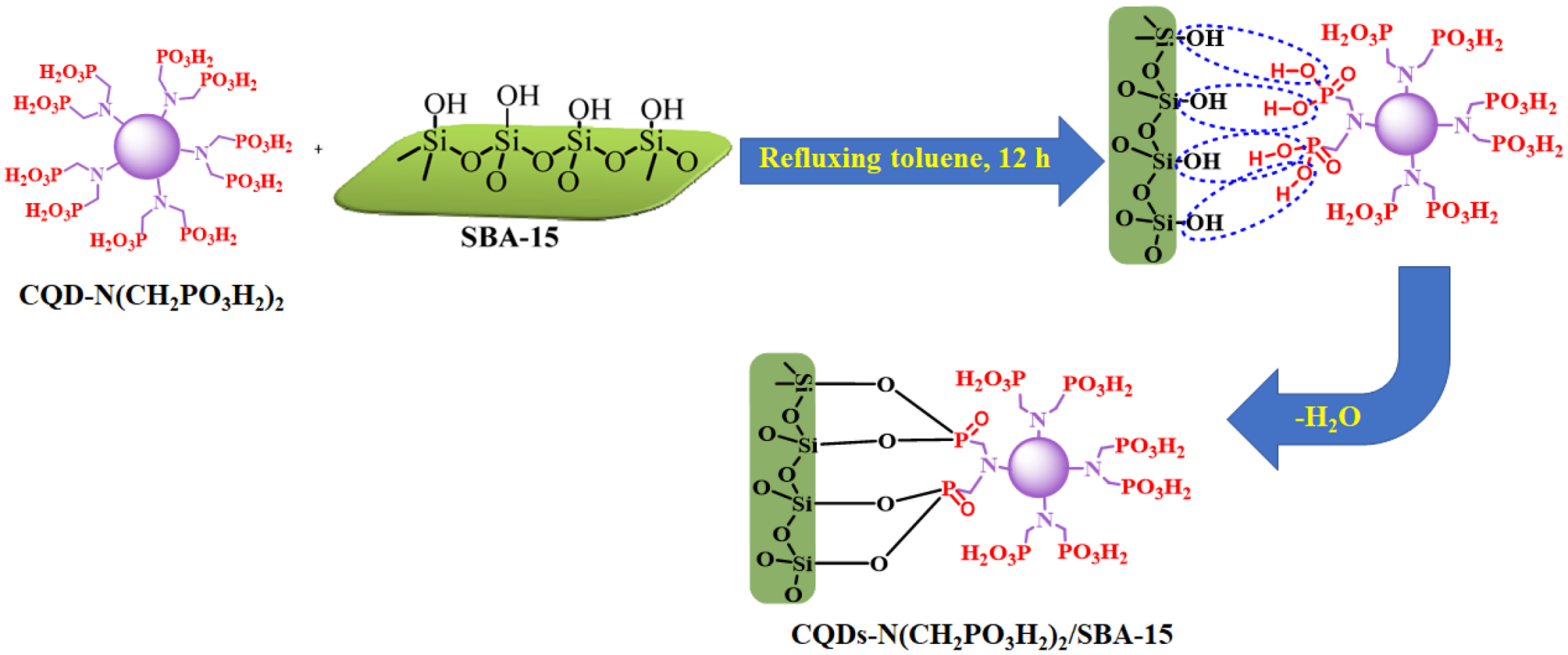
Schematic preparation of CQDs-N(CH_2_PO_3_H_2_)_2_/SBA-15 as a desired catalyst.

In continuation of our investigation on the mesoporous SBA-15 and CQDs as catalysts in organic synthesis, herein we wish to develop the knowledge of catalysts, consisting of mesoporous SBA-15 and CQDs as highly active composite catalysts^19,46–48^. With this aim, CQDs-N(CH_2_PO_3_H_2_)_2_/SBA-15 as a novel heterogeneous catalyst was synthesized, characterized and applied in the synthesis of target organic molecules (Fig. 3). To determine the structural nature and morphology of the CQDs-N(CH_2_PO_3_H_2_)_2_/SBA-15 various techniques such as XRD, SEM, TEM, *N*_*2*_ adsorption–desorption isotherms, FT-IR, energy dispersive X-ray (EDS) and SEM-elemental mapping were applied.

**Figure 4:**
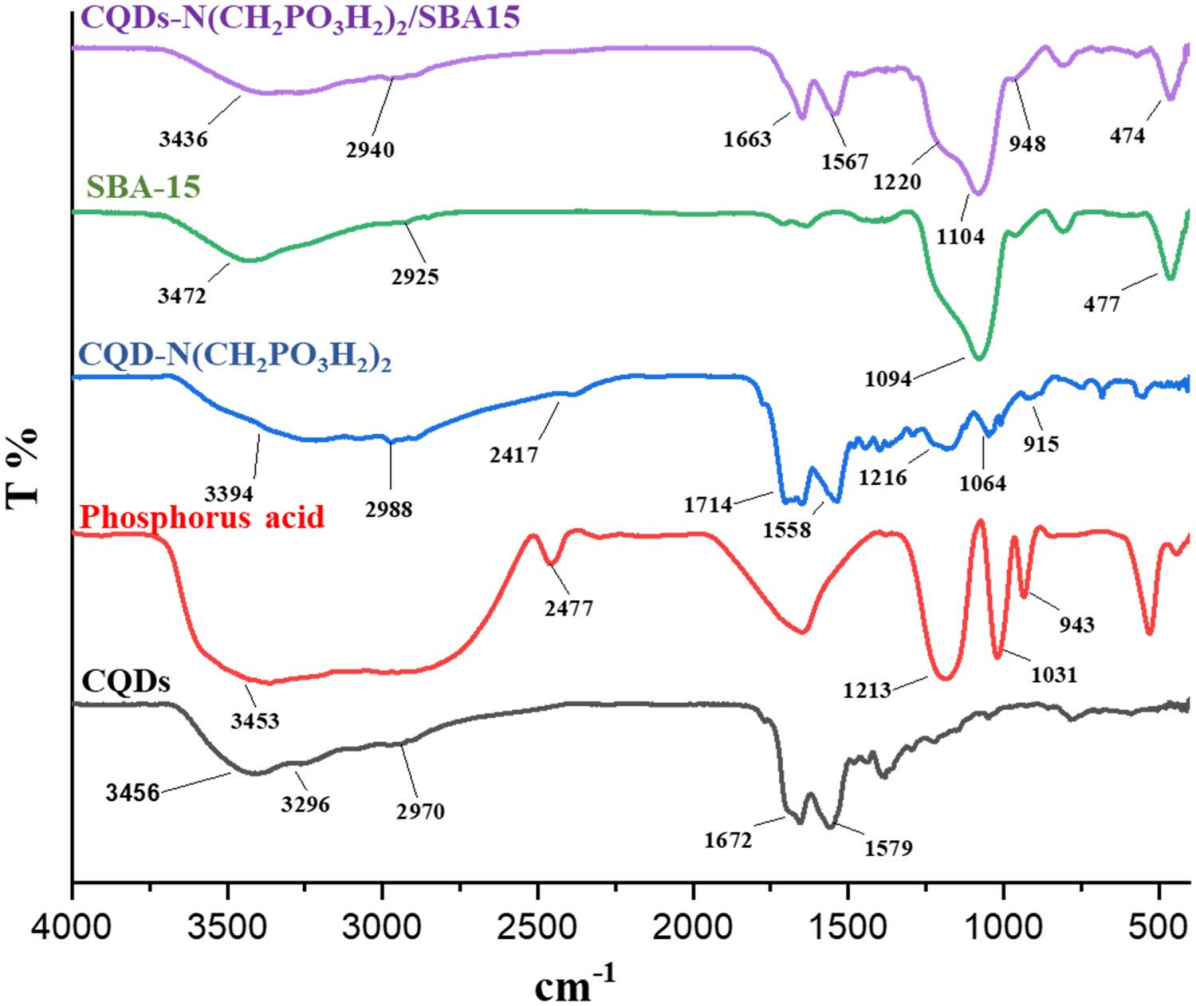
FT-IR spectra of CQDs, phosphorus acid, CQDs-N(CH_2_PO_3_H_2_)_2_, SBA-15 and CQDs-N(CH_2_PO_3_H_2_)_2_/SBA-15.

The FT-IR spectrum of CQDs, phosphorus acid, CQD-N(CH_2_PO_3_H_2_)_2_, SBA-15 and CQDs-N(CH_2_PO_3_H_2_)_2_/SBA-15 as a desired catalyst were compared in Fig. 4. The broad peak at 2600–3400 cm^−1^ indicates the OH of PO_3_H_2_ functional group. The aromatic C-H and C = C stretches bands are, respectively, at 2940 and 1663 cm^−1^. The absorption bands at 948 and 1058 cm^−1^ are related to P–O bond stretching and the band at 1104 cm^−1^ is related to P=O. The peak in the 1714 cm^−1^ indicates the CO of the carbonyl group in the CQDs-N(CH_2_PO_3_H_2_). Also, the absorption band at 1094 cm^−1^ was linked to the stretching vibrational of SiO_2_ group in the SBA-15. The changes in different stages of synthesis indicated the preparation of the catalyst.

**Figure 5:**
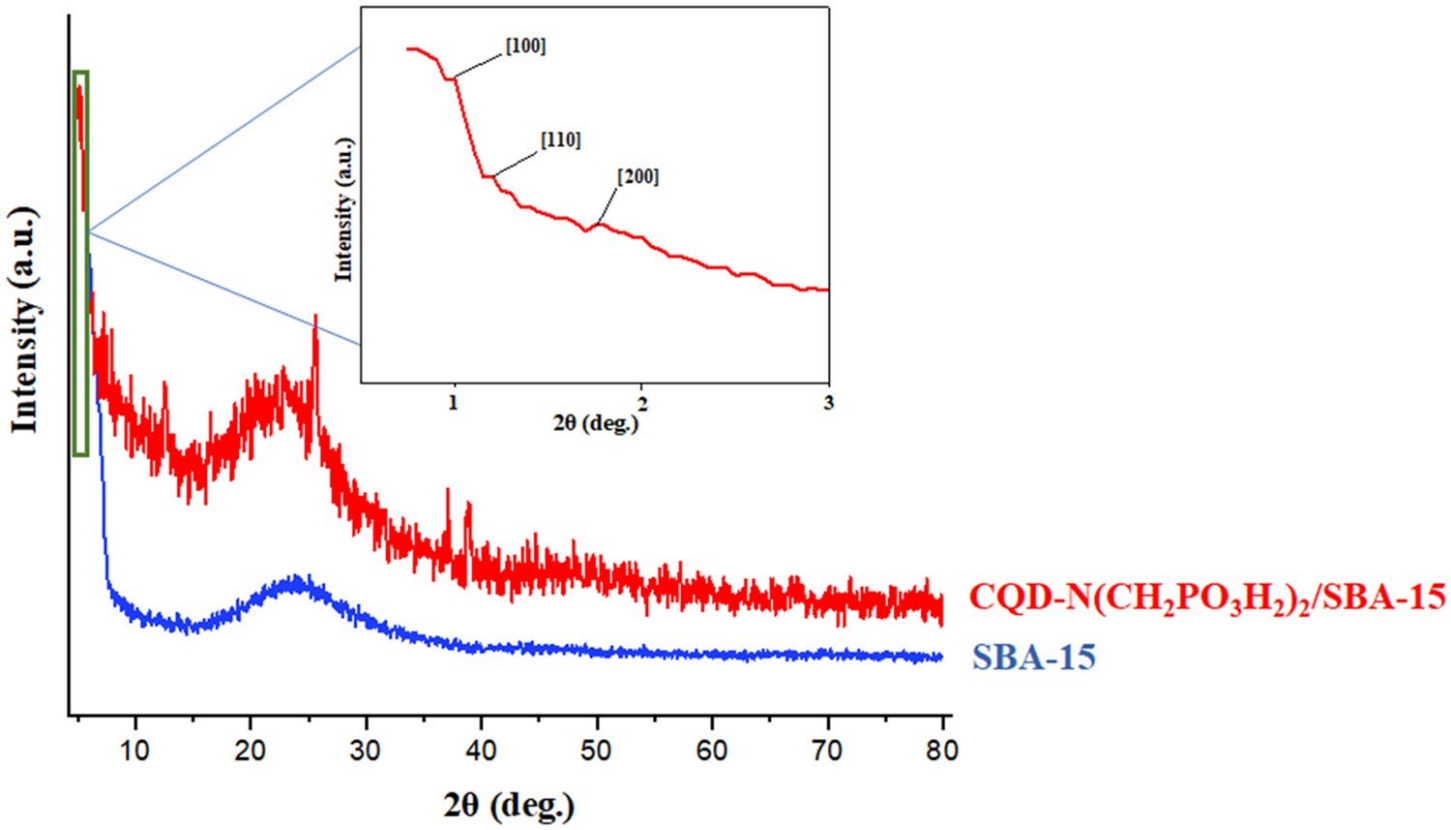
Comparison Low angle and PXRD of CQDs-N(CH_2_PO_3_H_2_)_2_/SBA-15 with SBA-15.

**Figure 6:**
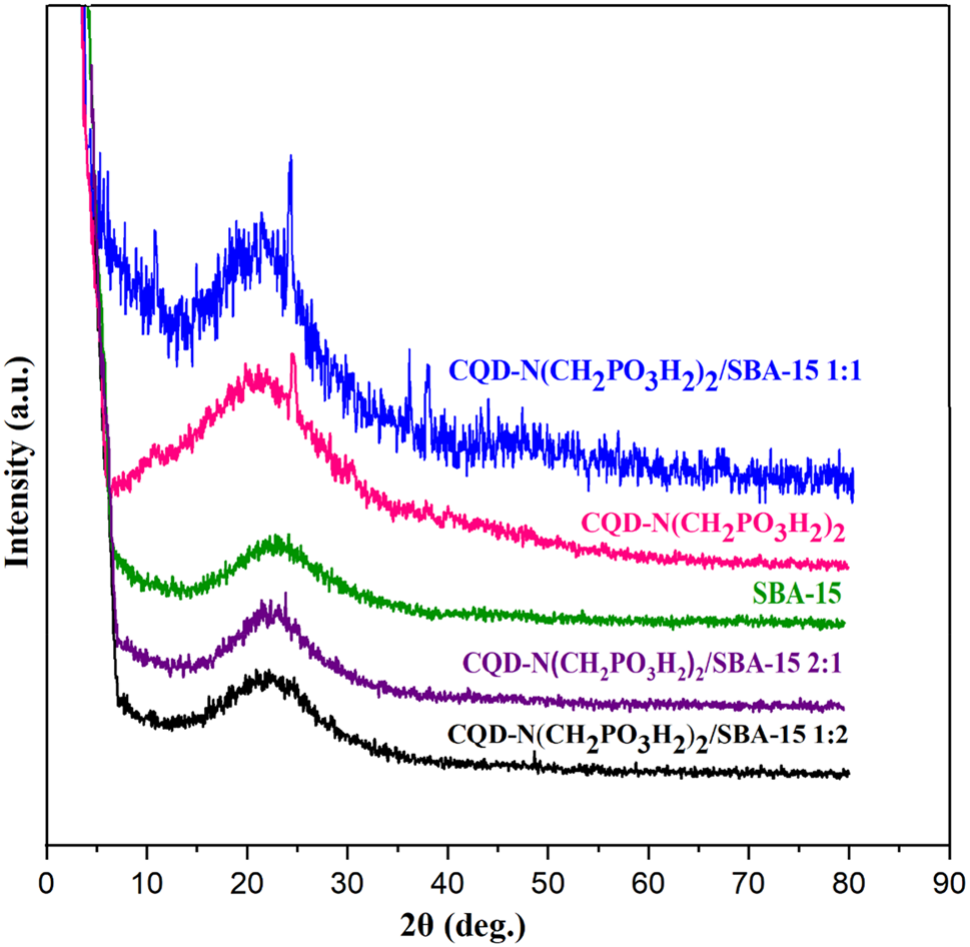
Comparison PXRD of CQDs-N(CH_2_PO_3_H_2_)_2_/SBA-15 (1:1), CQD-N(CH_2_PO_3_H_2_)_2_, SBA-15, CQDs-N(CH_2_PO_3_H_2_)_2_/SBA-15 (2:1) and CQDs-N(CH_2_PO_3_H_2_)_2_/SBA-15 (1:2).

**Figure 7:**
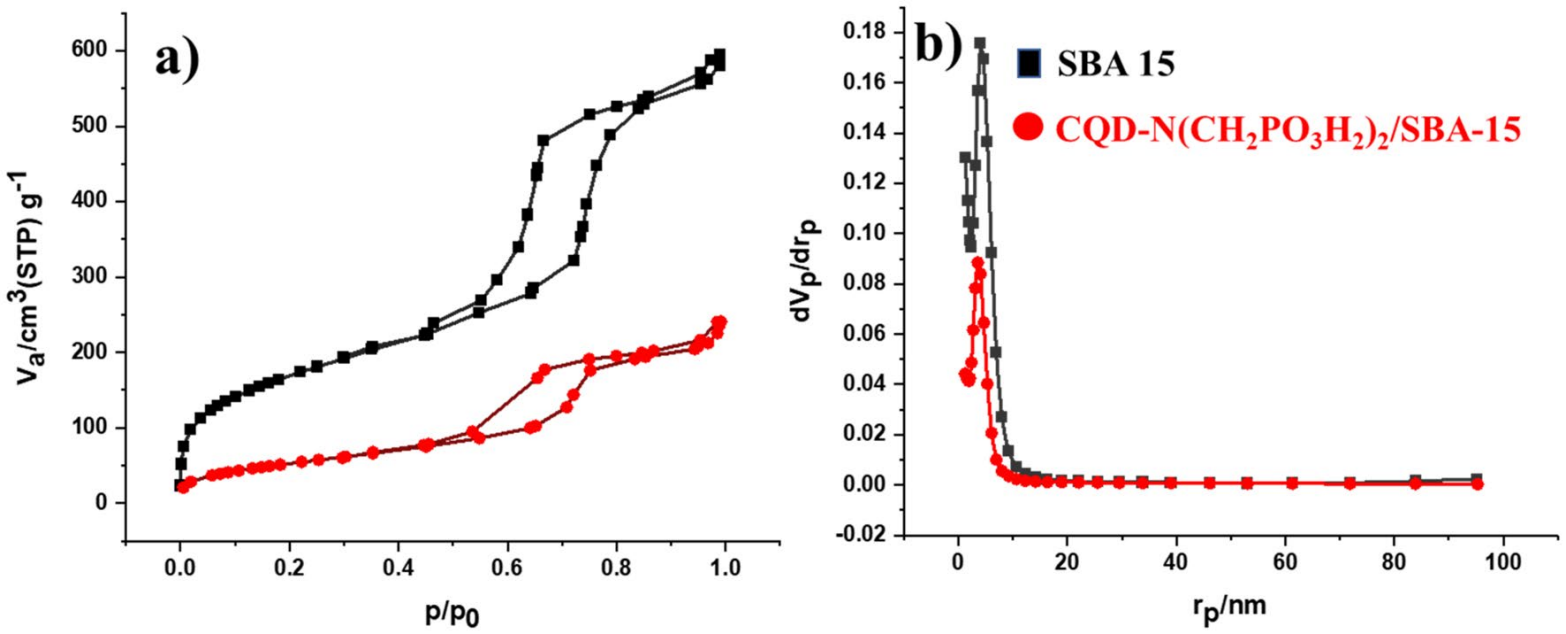
(**a**) Nitrogen adsorption–desorption isotherm and (**b**) BJH of SBA-15 and CQDs-N(CH_2_PO_3_H_2_)_2_/SBA-15.

The synthesis of CQDs-N(CH_2_PO_3_H_2_)_2_ encapsulated in SBA-15 as a composite material was also checked by XRD patterns of the corresponding starting material components (Figs. 5 and 6). For this purpose, XRD patterns exhibited distinguished three bands inclusive of a sharp band (100) and small bands indexed (110) and (200), which are in a close agreement with the previously reported data^18^. As shown in Fig. 5, the broad peak of CQDs-N(CH_2_PO_3_H_2_)_2_/SBA-15 corresponds to diffraction lines 002 in carbon quantum dots (CQDs). Therefore, the broad band in 2Ɵ = 15–35° indicated a successful immobilization of CQDs-N(CH_2_PO_3_H_2_)_2_ into SBA-15. The textural properties of SBA-15 and CQDs-N(CH_2_PO_3_H_2_)_2_/SBA-15 were also studied by *N*_*2*_ adsorption–desorption isotherms (Fig. 7a,b). A hysteresis loop is observed indicating the presence of mesoporous in the sample. The calculated surface areas for SBA-15 and catalyst based on BET equation and total pore volumes are 596.55 m^2^ g^−1^, 192.07 m^2^ g^−1^ and 0.8564 cm^3^ g^−1^, 0.3709 cm^3^ g^−1^_,_ respectively. The pore size distribution of CQDs-N(CH_2_PO_3_H_2_)_2_/SBA15 based on BJH method is shown in Fig. 7. This plot clearly shows pores diameter of SBA-15 was 4.03 nm and reached 3.53 in CQDs-N(CH_2_PO_3_H_2_)_2_/SBA-15.

**Figure 8:**
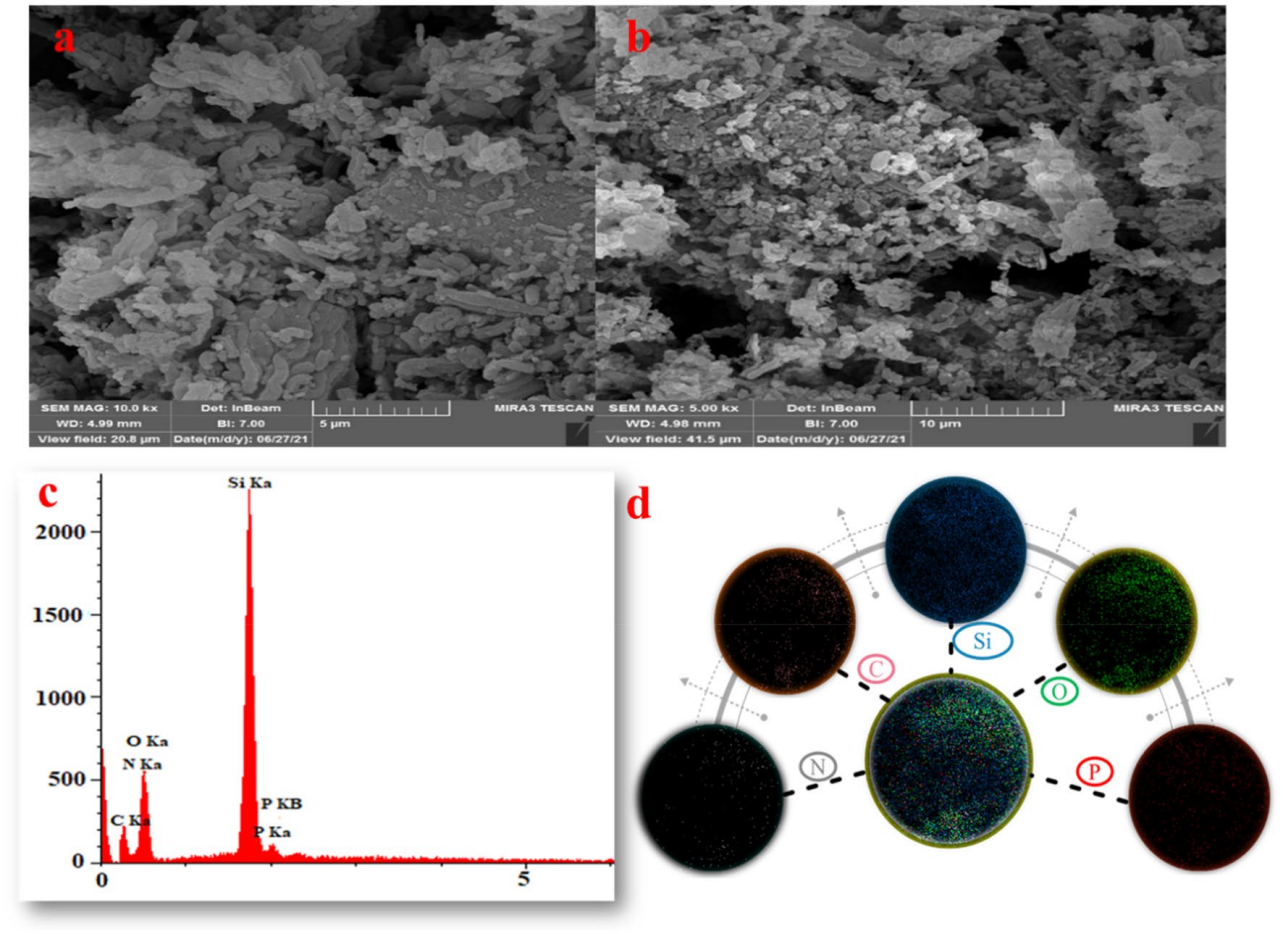
(**a**,**b**) Scanning electron microscope (SEM) images of CQDs-N(CH_2_PO_3_H_2_)_2_/SBA-15. (**c**) Energy dispersive X-ray analysis (EDX) and (**d**) SEM-elemental technique of CQDs-N(CH_2_PO_3_H_2_)_2_/SBA-15.

The surface of CQDs-N(CH_2_PO_3_H_2_)_2_/SBA-15 was determined by SEM images (Fig. 8). SEM images of the catalyst revealed that the particles have not completely agglomerated and particles of observed in tubular nano rod shape of silica template (Fig. 8a,b). In another investigation, the schaffold CQDs-N(CH_2_PO_3_H_2_)_2_/SBA-15 is composed of C, N, O, Si and P according to the energy dispersive X-ray spectroscopy (EDX) technique (Fig. 8c). Also, distribution of elements including P (red), Si (blue), O (green), N (yellow) and C (orange) on the surface of the catalyst was investigated and verified by SEM-elemental technique (Fig. 8d). Therefore, energy dispersive X-ray spectroscopy (EDX) and SEM-elemental mapping spectroscopy of all the expected elements confirm the uniform distribution of the elements on the surface.

**Figure 9:**
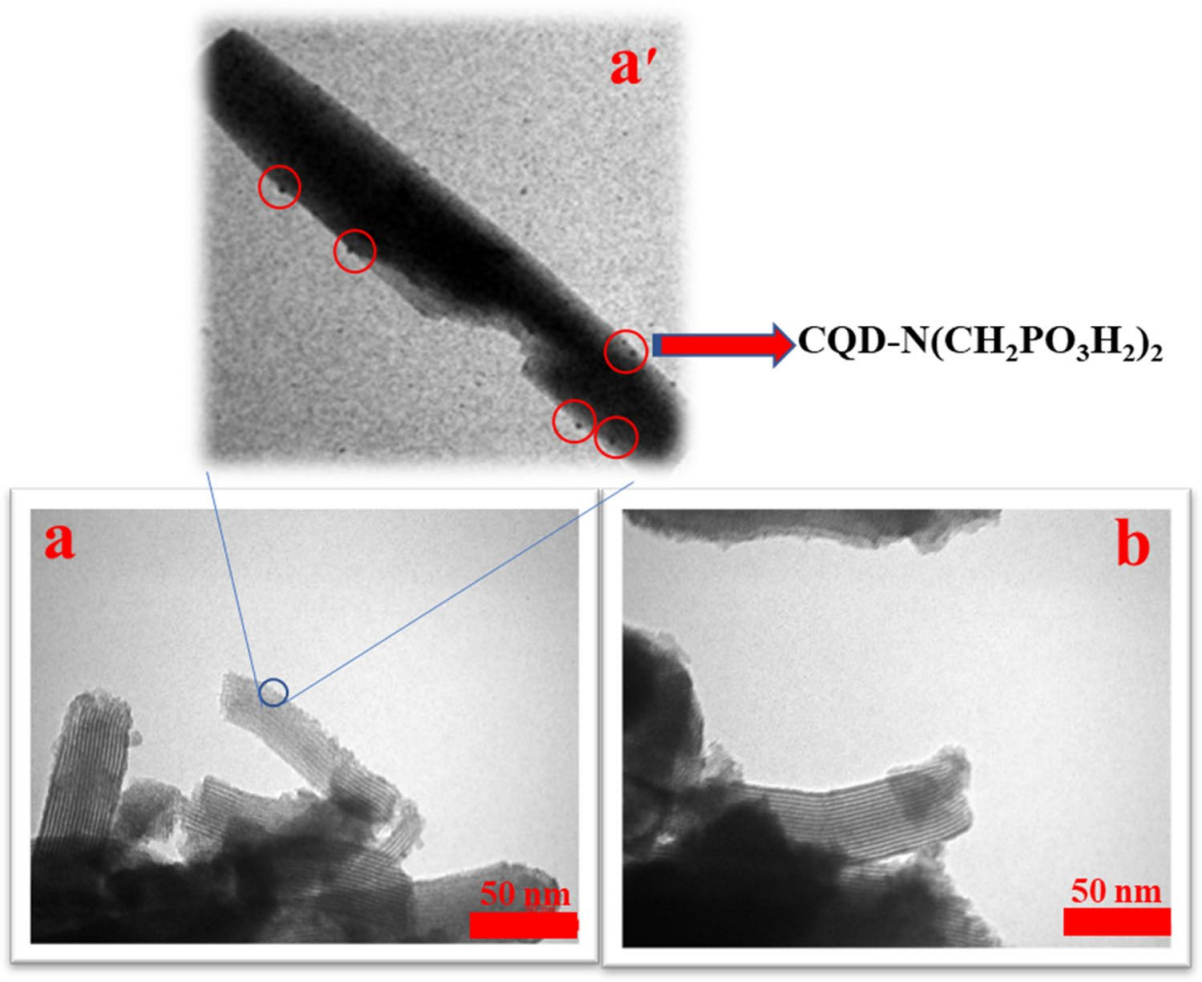
Transmission electron microcopy (TEM) images of CQDs-N(CH_2_PO_3_H_2_)_2_/SBA-15.

The morphology and surface of CQDs-N(CH_2_PO_3_H_2_)_2_/SBA-15 was determined by TEM analysis (Fig. 9). The TEM images of the CQDs-N(CH_2_PO_3_H_2_)_2_/SBA-15 were shown to be well fit with the two-dimensional hexagonal meso-structures. Based on these images, the scaffold of mesoporous template has been assembled with carbon quantum dots (CQDs) (Fig. 9), which agreed well with the results of low-angle XRD and *N*_*2*_ adsorption–desorption isotherms characterizations.

After successful synthesis and characterization of the CQDs-N(CH_2_PO_3_H_2_)_2_/SBA-15, it was used to prepare novel pyrazolo[4′,3′:5,6]pyrido[2,3-*d*]pyrimidine derivatives. The above-mentioned catalyst was achieved one-pot reaction of between 4-chloro benzaldehyde (1 mmol, 0.14 g), pyrimidine-2,4,6(1*H*,3*H*,5*H*)-trione (1 mmol, 0.128 g) and 5-(1*H*-indol-3-yl)-1*H*-pyrazol-3-amine (1 mmol, 0.198 g) as a model reaction. The model reaction was tested using different amounts of catalysts, solvents and temperatures. The results of the obtained products are summarized in Table 1. The best of choice for the synthesis of novel pyrazolo[4′,3′:5,6]pyrido[2,3-*d*]pyrimidines was achieved in the presence of catalytic amount of CQDs-N(CH_2_PO_3_H_2_)_2_/SBA-15 (10 mg) at 100 °C under the solvent-free condition (Entry 2, Table 1). The model reaction was also tested using different organic solvents such as such EtOH, DMF, H_2_O, CH_3_CN, *n*-Hexane, CHCl_3_, Toluene, MeOH, CH_2_Cl_2_, EtOAc (5 mL) and water which is results of the reaction did not improve) Entry 8–17, Table 1). The results show that CQDs-N(CH_2_PO_3_H_2_)_2_/SBA-15 is suitable for the preparation of novel pyrazolo[4′,3′:5,6]pyrido[2,3-*d*]pyrimidine derivatives.

**Table 1:** Effect of various amounts of catalysts, temperature and solvents in synthesis of pyrazolo[4′,3′:5,6]pyrido[2,3-*d*]pyrimidines.


Entry	Solvent	Catalyst (mg)	Temp. (˚C)	Time (min.)	Yield (%)
1	–	5	100	40	70
2	–	10	100	15	91
3	–	20	100	35	72
4	–	–	100	120	-
5	–	10	110	20	85
6	–	10	50	60	46
7	–	10	25	70	40
8	EtOH	10	Reflux	45	70
9	DMF	10	100	100	30
10	H_2_O	10	Reflux	120	–
11	CH_3_CN	10	Reflux	120	Trace
12	*n*-Hexane	10	Reflux	120	–
13	CHCl_3_	10	Reflux	100	35
14	Toluene	10	Reflux	120	–
15	MeOH	10	Reflux	60	60
16	CH_2_Cl_2_	10	Reflux	65	35
17	EtOAc	10	Reflux	120	–

After the optimization of the reaction conditions, the efficiency and applicability of CQDs-N(CH_2_PO_3_H_2_)_2_/SBA-15 were studied for the preparation of novel pyrazolo[4′,3′:5,6]pyrido[2,3-*d*]pyrimidines. The results are summarized in Tables 2 and 3. As Tables 2 and 3 indicate, pyrimidine-2,4,6(1*H*,3*H*,5*H*)-trione derivatives and 5-(1*H*-indol-3-yl)-1*H*-pyrazol-3-amine as starting materials and various aldehydes including bearing electron-donating, electron-withdrawing and halogens groups afforded desired products (b1-b15), (c1-c7) excellent yields (75–94%) and short reaction times (10–40 min.), respectively.

**Table 2:**
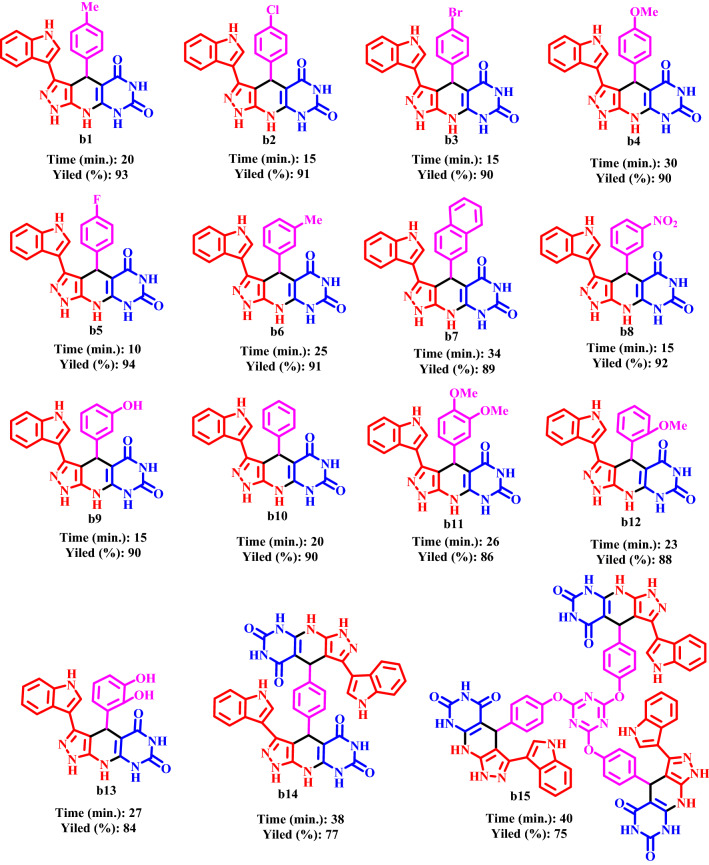
Synthesis of pyrazolo[4′,3′:5,6]pyrido[2,3-*d*]pyrimidines using CQDs–N(CH_2_PO_3_H_2_)_2_/SBA15 under solvent free condition.

**Table 3:**
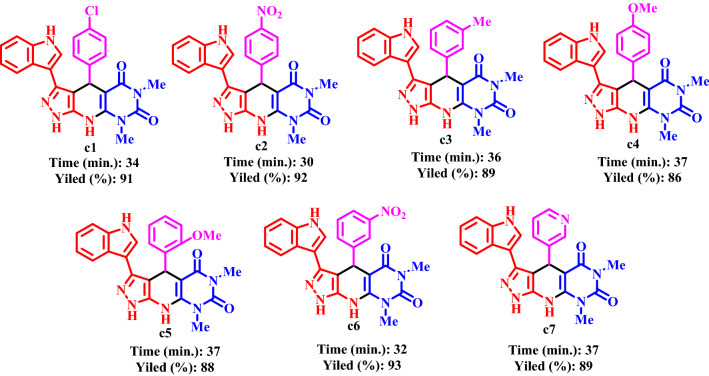
Synthesis of pyrazolo[4′,3′:5,6]pyrido[2,3-*d*]pyrimidines using CQDs-N(CH_2_PO_3_H_2_)_2_/SBA15 under solvent free condition.

**Figure 10:**
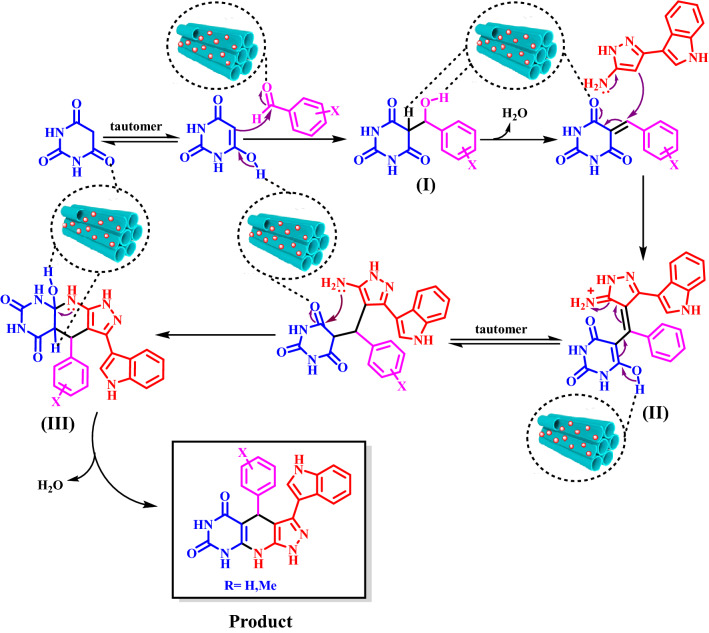
Proposed mechanism for the synthesis of pyrazolo[4′,3′:5,6]pyrido[2,3-*d*]pyrimidines.

In a suggested mechanism, the aldehyde is activated by the catalyst. The enol form of the pyrimidine-2,4,6(1*H*,3*H*,5*H*)-trione and activated aldehyde is produced intermediate **I**. Then, by reaction of intermediate **I** as a Michael acceptor and 5-(1*H*-indol-3-yl)-1*H*-pyrazol-3-amine leads to the intermediate **II**. In the following, intermediate **II** creates intermediate **III** during the intramolecular reaction and cyclization reaction. Finally, intermediate **III** loss of molecule H_2_O to prepared product (Fig. 10).

**Figure 11:**
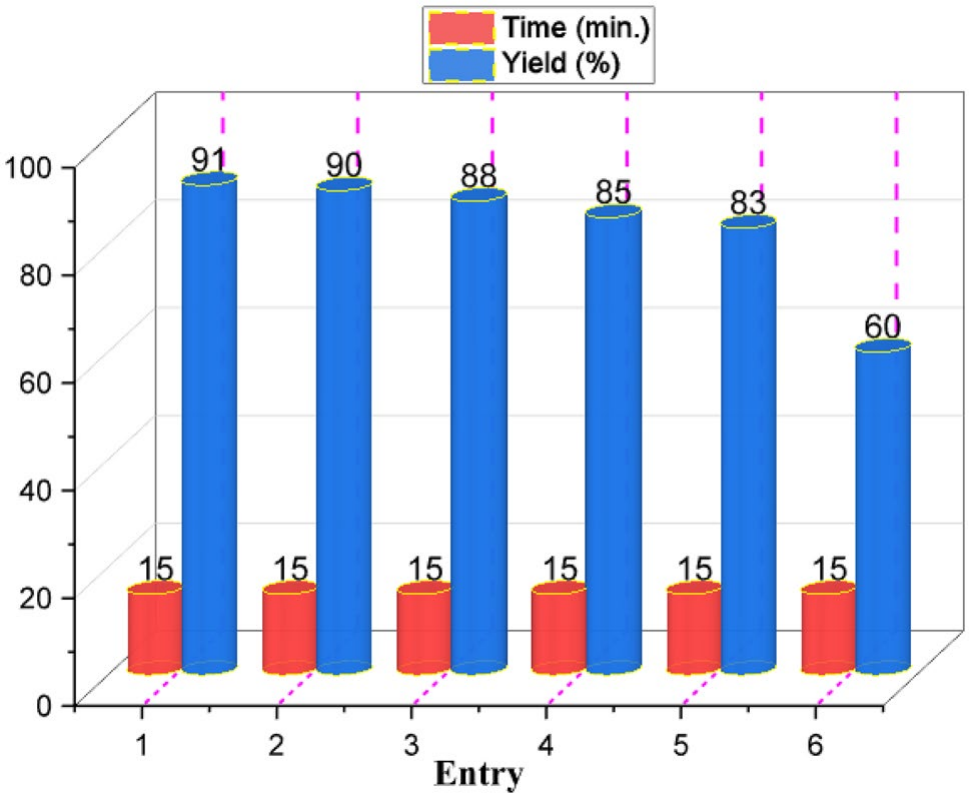
Recyclability of CQDs-N(CH_2_PO_3_H_2_)_2_/SBA-15 at the synthesis of pyrazolo[4′,3′:5,6]pyrido[2,3-*d*]pyrimidines.

In another section, to determine efficiency of CQDs-N(CH_2_PO_3_H_2_)_2_/SBA-15 as a catalyst, we have tested the model reaction using previously reported catalysts such as organic, magnetic, solid acid and basic catalysts. The results of the reactions showed that CQDs-N(CH_2_PO_3_H_2_)_2_/SBA-15 is the best catalyst for the synthesis of novel pyrazolo[4′,3′:5,6]pyrido[2,3-*d*]pyrimidine derivatives (short reaction time and high yield) (Table 4). Also, the recyclability and reusability of CQDs-N(CH_2_PO_3_H_2_)_2_/SBA-15 as catalyst were also studied on a model reaction under the above-mentioned reaction conditions. The results shown in Fig. 11, CQDs-N(CH_2_PO_3_H_2_)_2_/SBA-15 can be reused up to five runs in the reaction without a significant reduction in product yield.

**Table 4:** Synthesis of pyrazolo[4′,3′:5,6]pyrido[2,3-*d*]pyrimidines in the presence of various catalysts.


Entry	Catalyst	(mol%)	Time (min.)	Yield (%)
1	FeCl_3_	10	90	20
2	H_2_SO_4_	10	120	25
3	Fe_3_O_4_	10 mg	120	Trace
4	NH_4_NO_3_	10	100	20
5	CF_3_SO_3_H	10	80	25
6	GTBSA^29^	10	120	20
7	MIL-100(Cr)/NHEtN(CH_2_PO_3_H_2_)_2_^49^	10	90	45
8	H_3_[p(W_3_O_10_)_4_].XH_2_O	10	120	Trace
9	SBA-15/(CH_2_)_3_ N(CH_2_PO_3_H_2_)(CH_2_)_2_-N(CH_2_PO_3_H_2_)_2_^18^	10	60	52
10	[PVI-SO_3_H]FeCl_4_^50^	10	120	30
11	*p*-TSA	10	120	20
12	SSA^51^	10 mg	100	30
13	Et_3_N	10	120	–
14	MHMHPA^27,28^	10	100	30
15	[Py-SO_3_H]Cl^52^	10	120	20
16	APVPB^53^	10 mg	70	40
17	Fe_3_O_4_@Co(BDC)-NH_2_^54^	10 mg	85	45
18	H_3_PO_3_	10	70	55
19	CQDs^55^	10 mg	40	60
20	CQDs-N(CH_2_PO_3_H_2_)_2_^19^	10 mg	30	75
21	CQDs-N(CH_2_PO_3_H_2_)_2_/SBA-15	10 mg	15	91

A hot filtration test was conducted to synthesize 1,4-dihydropyridines (1,4-DHPs) as a model reaction under the optimized reaction condition. While running the fresh batch, the catalyst was filtered off at 25 min. At this stage, the reaction yield was 45%. Another reaction was run for 25 min and reaction mixture was filtered off. The filtrate was stirred for another 25 min under the same conditions. Incidentally, the reaction afforded no augmentation in its yield. Therefore, it can be concluded that the structure of the catalyst is not decomposed and the catalyst can be considered a heterogeneous catalyst.

## Conclusion

In summary, a novel porous composite catalyst consists of carbon quantum dots (CQDs) and the mesoporous silica namely CQDs-N(CH_2_PO_3_H_2_)_2_/SBA-15 was introduced and fully characterized by several techniques. This novel porous catalyst was investigated via a single step reaction for the synthesis of a wide range of novel pyrazolo[4′,3′:5,6]pyrido[2,3-*d*]pyrimidine derivatives as biological active candidates. The major advantages of described methodology are the high yield of the isolated products, short reaction time, eco-friendly way, mild and green reaction conditions.

## Supplementary Information

Supplementary Information.

## Data Availability

The datasets used and/or analyzed during the current study available from the corresponding author on reasonable request.
