# Supplementary material for: Phosphonic acid tagged carbon quantum dots encapsulated in SBA-15 as a novel catalyst for the preparation of N-heterocycles with pyrazolo, barbituric acid and indole moieties
Source: Sci Rep. 2022 Dec 2;12:20812. doi: 10.1038/s41598-022-24553-3 (PMC9718821; doi:10.1038/s41598-022-24553-3)
Supplement: Supplementary file 1 — Supplementary Information. [file 41598_2022_24553_MOESM1_ESM.docx]

**Phosphonic acid tagged carbon quantum dots encapsulated in SBA-15 as a novel catalyst for the preparation of *N*-heterocycles with pyrazolo,** **barbituric acid and indole moieties**

^a^ Department of Organic Chemistry, Faculty of Chemistry, Bu-Ali Sina University, Hamedan 6517838683, Tel: +988138282807, Fax: +988138380709 Iran. E-Mail: [zolfi@basu.ac.ir &](mailto:zolfi@basu.ac.ir%20&) [mzolfigol@yahoo.com](mailto:mzolfigol@yahoo.com) (M. A. Zolfigol).

^b^ Department of Chemistry, Faculty of Science, University of Qom, Qom 3716146611, Iran. E-Mail: [mahmoud8103@yahoo.com](mailto:mahmoud8103@yahoo.com) (M. Zarei).

^c^ Organic and Nano Group (ONG), Department of Chemistry Iran University of Science and Technology (IUST), PO Box 16846-13114, Tehran, Iran. E-Mail: rostamnia@iust.ac.ir.

**CONTENT**

*Spectral data of pyrazolo[4',3':5,6]pyrido[2,3-d]pyrimidines derivatives.....................................1*

[FT-IR spectrum of 3-(1H-indol-3-yl)-4-(p-tolyl)-1,4,8,9-tetrahydro-5H pyrazolo[4',3':5,6]pyrido[2,3-d]pyrimidine-5,7(6H)-dione (b1). 9](#_Toc73460070)

[^1^H-NMR spectrum of 3-(1H-indol-3-yl)-4-(p-tolyl)-1,4,8,9-tetrahydro-5H pyrazolo[4',3':5,6]pyrido[2,3-d]pyrimidine-5,7(6H)-dione (b1). 10](#_Toc73460071)

[^1^H-NMR (D_2_O exchange) spectrum of 3-(1H-indol-3-yl)-4-(p-tolyl)-1,4,8,9-tetrahydro-5H pyrazolo[4',3':5,6]pyrido[2,3-d]pyrimidine-5,7(6H)-dione (b1). 11](#_Toc73460073)

[^13^C-NMR spectrum of 3-(1H-indol-3-yl)-4-(p-tolyl)-1,4,8,9-tetrahydro-5H pyrazolo[4',3':5,6]pyrido[2,3-d]pyrimidine-5,7(6H)-dione (b1). 12](#_Toc73460072)

[FT-IR spectrum of 4-(4-chlorophenyl)-3-(1H-indol-3-yl)-1,4,8,9-tetrahydro-5H-pyrazolo[4',3':5,6]pyrido[2,3-d]pyrimidine-5,7(6H)-dione (b2). 13](#_Toc73460077)

[^1^H-NMR spectrum of 4-(4-chlorophenyl)-3-(1H-indol-3-yl)-1,4,8,9-tetrahydro-5H-pyrazolo[4',3':5,6]pyrido[2,3-d]pyrimidine-5,7(6H)-dione (b2). 14](#_Toc73460078)

[^13^C-NMR spectrum of 4-(4-chlorophenyl)-3-(1H-indol-3-yl)-1,4,8,9-tetrahydro-5H-pyrazolo[4',3':5,6]pyrido[2,3-d]pyrimidine-5,7(6H)-dione (b2). 15](#_Toc73460079)

[FT-IR spectrum of 4-(4-bromophenyl)-3-(1H-indol-3-yl)-1,4,8,9-tetrahydro-5H-pyrazolo[4',3':5,6]pyrido[2,3-d]pyrimidine-5,7(6H)-dione (b3). 16](#_Toc73460080)

[^1^H-NMR spectrum of 4-(4-bromophenyl)-3-(1H-indol-3-yl)-1,4,8,9-tetrahydro-5H-pyrazolo[4',3':5,6]pyrido[2,3-d]pyrimidine-5,7(6H)-dione (b3). 17](#_Toc73460081)

[^13^C-NMR spectrum of 4-(4-bromophenyl)-3-(1H-indol-3-yl)-1,4,8,9-tetrahydro-5H-pyrazolo[4',3':5,6]pyrido[2,3-d]pyrimidine-5,7(6H)-dione (b3). 18](#_Toc73460082)

[FT-IR spectrum of 3-(1H-indol-3-yl)-4-(4-methoxyphenyl)-1,4,8,9-tetrahydro-5H-pyrazolo[4',3':5,6]pyrido[2,3-d]pyrimidine-5,7(6H)-dione (b4). 19](#_Toc73460083)

[^1^H-NMR spectrum of 3-(1H-indol-3-yl)-4-(4-methoxyphenyl)-1,4,8,9-tetrahydro-5H-pyrazolo[4',3':5,6]pyrido[2,3-d]pyrimidine-5,7(6H)-dione (b4). 20](#_Toc73460084)

[^13^C-NMR spectrum of 3-(1H-indol-3-yl)-4-(4-methoxyphenyl)-1,4,8,9-tetrahydro-5H-pyrazolo[4',3':5,6]pyrido[2,3-d]pyrimidine-5,7(6H)-dione (b4). 21](#_Toc73460085)

[FT-IR spectrum of 4-(4-fluorophenyl)-3-(1H-indol-3-yl)-1,4,8,9-tetrahydro-5H-pyrazolo[4',3':5,6]pyrido[2,3-d]pyrimidine-5,7(6H)-dione (b5). 22](#_Toc73460086)

[^1^H-NMR spectrum of 4-(4-fluorophenyl)-3-(1H-indol-3-yl)-1,4,8,9-tetrahydro-5H-pyrazolo[4',3':5,6]pyrido[2,3-d]pyrimidine-5,7(6H)-dione (b5). 23](#_Toc73460087)

[^13^C-NMR spectrum of 4-(4-fluorophenyl)-3-(1H-indol-3-yl)-1,4,8,9-tetrahydro-5H-pyrazolo[4',3':5,6]pyrido[2,3-d]pyrimidine-5,7(6H)-dione (b5). 24](#_Toc73460088)

[FT-IR spectrum of 3-(1H-indol-3-yl)-4-(m-tolyl)-1,4,8,9-tetrahydro-5H-pyrazolo[4',3':5,6]pyrido[2,3-d]pyrimidine-5,7(6H)-dione (b6). 25](#_Toc73460089)

[^1^H-NMR spectrum of 3-(1H-indol-3-yl)-4-(m-tolyl)-1,4,8,9-tetrahydro-5H-pyrazolo[4',3':5,6]pyrido[2,3-d]pyrimidine-5,7(6H)-dione (b6). 26](#_Toc73460090)

[^13^C-NMR spectrum of 3-(1H-indol-3-yl)-4-(m-tolyl)-1,4,8,9-tetrahydro-5H-pyrazolo[4',3':5,6]pyrido[2,3-d]pyrimidine-5,7(6H)-dione (b6). 27](#_Toc73460091)

[FT-IR spectrum of 3-(1H-indol-3-yl)-4-(naphthalen-2-yl)-1,4,8,9-tetrahydro-5H-pyrazolo[4',3':5,6]pyrido[2,3-d]pyrimidine-5,7(6H)-dione (b7). 28](#_Toc73460092)

[^1^H-NMR spectrum 3-(1H-indol-3-yl)-4-(naphthalen-2-yl)-1,4,8,9-tetrahydro-5H-pyrazolo[4',3':5,6]pyrido[2,3-d]pyrimidine-5,7(6H)-dione (b7). 29](#_Toc73460093)

[^13^C-NMR spectrum of 3-(1H-indol-3-yl)-4-(naphthalen-2-yl)-1,4,8,9-tetrahydro-5H-pyrazolo[4',3':5,6]pyrido[2,3-d]pyrimidine-5,7(6H)-dione (b7). 30](#_Toc73460094)

[FT-IR spectrum of 3-(1H-indol-3-yl)-4-(3-nitrophenyl)-1,4,8,9-tetrahydro-5H-pyrazolo[4',3':5,6]pyrido[2,3-d]pyrimidine-5,7(6H)-dione (b8). 31](#_Toc73460095)

[^1^H-NMR spectrum of 3-(1H-indol-3-yl)-4-(3-nitrophenyl)-1,4,8,9-tetrahydro-5H-pyrazolo[4',3':5,6]pyrido[2,3-d]pyrimidine-5,7(6H)-dione (b8). 32](#_Toc73460096)

[^13^C-NMR spectrum of 3-(1H-indol-3-yl)-4-(3-nitrophenyl)-1,4,8,9-tetrahydro-5H-pyrazolo[4',3':5,6]pyrido[2,3-d]pyrimidine-5,7(6H)-dione (b8). 33](#_Toc73460097)

[FT-IR spectrum of 4-(3-hydroxyphenyl)-3-(1H-indol-3-yl)-1,4,8,9-tetrahydro-5H-pyrazolo[4',3':5,6]pyrido[2,3-d]pyrimidine-5,7(6H)-dione (b9). 34](#_Toc73460098)

[^1^H-NMR spectrum of 4-(3-hydroxyphenyl)-3-(1H-indol-3-yl)-1,4,8,9-tetrahydro-5H-pyrazolo[4',3':5,6]pyrido[2,3-d]pyrimidine-5,7(6H)-dione (b9). 35](#_Toc73460099)

[^13^C-NMR spectrum of 4-(3-hydroxyphenyl)-3-(1H-indol-3-yl)-1,4,8,9-tetrahydro-5H-pyrazolo[4',3':5,6]pyrido[2,3-d]pyrimidine-5,7(6H)-dione (b9). 36](#_Toc73460100)

[FT-IR spectrum of 3-(1H-indol-3-yl)-4-phenyl-1,4,8,9-tetrahydro-5H-pyrazolo[4',3':5,6]pyrido[2,3-d]pyrimidine-5,7(6H)-dione (b10). 37](#_Toc73460101)

[^1^H-NMR spectrum of 3-(1H-indol-3-yl)-4-phenyl-1,4,8,9-tetrahydro-5H-pyrazolo[4',3':5,6]pyrido[2,3-d]pyrimidine-5,7(6H)-dione (b10). 38](#_Toc73460102)

[^13^C-NMR spectrum of 3-(1H-indol-3-yl)-4-phenyl-1,4,8,9-tetrahydro-5H-pyrazolo[4',3':5,6]pyrido[2,3-d]pyrimidine-5,7(6H)-dione (b10). 39](#_Toc73460103)

[FT-IR spectrum 4-(2,3-dihydroxyphenyl)-3-(1H-indol-3-yl)-1,4,8,9-tetrahydro-5H-pyrazolo[4',3':5,6]pyrido[2,3-d]pyrimidine-5,7(6H)-dione (b11).. 40](#_Toc73460104)

[^1^H-NMR spectrum 4-(2,3-dihydroxyphenyl)-3-(1H-indol-3-yl)-1,4,8,9-tetrahydro-5H-pyrazolo[4',3':5,6]pyrido[2,3-d]pyrimidine-5,7(6H)-dione (b11).. 41](#_Toc73460105)

[^13^C-NMR spectrum of 4-(2,3-dihydroxyphenyl)-3-(1H-indol-3-yl)-1,4,8,9-tetrahydro-5H-pyrazolo[4',3':5,6]pyrido[2,3-d]pyrimidine-5,7(6H)-dione (b11).. 42](#_Toc73460106)

[FT-IR spectrum of 3-(1H-indol-3-yl)-4-(2-methoxyphenyl)-1,4,8,9-tetrahydro-5H-pyrazolo[4',3':5,6]pyrido[2,3-d]pyrimidine-5,7(6H)-dione (b12). 43](#_Toc73460107)

[^1^H-NMR spectrum of 3-(1H-indol-3-yl)-4-(2-methoxyphenyl)-1,4,8,9-tetrahydro-5H-pyrazolo[4',3':5,6]pyrido[2,3-d]pyrimidine-5,7(6H)-dione (b12). 44](#_Toc73460108)

[^13^C-NMR spectrum of 3-(1H-indol-3-yl)-4-(2-methoxyphenyl)-1,4,8,9-tetrahydro-5H-pyrazolo[4',3':5,6]pyrido[2,3-d]pyrimidine-5,7(6H)-dione (b12).. 45](#_Toc73460106)

[FT-IR spectrum of 4-(2,3-dihydroxyphenyl)-3-(1H-indol-3-yl)-1,4,8,9-tetrahydro-5H-pyrazolo[4',3':5,6]pyrido[2,3-d]pyrimidine-5,7(6H)-dione (b13). 46](#_Toc73460109)

[^1^H-NMR spectrum of 4-(2,3-dihydroxyphenyl)-3-(1H-indol-3-yl)-1,4,8,9-tetrahydro-5H-pyrazolo[4',3':5,6]pyrido[2,3-d]pyrimidine-5,7(6H)-dione (b13). 47](#_Toc73460110)

[^13^C-NMR spectrum of 4-(2,3-dihydroxyphenyl)-3-(1H-indol-3-yl)-1,4,8,9-tetrahydro-5H-pyrazolo[4',3':5,6]pyrido[2,3-d]pyrimidine-5,7(6H)-dione (b13). 48](#_Toc73460111)

[FT-IR spectrum of 4,4'-(1,4-phenylene)bis(3-(1H-indol-3-yl)-1,4,8,9-tetrahydro-5H-pyrazolo[4',3':5,6]pyrido[2,3-d]pyrimidine-5,7(6H)-dione) (b14). 49](#_Toc73460112)

[^1^H-NMR spectrum of 4,4'-(1,4-phenylene)bis(3-(1H-indol-3-yl)-1,4,8,9-tetrahydro-5H-pyrazolo[4',3':5,6]pyrido[2,3-d]pyrimidine-5,7(6H)-dione) (b14). 50](#_Toc73460113)

[^13^C-NMR spectrum 4,4'-(1,4-phenylene)bis(3-(1H-indol-3-yl)-1,4,8,9-tetrahydro-5H-pyrazolo[4',3':5,6]pyrido[2,3-d]pyrimidine-5,7(6H)-dione) (b14). 51](#_Toc73460114)

[FT-IR spectrum 4,4',4''-(((1,3,5-triazine-2,4,6-triyl)tris(oxy))tris(benzene-4,1-diyl))tris(3-(1H-indol-3-yl)-1,4,8,9-tetrahydro-5H-pyrazolo[4',3':5,6]pyrido[2,3-d]pyrimidine-5,7(6H)-dione) (b15). 52](#_Toc73460115)

[^1^H-NMR spectrum 4,4',4''-(((1,3,5-triazine-2,4,6-triyl)tris(oxy))tris(benzene-4,1-diyl))tris(3-(1H-indol-3-yl)-1,4,8,9-tetrahydro-5H-pyrazolo[4',3':5,6]pyrido[2,3-d]pyrimidine-5,7(6H)-dione) (b15). 53](#_Toc73460116)

[^13^C-NMR spectrum of 4,4',4''-(((1,3,5-triazine-2,4,6-triyl)tris(oxy))tris(benzene-4,1-diyl))tris(3-(1H-indol-3-yl)-1,4,8,9-tetrahydro-5H-pyrazolo[4',3':5,6]pyrido[2,3-d]pyrimidine-5,7(6H)-dione) (b15). 54](#_Toc73460117)

[FT-IR spectrum of 4-(4-chlorophenyl)-3-(1H-indol-3-yl)-6,8-dimethyl-1,4,8,9-tetrahydro-5H-pyrazolo[4',3':5,6]pyrido[2,3-d]pyrimidine-5,7(6H)-dione (c1). 55](#_Toc73460118)

[^1^H-NMR spectrum of 4-(4-chlorophenyl)-3-(1H-indol-3-yl)-6,8-dimethyl-1,4,8,9-tetrahydro-5H-pyrazolo[4',3':5,6]pyrido[2,3-d]pyrimidine-5,7(6H)-dione (c1). 56](#_Toc73460119)

[^13^C-NMR spectrum of 4-(4-chlorophenyl)-3-(1H-indol-3-yl)-6,8-dimethyl-1,4,8,9-tetrahydro-5H-pyrazolo[4',3':5,6]pyrido[2,3-d]pyrimidine-5,7(6H)-dione (c1). 57](#_Toc73460120)

[FT-IR spectrum of 3-(1H-indol-3-yl)-6,8-dimethyl-4-(4-nitrophenyl)-1,4,8,9-tetrahydro-5H-pyrazolo[4',3':5,6]pyrido[2,3-d]pyrimidine-5,7(6H)-dione (c2). 58](#_Toc73460121)

[^1^H-NMR spectrum of 3-(1H-indol-3-yl)-6,8-dimethyl-4-(4-nitrophenyl)-1,4,8,9-tetrahydro-5H-pyrazolo[4',3':5,6]pyrido[2,3-d]pyrimidine-5,7(6H)-dione (c2). 59](#_Toc73460122)

[^13^C-NMR spectrum of 3-(1H-indol-3-yl)-6,8-dimethyl-4-(4-nitrophenyl)-1,4,8,9-tetrahydro-5H-pyrazolo[4',3':5,6]pyrido[2,3-d]pyrimidine-5,7(6H)-dione (c2). 60](#_Toc73460123)

[FT-IR spectrum of 3-(1H-indol-3-yl)-6,8-dimethyl-4-(m-tolyl)-1,4,8,9-tetrahydro-5H-pyrazolo[4',3':5,6]pyrido[2,3-d]pyrimidine-5,7(6H)-dione (c3). 61](#_Toc73460124)

[^1^H-NMR spectrum of 3-(1H-indol-3-yl)-6,8-dimethyl-4-(m-tolyl)-1,4,8,9-tetrahydro-5H-pyrazolo[4',3':5,6]pyrido[2,3-d]pyrimidine-5,7(6H)-dione (c3). 62](#_Toc73460125)

[^13^C-NMR spectrum of 3-(1H-indol-3-yl)-6,8-dimethyl-4-(m-tolyl)-1,4,8,9-tetrahydro-5H-pyrazolo[4',3':5,6]pyrido[2,3-d]pyrimidine-5,7(6H)-dione (c3). 63](#_Toc73460126)

[FT-IR spectrum of 3-(1H-indol-3-yl)-4-(4-methoxyphenyl)-6,8-dimethyl-1,4,8,9-tetrahydro-5H-pyrazolo[4',3':5,6]pyrido[2,3-d]pyrimidine-5,7(6H)-dione (c4).. 64](#_Toc73460127)

[^1^H-NMR spectrum of 3-(1H-indol-3-yl)-4-(4-methoxyphenyl)-6,8-dimethyl-1,4,8,9-tetrahydro-5H-pyrazolo[4',3':5,6]pyrido[2,3-d]pyrimidine-5,7(6H)-dione (c4). 65](#_Toc73460128)

[^13^C-NMR spectrum of 3-(1H-indol-3-yl)-4-(4-methoxyphenyl)-6,8-dimethyl-1,4,8,9-tetrahydro-5H-pyrazolo[4',3':5,6]pyrido[2,3-d]pyrimidine-5,7(6H)-dione (c4).. 66](#_Toc73460129)

[FT-IR spectrum of 3-(1H-indol-3-yl)-4-(2-methoxyphenyl)-6,8-dimethyl-1,4,8,9-tetrahydro-5H-pyrazolo[4',3':5,6]pyrido[2,3-d]pyrimidine-5,7(6H)-dione (c5). 67](#_Toc73460130)

[^1^H-NMR spectrum of 3-(1H-indol-3-yl)-4-(2-methoxyphenyl)-6,8-dimethyl-1,4,8,9-tetrahydro-5H-pyrazolo[4',3':5,6]pyrido[2,3-d]pyrimidine-5,7(6H)-dione (c5). 68](#_Toc73460131)

[^13^C-NMR spectrum of 3-(1H-indol-3-yl)-4-(2-methoxyphenyl)-6,8-dimethyl-1,4,8,9-tetrahydro-5H-pyrazolo[4',3':5,6]pyrido[2,3-d]pyrimidine-5,7(6H)-dione (c5). 69](#_Toc73460132)

[FT-IR spectrum of 3-(1H-indol-3-yl)-6,8-dimethyl-4-(3-nitrophenyl)-1,4,8,9-tetrahydro-5H-pyrazolo[4',3':5,6]pyrido[2,3-d]pyrimidine-5,7(6H)-dione (c6). 70](#_Toc73460133)

[^1^H-NMR spectrum of 3-(1H-indol-3-yl)-6,8-dimethyl-4-(3-nitrophenyl)-1,4,8,9-tetrahydro-5H-pyrazolo[4',3':5,6]pyrido[2,3-d]pyrimidine-5,7(6H)-dione (c6). 71](#_Toc73460134)

[FT-IR spectrum of 3-(1H-indol-3-yl)-6,8-dimethyl-4-(pyridin-3-yl)-1,4,8,9-tetrahydro-5H-pyrazolo[4',3':5,6]pyrido[2,3-d]pyrimidine-5,7(6H)-dione (c7). 72](#_Toc73460136)

[^1^H-NMR spectrum of 3-(1H-indol-3-yl)-6,8-dimethyl-4-(pyridin-3-yl)-1,4,8,9-tetrahydro-5H-pyrazolo[4',3':5,6]pyrido[2,3-d]pyrimidine-5,7(6H)-dione (c7). 73](#_Toc73460137)

[^13^C-NMR spectrum of 3-(1H-indol-3-yl)-6,8-dimethyl-4-(pyridin-3-yl)-1,4,8,9-tetrahydro-5H-pyrazolo[4',3':5,6]pyrido[2,3-d]pyrimidine-5,7(6H)-dione (c7). 74](#_Toc73460138)

**Spectral data of pyrazolo[4',3':5,6]pyrido[2,3-*d*]pyrimidines derivatives**

***3-(1H-indol-3-yl)-4-(p-tolyl)-1,4,8,9-tetrahydro-5H pyrazolo[4',3':5,6]pyrido[2,3-d]pyrimidine-5,7(6H)-dione (b1)***

Yellow solid; Mp: >300 ˚C; FT-IR (KBr, cm^-1^): 3396, 3340, 3248, 1713, 1659, 1513. ^1^H NMR (400 MHz, DMSO-*d*_6_) δ 12.35 (s, 1H), 11.50 (s, 1H), 10.63 (s, 1H), 10.10 (s, 1H), 9.03 (s, 1H), 7.80 (d, *J* = 7.9 Hz, 1H), 7.51 (d, *J* = 8.1 Hz, 1H), 7.36 (d, *J* = 2.7 Hz, 1H), 7.26 (t, *J* = 7.5 Hz, 1H), 7.19 (t, *J* = 7.5 Hz, 1H), 7.07 (d, *J* = 8.1 Hz, 2H), 7.00 (d, *J* = 8.1 Hz, 2H), 5.22 (s, 1H), 2.25 (s, 3H). ^13^C NMR (101 MHz, DMSO-*d*_6_) δ 162.8, 149.9, 146.5, 146.3, 144.1, 135.8, 134.4, 134.2, 128.2, 127.2, 124.6, 124.5, 121.7, 119.6, 119.5, 111.8, 104.3, 101.1, 87.5, 34.3, 20.5. ^1^H NMR (400 MHz, DMSO-*d*_6_ and D_2_O) δ 7.64 (d, *J* = 7.7 Hz, 1H), 7.41 (d, *J* = 8.1 Hz, 1H), 7.25 (s, 1H), 7.16 (t, *J* = 7.5 Hz, 1H), 7.08 (t, *J* = 7.4 Hz, 1H), 6.93 – 6.85 (m, 4H), 5.10 (s, 1H), 2.10 (s, 3H).

***4-(4-Chlorophenyl)-3-(1H-indol-3-yl)-1,4,8,9-tetrahydro-5H-pyrazolo[4',3':5,6]pyrido[2,3-d]pyrimidine-5,7(6H)-dione (b2)***

White solid; Mp: >300 ˚C; FT-IR (KBr, cm^-1^): 3404, 3202, 1710, 1632, 1569, 1475 .^1^H NMR (400 MHz, DMSO-*d*_6_) δ 12.30 (s, 1H), 11.42 (s, 1H), 10.59 (s, 1H), 10.08 (s, 1H), 9.03 (s, 1H), 7.65 (d, *J* = 7.9 Hz, 1H), 7.40 (d, *J* = 8.1 Hz, 1H), 7.29 (d, *J* = 2.8 Hz, 1H), 7.17 – 7.11 (m, 3H), 7.08 – 7.01 (m, 3H), 5.17 (s, 1H). ^13^C NMR (101 MHz, DMSO-*d*_6_) δ 162.9, 149.9, 146.5, 145.9, 135.8, 134.5, 130.0, 129.1, 127.5, 124.7, 124.6, 121.8, 119.6, 119.4, 111.8, 104.1, 100.6, 86.8, 34.4.

***4-(4-Bromophenyl)-3-(1H-indol-3-yl)-1,4,8,9-tetrahydro-5H-pyrazolo[4',3':5,6]pyrido[2,3-d]pyrimidine-5,7(6H)-dione (b3)***

White solid; Mp: >300 ˚C; FT-IR (KBr, cm^-1^): 3421, 3329, 3195, 1708, 1606, 1423. ^1^H NMR (400 MHz, DMSO-*d*_6_) δ 12.40 (s, 1H), 11.52 (s, 1H), 10.68 (s, 1H), 10.17 (s, 1H), 9.12 (s, 1H), 7.77 (d, *J* = 7.8 Hz, 1H), 7.51 (d, *J* = 8.1 Hz, 1H), 7.40 (d, *J* = 2.7 Hz, 1H), 7.38 – 7.34 (m, 2H), 7.26 (t, 1H), 7.18 (t, 1H), 7.10 (d, *J* = 8.5 Hz, 2H), 5.26 (s, 1H). ^13^C NMR (101 MHz, DMSO-*d*_6_) δ 162.9, 149.9, 146.5, 146.3, 135.8, 134.4, 130.4, 129.6, 124.7, 124.6, 121.8, 119.6, 119.4, 118.6, 111.8, 104.1, 100.4, 86.7, 34.5.

***3-(1H-Indol-3-yl)-4-(4-methoxyphenyl)-1,4,8,9-tetrahydro-5H-pyrazolo[4',3':5,6]pyrido[2,3-d]pyrimidine-5,7(6H)-dione (b4)***

Yellow solid; Mp: >300 ˚C; FT-IR (KBr, cm^-1^): 3362, 3278, 3162, 1728, 1686, 1551. ^1^H NMR (400 MHz, DMSO-*d*_6_) δ 12.26 (s, 1H), 11.40 (s, 1H), 10.56 (s, 1H), 10.05 (s, 1H), 8.96 (s, 1H), 7.71 (d, *J* = 7.9 Hz, 1H), 7.41 (d, *J* = 8.1 Hz, 1H), 7.24 (d, *J* = 2.7 Hz, 1H), 7.18 – 7.13 (m, 1H), 7.10 – 7.06 (m, 1H), 7.00 (d, *J* = 8.9 Hz, 2H), 6.66 (d, *J* = 8.9 Hz, 2H), 5.10 (s, 1H), 3.61 (s, 3H). ^13^C NMR (101 MHz, DMSO-*d*_6_) δ 162.9, 157.1, 149.9, 146.2, 139.3, 135.8, 128.3, 124.6, 124.5, 121.7, 119.6, 113.0, 111.7, 104.4, 101.1, 87.6, 54.8, 33.9.

***4-(4-Fluorophenyl)-3-(1H-indol-3-yl)-1,4,8,9-tetrahydro-5H-pyrazolo[4',3':5,6]pyrido[2,3-d]pyrimidine-5,7(6H)-dione (b5)***

White solid; Mp: >300 ˚C; FT-IR (KBr, cm^-1^): 3392, 3229, 3200, 1710, 1599, 1523. ^1^H NMR (400 MHz, DMSO-*d*_6_) δ 12.38 (s, 1H), 11.50 (s, 1H), 10.65 (s, 1H), 10.14 (s, 1H), 9.12 (s, 1H), 7.78 (d, *J* = 8.6 Hz, 1H), 7.52 (d, *J* = 8.2 Hz, 1H), 7.37 (d, *J* = 2.7 Hz, 1H), 7.29 – 7.24 (m, 1H), 7.21 – 7.15 (m, 3H), 7.00 (t, *J* = 8.9 Hz, 2H), 5.28 (s, 1H). ^13^C NMR (101 MHz, DMSO-*d*_6_) δ 162.9, 161.4, 159.0, 143.2, 143.2, 135.8, 129.0, 129.0, 124.7, 124.5, 121.7, 119.6, 119.4, 114.3, 114.1, 111.8, 100.9, 87.1 34.12.

***3-(1H-Indol-3-yl)-4-(m-tolyl)-1,4,8,9-tetrahydro-5H-pyrazolo[4',3':5,6]pyrido[2,3-d]pyrimidine-5,7(6H)-dione (b6)***

Yellow solid; Mp: >300 ˚C; FT-IR (KBr, cm^-1^): 3402, 3331, 3228, 1713, 1659, 1512. ^1^H NMR (400 MHz, DMSO-*d*_6_) δ 12.33 (s, 1H), 11.51 (s, 1H), 10.64 (s, 1H), 10.14 (s, 1H), 9.05 (s, 1H), 7.77 (d, *J* = 7.9 Hz, 1H), 7.52 (d, *J* = 8.1 Hz, 1H), 7.34 (d, *J* = 2.7 Hz, 1H), 7.26 (t, *J* = 7.6 Hz, 1H), 7.18 (t, *J* = 7.5 Hz, 1H), 7.07 (t, *J* = 7.8 Hz, 1H), 6.97 – 6.90 (m, 3H), 5.20 (s, 1H), 2.19 (s, 3H). ^13^C NMR (101 MHz, DMSO-*d*_6_) δ 162.8, 149.9, 146.9, 146.5, 146.2, 136.4, 135.8, 127.9, 127.5, 126.3, 124.8, 124.5, 124.5, 121.7, 119.6, 119.5, 111.7, 104.4, 101.1, 87.2, 34.7, 21.0.

***3-(1H-Indol-3-yl)-4-(naphthalen-2-yl)-1,4,8,9-tetrahydro-5H-pyrazolo[4',3':5,6]pyrido[2,3-d]pyrimidine-5,7(6H)-dione (b7)***

Yellow solid; Mp: >300 ˚C; FT-IR (KBr, cm^-1^): 3409, 3375, 3249, 1714, 1659, 1606. ^1^H NMR (400 MHz, DMSO-*d*_6_) δ 12.38 (s, 1H), 11.47 (s, 1H), 10.65 (d, *J* = 1.7 Hz, 1H), 10.19 (s, 1H), 9.14 (s, 1H), 7.84 – 7.71 (m, 4H), 7.64 (s, 1H), 7.50 – 7.45 (m, 3H), 7.36 (d, *J* = 2.7 Hz, 1H), 7.33 (d, *J* = 8.5 Hz, 1H), 7.23 (t, *J* = 6.9 Hz, 1H), 7.15 (d, *J* = 6.9 Hz, 1H), 5.43 (s, 1H). ^13^C NMR (101 MHz, DMSO-*d*_6_) δ 162.9, 149.9, 146.6, 144.2, 135.8, 134.6, 132.5, 131.5, 127.4, 127.2, 127.2, 126.4, 125.7, 125.2, 125.1, 124.7, 124.6, 121.7, 119.6, 119.5, 111.7, 104.2, 100.8, 87.0, 35.1.

***3-(1H-Indol-3-yl)-4-(3-nitrophenyl)-1,4,8,9-tetrahydro-5H-pyrazolo[4',3':5,6]pyrido[2,3-d]pyrimidine-5,7(6H)-dione (b8)***

Brown solid; Mp: >300 ˚C; FT-IR (KBr, cm^-1^): 3377, 3203, 1709, 1654, 1524, 1499. ^1^H NMR (400 MHz, DMSO-*d*_6_) δ 12.42 (s, 1H), 11.52 (s, 1H), 10.71 (s, 1H), 10.26 (s, 1H), 9.24 (s, 1H), 7.96 – 7.89 (m, 2H), 7.70 (d, *J* = 7.9 Hz, 1H), 7.57 (dt, *J* = 7.8, 1.3 Hz, 1H), 7.50 – 7.43 (m, 3H), 7.26 – 7.22 (m, 1H), 7.18 – 7.14 (m, 1H), 5.45 (s, 1H). ^13^C NMR (101 MHz, DMSO-*d*_6_) δ 162.9, 149.9, 148.8, 147.0, 146.8, 135.8, 134.0, 129.0, 124.8, 124.7, 121.8, 121.7, 120.7, 119.6, 119.2, 111.8, 103.8, 100.1, 86.1, 35.0.

***4-(3-Hydroxyphenyl)-3-(1H-indol-3-yl)-1,4,8,9-tetrahydro-5H-pyrazolo[4',3':5,6]pyrido[2,3-d]pyrimidine-5,7(6H)-dione (b9)***

Yellow solid; Mp: >300 ˚C; FT-IR (KBr, cm^-1^): 3428, 3371, 3224, 1712, 1697,1598. ^1^H NMR (400 MHz, DMSO-*d*_6_) δ 12.36, 11.53, 10.66, 10.12, 9.21, 9.05, 7.82, 7.80, 7.53, 7.51, 7.32, 7.31, 7.29, 7.27, 7.25, 7.21, 7.19, 7.17, 7.00, 6.98, 6.96, 6.65, 6.63, 6.54, 6.52, 5.17, 3.48, 2.65. ^13^C NMR (101 MHz, DMSO-*d*_6_) δ 162.8, 156.8, 149.9, 148.5, 146.6, 146.4, 135.8, 134.2, 128.4, 124.7, 124.4, 121.7, 119.6, 118.3, 114.3, 112.7, 111.7, 101.0, 87.4, 34.6.

***3-(1H-Indol-3-yl)-4-phenyl-1,4,8,9-tetrahydro-5H-pyrazolo[4',3':5,6]pyrido[2,3-d]pyrimidine-5,7(6H)-dione (b10)***

Yellow solid; Mp: >300 ˚C; FT-IR (KBr, cm^-1^): 3367, 3231, 1704, 1594, 1521, 1428. ^1^H NMR (400 MHz, DMSO-*d*_6_) δ 12.36 (s, 1H), 11.50 (s, 1H), 10.65 (d, *J* = 1.8 Hz, 1H), 10.15 (s, 1H), 9.07 (s, 1H), 7.78 (d, *J* = 7.9 Hz, 1H), 7.51 (d, *J* = 8.1 Hz, 1H), 7.33 (d, *J* = 2.7 Hz, 1H), 7.28 – 7.23 (m, 1H), 7.20 – 7.16 (m, 5H), 7.13 – 7.08 (m, 1H), 5.25 (s, 1H). ^13^C NMR (101 MHz, DMSO-*d*_6_) δ 162.9, 149.9, 147.1, 146.4, 135.8, 127.6, 127.3, 125.6, 124.7, 124.5, 121.7, 119.6, 119.5, 111.7, 104.4, 101.0, 87.3, 34.8.

***4-(2,3-Dihydroxyphenyl)-3-(1H-indol-3-yl)-1,4,8,9-tetrahydro-5H-pyrazolo[4',3':5,6]pyrido [2,3-d]pyrimidine-5,7(6H)-dione (b11)***

Yellow solid; Mp: >300 ˚C; FT-IR (KBr, cm^-1^): 3366, 3334, 3199, 1717, 1660, 1638. ^1^H NMR (400 MHz, DMSO-*d*_6_) δ 12.29 (s, 1H), 11.51 (s, 1H), 10.64 (s, 1H), 10.11 (s, 1H), 9.00 (s, 1H), 7.75 (d, *J* = 7.9 Hz, 1H), 7.50 (d, *J* = 7.8 Hz, 1H), 7.41 (d, *J* = 2.7 Hz, 1H), 7.26 – 7.16 (m, 2H), 6.73 (d, *J* = 8.3 Hz, 1H), 6.60 (s, 1H), 6.56 – 6.52 (m, 1H), 5.22 (s, 1H), 3.65 (s, 3H), 3.33 (s, 3H). ^13^C NMR (101 MHz, DMSO-*d*_6_) δ 163.0, 150.0, 147.5, 146.6, 146.5, 139.6, 135.9, 124.8, 124.7, 121.8, 119.7, 119.5, 118.3, 111.8, 111.4, 104.7, 101.4, 86.9, 55.3, 54.6, 34.2.

***3-(1H-Indol-3-yl)-4-(2-methoxyphenyl)-1,4,8,9-tetrahydro-5H-pyrazolo[4',3':5,6]pyrido[2,3-d]pyrimidine-5,7(6H)-dione (b12)***

Yellow solid; Mp: >300 ˚C; FT-IR (KBr, cm^-1^): 3255, 1711, 1636, 1610, 1518, 1435. ^1^H NMR (400 MHz, DMSO-*d*_6_) δ 12.15 (s, 1H), 11.48 (s, 1H), 10.51 (s, 1H), 10.06 (s, 1H), 8.96 (s, 1H), 7.70 (d, *J* = 8.1 Hz, 1H), 7.49 (d, *J* = 8.1 Hz, 1H), 7.38 (s, 1H), 7.25 – 7.20 (m, 1H), 7.16 – 7.03 (m, 3H), 6.83 – 6.77 (m, 2H), 5.52 (s, 1H), 3.47 (s, 3H). ^13^C NMR (101 MHz, DMSO-*d*_6_) δ 162.6, 156.0, 150.0, 147.0, 146.5, 135.7, 135.6, 134.0, 129.3, 126.7, 125.1, 124.8, 121.5, 119.9, 119.5, 119.3, 111.5, 111.2, 104.3, 101.6, 87.1, 55.0, 29.2.

***4-(2,3-Dihydroxyphenyl)-3-(1H-indol-3-yl)-1,4,8,9-tetrahydro-5H-pyrazolo[4',3':5,6]pyrido[2,3-d]pyrimidine-5,7(6H)-dione (b13)***

Yellow solid; Mp: >300 ˚C; FT-IR (KBr, cm^-1^): 3561, 3347, 3196, 1696, 1631, 1544. ^1^H NMR (400 MHz, DMSO-*d*_6_) δ 12.39 (s, 1H), 11.54 (s, 1H), 11.18 (s, 1H), 10.52 (s, 1H), 10.19 (s, 1H), 9.41 (s, 1H), 8.69 (s, 1H), 7.90 (d, *J* = 7.9 Hz, 1H), 7.43 (d, *J* = 8.1 Hz, 1H), 7.24 – 7.20 (m, 1H), 7.17 – 7.13 (m, 1H), 6.80 (d, *J* = 2.8 Hz, 1H), 6.64 – 6.61 (m, 2H), 6.30 – 6.26 (m, 1H), 5.37 (s, 1H). ^13^C NMR (101 MHz, DMSO-*d*_6_) δ 166.1, 149.1, 147.9, 147.2, 146.6, 141.2, 135.7, 135.5, 134.5, 124.3, 123.7, 121.9, 120.5, 119.6, 119.6, 118.8, 113.2, 111.6, 104.5, 100.1, 87.1, 28.2.

***4,4'-(1,4-Phenylene)bis(3-(1H-indol-3-yl)-1,4,8,9-tetrahydro-5H-pyrazolo[4',3':5,6]pyrido[2,3-d]pyrimidine-5,7(6H)-dione) (b14)***

Yellow solid; Mp: >300 ˚C; FT-IR (KBr, cm^-1^): 3270, 1709, 1628, 1606, 1572, 1510. ^1^H NMR (400 MHz, DMSO-*d*_6_) δ 12.32 (s, 2H), 11.47 (s, 2H), 10.60 (s, 2H), 10.07 (s, 2H), 8.96 (s, 2H), 7.82 – 7.78 (m, 2H), 7.50 (d, *J* = 7.9 Hz, 2H), 7.27 – 7.26 (m, 2H), 7.17 – 7.16 (m, 2H), 6.98 – 6.96 (m, 3H), 6.58 (d, *J* = 8.6 Hz, 3H), 5.11 (s, 2H). ^13^C NMR (101 MHz, DMSO-*d*_6_) δ 162.9, 155.1, 149.9, 146.2, 137.7, 135.8, 128.3, 124.7, 124.6, 124.4, 121.7, 120.7, 119.6, 114.3, 111.7, 104.5, 101.3, 87.8, 33.8.

***4,4',4''-(((1,3,5-Triazine-2,4,6-triyl)tris(oxy))tris(benzene-4,1-diyl))tris(3-(1H-indol-3-yl)-1,4,8,9-tetrahydro-5H-pyrazolo[4',3':5,6]pyrido[2,3-d]pyrimidine-5,7(6H)-dione) (b15)***

Yellow solid; Mp: >300 ˚C; FT-IR (KBr, cm^-1^): 3249, 1712, 1630, 1608, 1571,1513. ^1^H NMR (400 MHz, DMSO-*d*_6_) δ 12.30 (s, 3H), 11.32 (s, 3H), 10.52 (d, *J* = 1.7 Hz, 3H), 10.03 (s, 3H), 8.92 (s, 3H), 7.71 – 7.67 (m, 5H), 7.53 – 7.50 (m, 3H), 7.46 – 7.44 (m, 3H), 7.25 – 7.21 (m, 5H), 7.05 – 7.04 (m, 3H), 6.94 – 6.92 (m, 8H), 5.05 (s, 3H). ^13^C NMR (101 MHz, DMSO-*d*_6_) δ 162.8, 162.8, 149.8, 146.6, 144.5, 144.4, 135.7, 126.8, 126.7, 124.7, 124.7, 124.2, 121.7, 119.6, 111.7, 104.4, 101.2, 101.1, 87.3, 87.3, 34.2.

***4-(4-Chlorophenyl)-3-(1H-indol-3-yl)-6,8-dimethyl-1,4,8,9-tetrahydro-5H-pyrazolo[4',3':5,6]pyrido[2,3-d]pyrimidine-5,7(6H)-dione (c1)***

White solid; Mp: >300 ˚C; FT-IR (KBr, cm^-1^): 3281, 1706, 1611, 1586, 1570, 1486. ^1^H NMR (400 MHz, DMSO-*d*_6_) δ 12.48 (s, 1H), 11.52 (s, 1H), 10.15 (s, 1H), 7.78 (d, *J* = 7.9 Hz, 1H), 7.53 (d, *J* = 7.9 Hz, 1H), 7.39 (d, *J* = 2.7 Hz, 1H), 7.29 – 7.25 (m, 1H), 7.23 – 7.19 (m, 3H), 7.15 (d, *J* = 8.6 Hz, 2H), 5.39 (s, 1H), 3.61 (s, 3H), 3.20 (s, 3H). ^13^C NMR (101 MHz, DMSO-*d*_6_) δ 160.8, 150.7, 147.0, 146.6, 146.1, 135.8, 130.0, 129.1, 127.5, 124.7, 124.5, 121.8, 119.6, 119.4, 111.8, 100.8, 87.3, 35.3, 30.1, 27.5.

***3-(1H-Indol-3-yl)-6,8-dimethyl-4-(4-nitrophenyl)-1,4,8,9-tetrahydro-5H-pyrazolo[4',3':5,6]pyrido[2,3-d]pyrimidine-5,7(6H)-dione (c2)***

Brown solid; Mp: >300 ˚C; FT-IR (KBr, cm^-1^): 3409, 3314, 3060, 1710, 1695, 1635. ^1^H NMR (400 MHz, DMSO-*d*_6_) δ 12.52 (s, 1H), 11.51 (s, 1H), 10.22 (s, 1H), 8.00 (d, *J* = 8.9 Hz, 2H), 7.71 (d, *J* = 7.9 Hz, 1H), 7.51 (d, *J* = 8.1 Hz, 1H), 7.44 (d, *J* = 2.7 Hz, 1H), 7.35 (d, *J* = 8.7 Hz, 2H), 7.25 (t, *J* = 7.0 Hz, 1H), 7.17 (t, *J* = 7.5 Hz, 1H), 5.53 (s, 1H), 3.61 (s, 3H), 3.18 (s, 3H). ^13^C NMR (101 MHz, DMSO-*d*_6_) δ 160.8, 154.6, 150.7, 146.9, 146.8, 145.4, 135.8, 134.4, 128.5, 124.8, 122.8, 121.8, 119.6, 119.3, 111.9, 103.8, 100.2, 86.5, 36.1, 30.2, 27.5.

***3-(1H-Indol-3-yl)-6,8-dimethyl-4-(m-tolyl)-1,4,8,9-tetrahydro-5H-pyrazolo[4',3':5,6]pyrido[2,3-d]pyrimidine-5,7(6H)-dione* (c3)**

Yellow solid; Mp: >300 ˚C; FT-IR (KBr, cm^-1^): 3405, 3235, 1693, 1612, 1512, 1488. ^1^H NMR (400 MHz, DMSO-*d*_6_) δ 12.25 (s, 1H), 11.48 (s, 1H), 10.05 (s, 1H), 7.82 (d, *J* = 7.9 Hz, 1H), 7.51 (dd, *J* = 8.1, 0.9 Hz, 1H), 7.32 (d, *J* = 2.6 Hz, 1H), 7.28 – 7.23 (m, 1H), 7.20 – 7.15 (m, 1H), 7.10 – 7.07 (m, 2H), 6.76 – 6.72 (m, 2H), 5.31 (s, 1H), 3.71 (s, 3H), 3.59 (s, 3H), 3.20 (s, 3H). ^13^C NMR (101 MHz, DMSO-*d*_6_) δ 160.8, 150.7, 147.2, 146.6, 136.4, 135.8, 128.0, 127.5, 126.3, 124.8, 124.5, 121.7, 119.6, 119.5, 111.8, 104.4, 101.5, 87.8, 35.6, 30.1, 27.5, 21.0.

***3-(1H-Indol-3-yl)-4-(4-methoxyphenyl)-6,8-dimethyl-1,4,8,9-tetrahydro-5H-pyrazolo[4',3':5,6]pyrido[2,3-d]pyrimidine-5,7(6H)-dione (c4)***

Yellow solid; Mp: >300 ˚C; FT-IR (KBr, cm^-1^): 3388, 3253, 1702, 1623, 1574, 1515. ^1^H NMR (400 MHz, DMSO-*d*_6_) δ 12.37 (s, 1H), 11.49 (s, 1H), 10.04 (s, 1H), 7.77 (d, *J* = 7.8 Hz, 1H), 7.52 (d, *J* = 7.1 Hz, 1H), 7.32 (d, *J* = 2.7 Hz, 1H), 7.26 (t, *J* = 6.9 Hz, 1H), 7.18 (t, *J* = 7.5 Hz, 1H), 7.05 (t, *J* = 7.5 Hz, 1H), 6.96 (d, *J* = 7.8 Hz, 1H), 6.89 (d, *J* = 10.6 Hz, 2H), 5.31 (s, 1H), 3.60 (s, 3H), 3.20 (s, 3H), 2.17 (s, 3H). ^13^C NMR (101 MHz, DMSO-*d*_6_) δ 160.8, 157.1, 150.7, 147.1, 146.3, 139.5, 135.8, 133.9, 128.3, 124.7, 124.4, 121.8, 119.6, 113.0, 111.8, 104.5, 101.4, 88.2, 54.8, 34.8, 30.0, 27.5.

***3-(1H-Indol-3-yl)-4-(2-methoxyphenyl)-6,8-dimethyl-1,4,8,9-tetrahydro-5H-pyrazolo[4',3':5,6]pyrido[2,3-d]pyrimidine-5,7(6H)-dione (c5)***

Yellow solid; Mp: >300 ˚C; FT-IR (KBr, cm^-1^): 3273, 3070, 1679, 1626, 1569, 1514. ^1^H NMR (400 MHz, DMSO-*d*_6_) δ 12.19 (s, 1H), 11.46 (s, 1H), 9.97 (s, 1H), 7.72 (d, *J* = 7.8 Hz, 1H), 7.50 (d, *J* = 8.1 Hz, 1H), 7.38 (d, *J* = 2.7 Hz, 1H), 7.26 – 7.21 (m, 1H), 7.16 – 7.10 (m, 2H), 7.07 – 7.03 (m, 1H), 6.80 – 6.75 (m, 2H), 5.64 (s, 1H), 3.62 (s, 3H), 3.46 (s, 3H), 3.16 (s, 3H). ^13^C NMR (101 MHz, DMSO-*d*_6_) δ 160.5, 156.0, 150.8, 135.8, 129.3, 126.7, 125.2, 124.7, 121.5, 119.9, 119.5, 119.3, 111.5, 111.0, 104.3, 101.8, 87.7, 54.8, 30.0, 28.0, 27.4.

***3-(1H-Indol-3-yl)-6,8-dimethyl-4-(3-nitrophenyl)-1,4,8,9-tetrahydro-5H-pyrazolo[4',3':5,6]pyrido[2,3-d]pyrimidine-5,7(6H)-dione (c6)***

Brown solid; Mp: >300 ˚C; FT-IR (KBr, cm^-1^): 3453, 3359, 3219, 1692, 1618, 1570. ^1^H NMR (400 MHz, DMSO-*d*_6_) δ 12.48 (s, 1H), 11.50 (s, 1H), 10.22 (s, 1H), 7.93 – 7.89 (m, 1H), 7.88 – 7.85 (m, 1H), 7.69 (d, *J* = 7.9 Hz, 1H), 7.58 (d, *J* = 7.8 Hz, 1H), 7.50 (d, *J* = 8.1 Hz, 1H), 7.46 – 7.41 (m, 2H), 7.26 – 7.22 (m, 1H), 7.18 – 7.13 (m, 1H), 5.54 (s, 1H), 3.61 (s, 3H), 3.18 (s, 3H).

***3-(1H-Indol-3-yl)-6,8-dimethyl-4-(pyridin-3-yl)-1,4,8,9-tetrahydro-5H-pyrazolo[4',3':5,6]pyrido[2,3-d]pyrimidine-5,7(6H)-dione (c7)***

Yellow solid; Mp: >300 ˚C; FT-IR (KBr, cm^-1^): 3290, 3062, 1713, 1631, 1568, 1509. ^1^H NMR (400 MHz, DMSO-*d*_6_) δ 12.49 (s, 1H), 11.52 (s, 1H), 10.18 (s, 1H), 8.29 (d, *J* = 6.2 Hz, 2H), 7.74 (d, *J* = 7.9 Hz, 1H), 7.53 (d, *J* = 7.1 Hz, 1H), 7.45 (d, *J* = 2.7 Hz, 1H), 7.29 – 7.24 (m, 1H), 7.21 – 7.16 (m, 1H), 7.06 (d, *J* = 4.6 Hz, 2H), 5.40 (s, 1H), 3.61 (s, 3H), 3.19 (s, 3H). ^13^C NMR (101 MHz, DMSO-*d*_6_) δ 172.0, 160.8, 157.1, 150.7, 146.3, 139.5, 135.8, 128.3, 124.7, 124.4, 121.88, 119.6, 113.0, 111.8, 104.5, 101.4, 88.2, 54.8, 30.0, 27.5, 21.0.


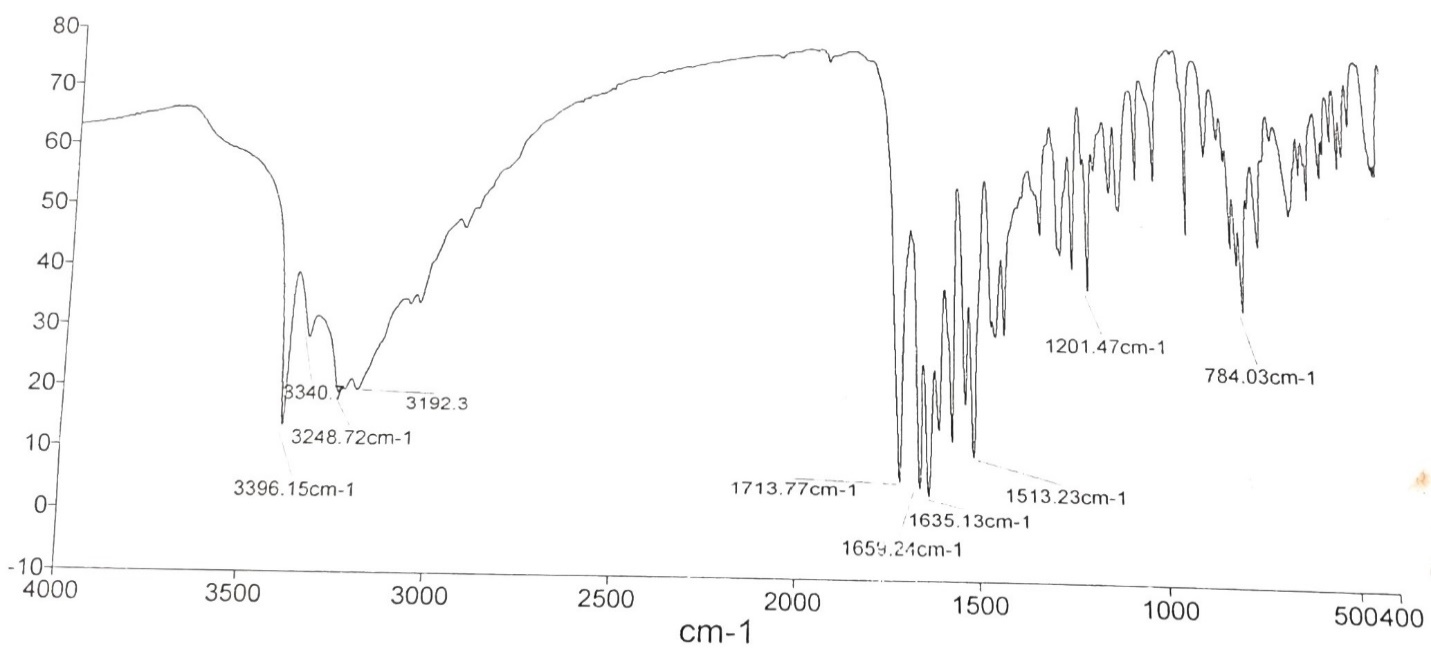


*FT-IR spectrum of 3-(1H-indol-3-yl)-4-(p-tolyl)-1,4,8,9-tetrahydro-5H pyrazolo[4',3':5,6]pyrido[2,3-d]pyrimidine-5,7(6H)-dione (b1).*


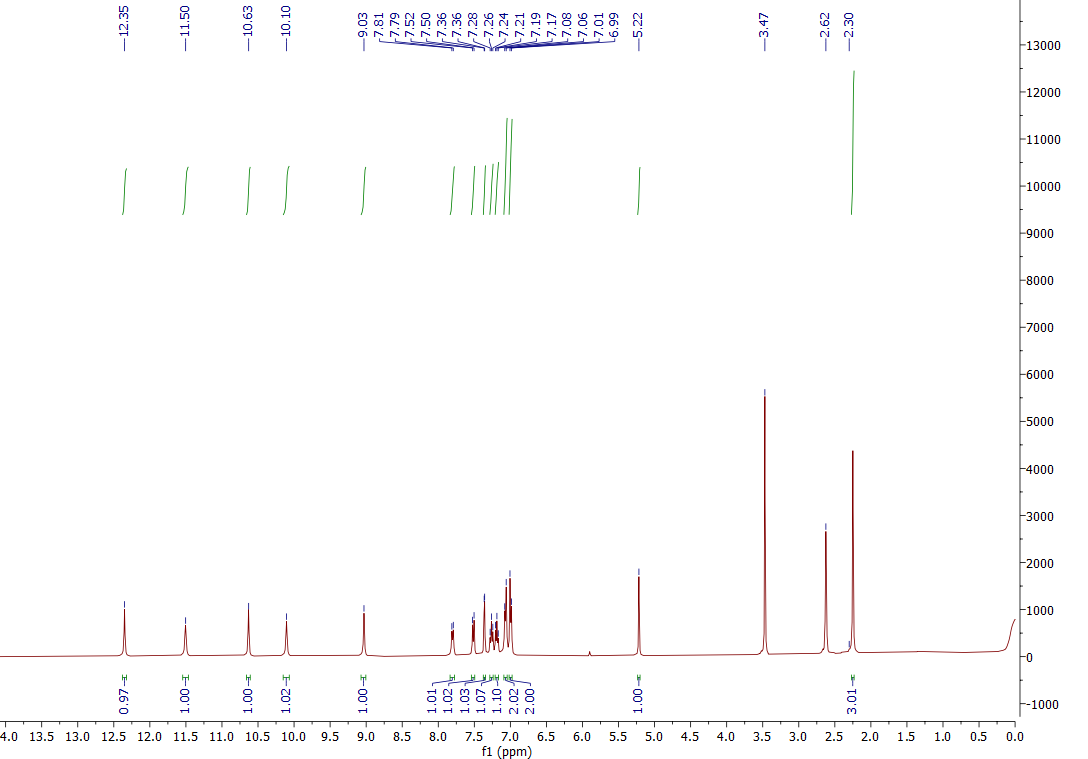


*^1^H-NMR spectrum of 3-(1H-indol-3-yl)-4-(p-tolyl)-1,4,8,9-tetrahydro-5H pyrazolo[4',3':5,6]pyrido[2,3-d]pyrimidine-5,7(6H)-dione (b1).*

*
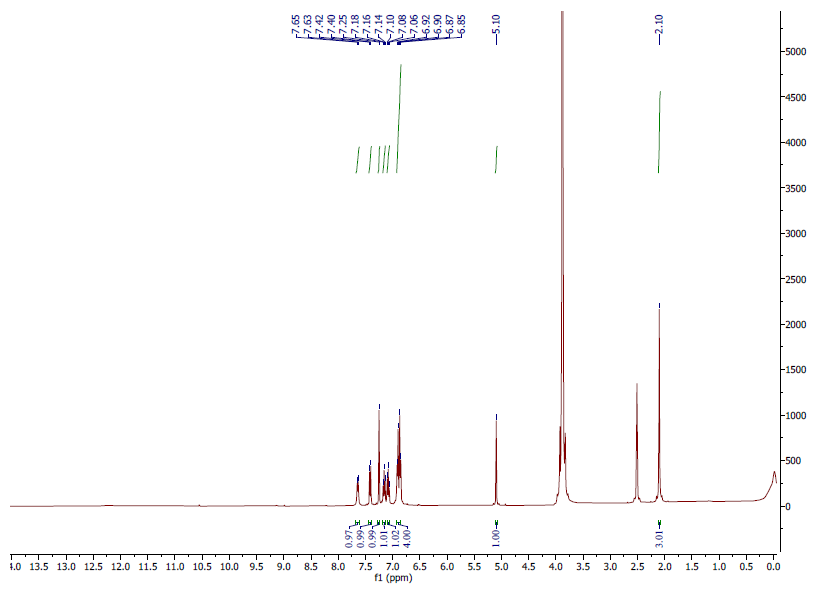
*

*^1^H-NMR spectrum of3-(1H-indol-3-yl)-4-(p-tolyl)-1,4,8,9-tetrahydro-5H pyrazolo[4',3':5,6]pyrido[2,3-d]pyrimidine-5,7(6H)-dione (b1). in DMSO-d_6_ and D_2_O as solvent.*


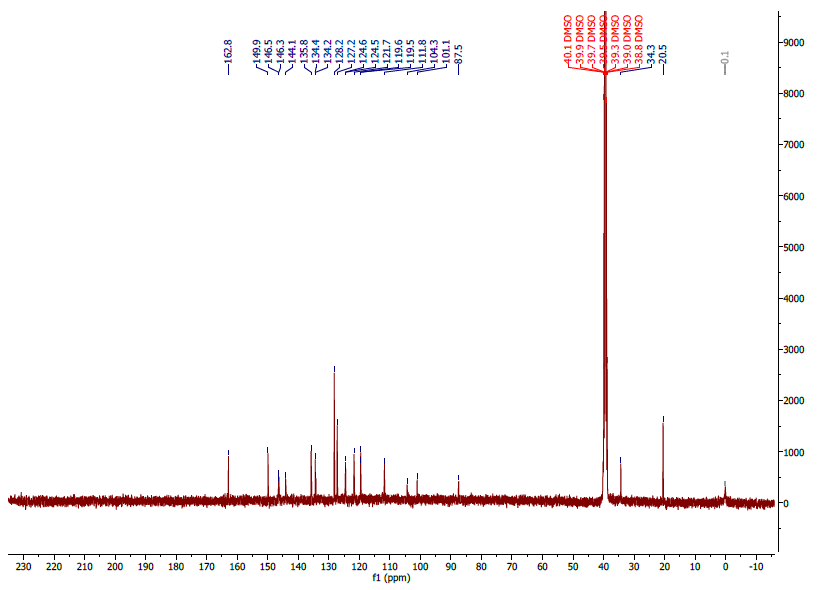


*^13^C-NMR spectrum of3-(1H-indol-3-yl)-4-(p-tolyl)-1,4,8,9-tetrahydro-5H pyrazolo[4',3':5,6]pyrido[2,3-d]pyrimidine-5,7(6H)-dione (b1).*


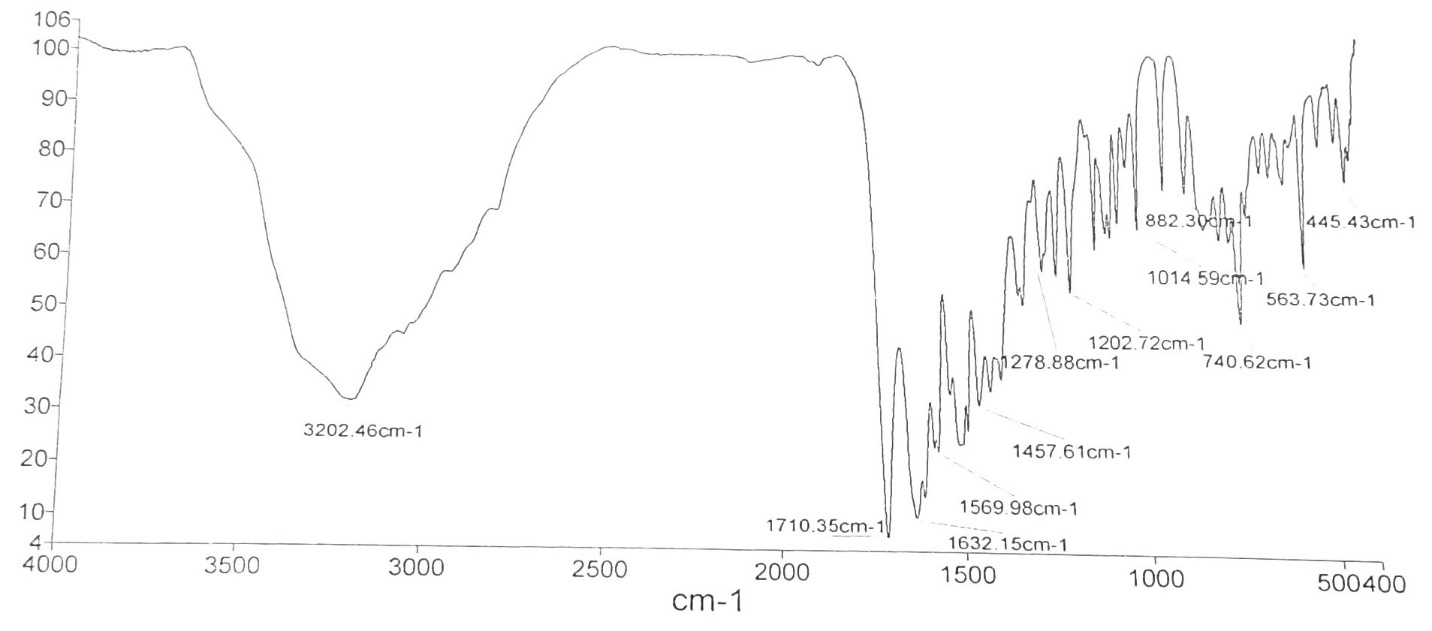


*FT-IR spectrum of 4-(4-chlorophenyl)-3-(1H-indol-3-yl)-1,4,8,9-tetrahydro-5H-pyrazolo[4',3':5,6]pyrido[2,3-d]pyrimidine-5,7(6H)-dione (b2).*


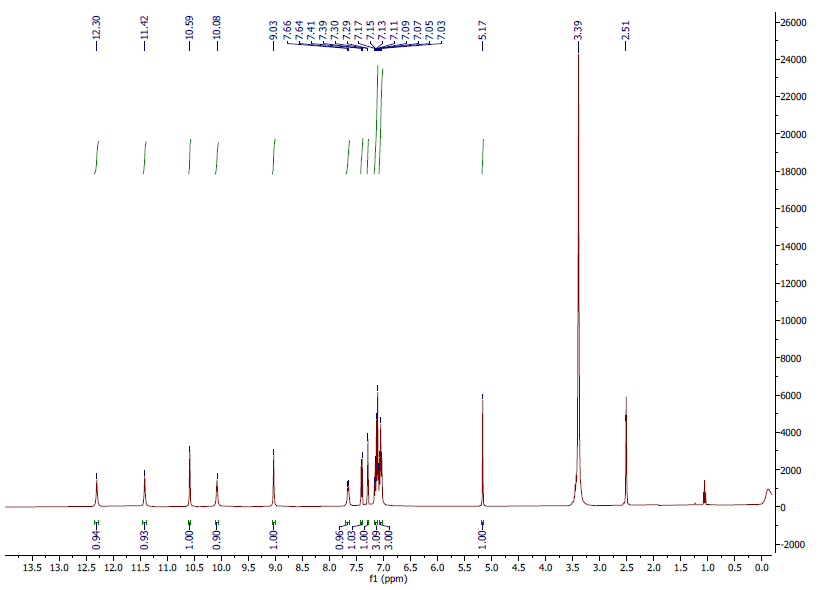


*^1^H-NMR spectrum of4-(4-chlorophenyl)-3-(1H-indol-3-yl)-1,4,8,9-tetrahydro-5H-pyrazolo[4',3':5,6]pyrido[2,3-d]pyrimidine-5,7(6H)-dione (b2).*


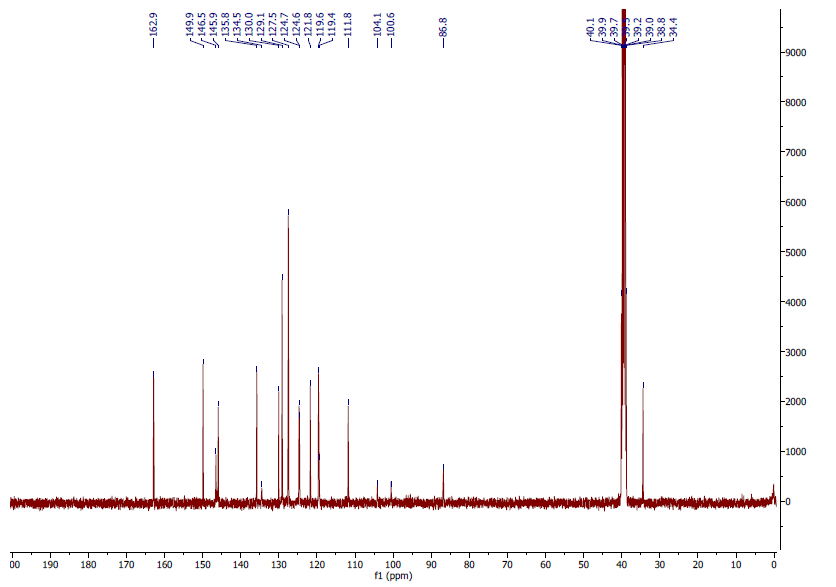


*^13^C-NMR spectrum of4-(4-chlorophenyl)-3-(1H-indol-3-yl)-1,4,8,9-tetrahydro-5H-pyrazolo[4',3':5,6]pyrido[2,3-d]pyrimidine-5,7(6H)-dione (b2).*


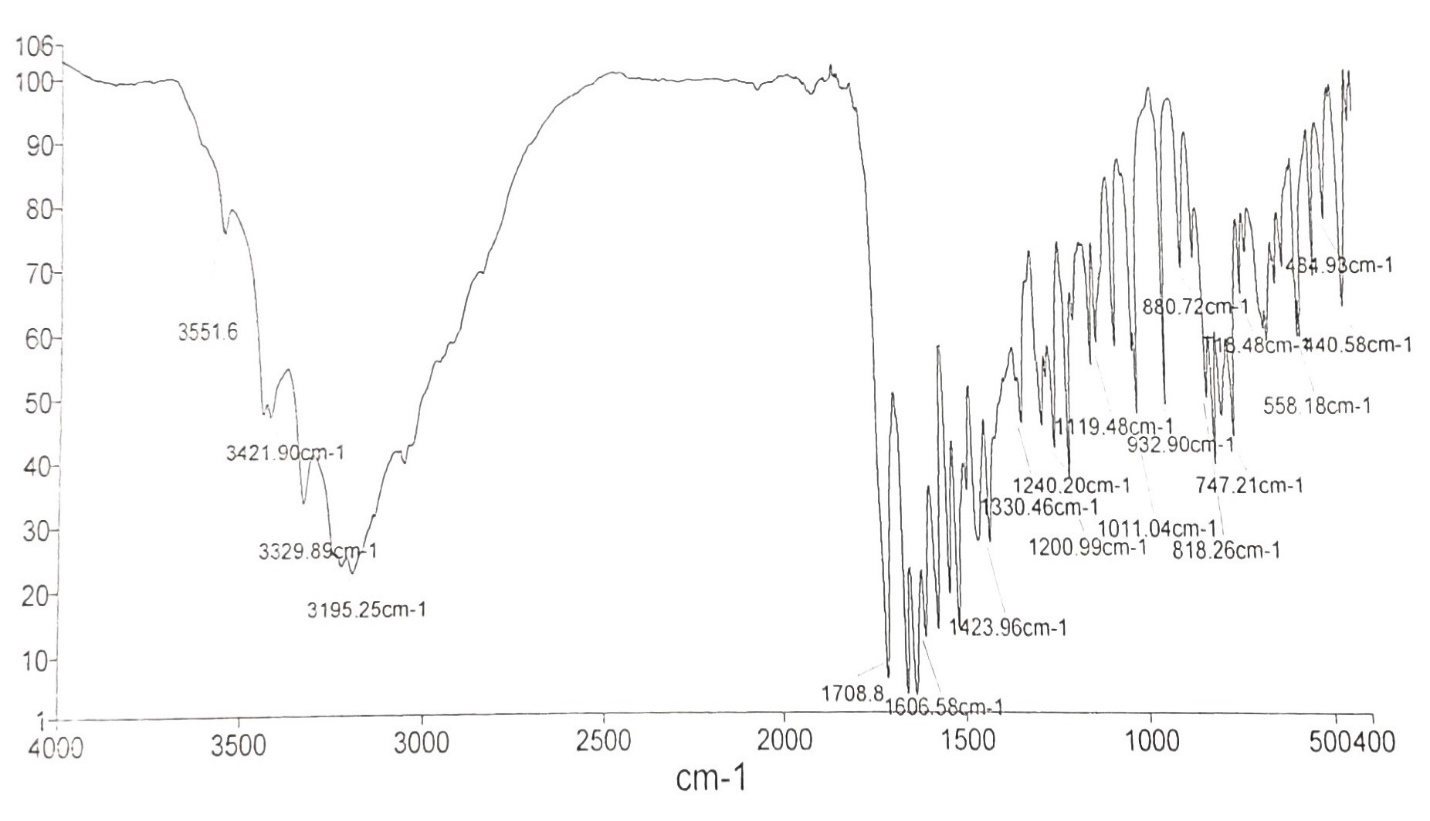


*FT-IR spectrum of 4-(4-bromophenyl)-3-(1H-indol-3-yl)-1,4,8,9-tetrahydro-5H-pyrazolo[4',3':5,6]pyrido[2,3-d]pyrimidine-5,7(6H)-dione (b3).*


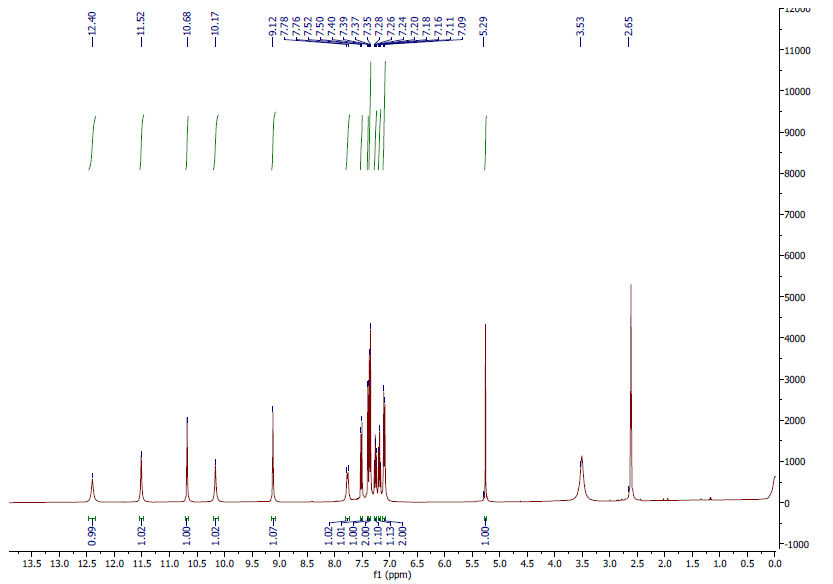


*^1^H-NMR spectrum of 4-(4-bromophenyl)-3-(1H-indol-3-yl)-1,4,8,9-tetrahydro-5H-pyrazolo[4',3':5,6]pyrido[2,3-d]pyrimidine-5,7(6H)-dione (b3).*


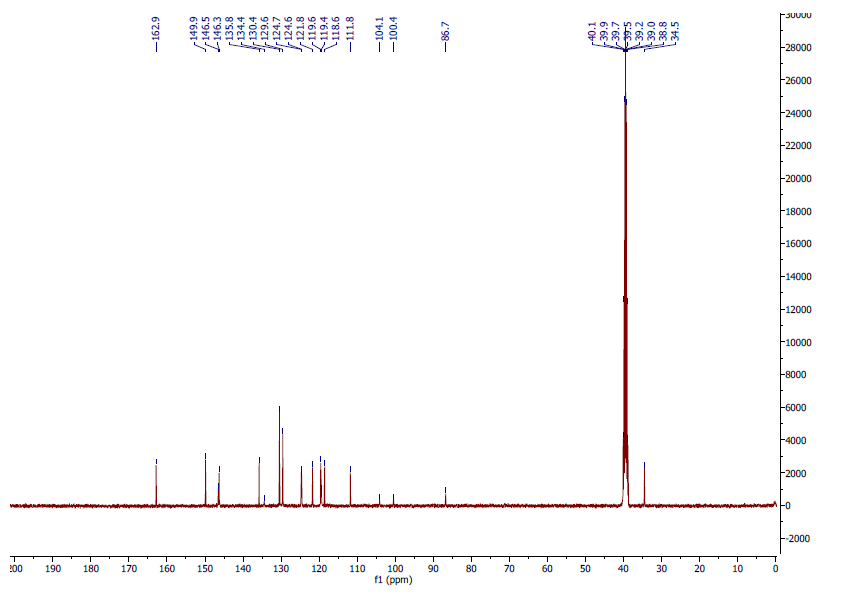


*^13^C-NMR spectrum of4-(4-bromophenyl)-3-(1H-indol-3-yl)-1,4,8,9-tetrahydro-5H-pyrazolo[4',3':5,6]pyrido[2,3-d]pyrimidine-5,7(6H)-dione (b3).*


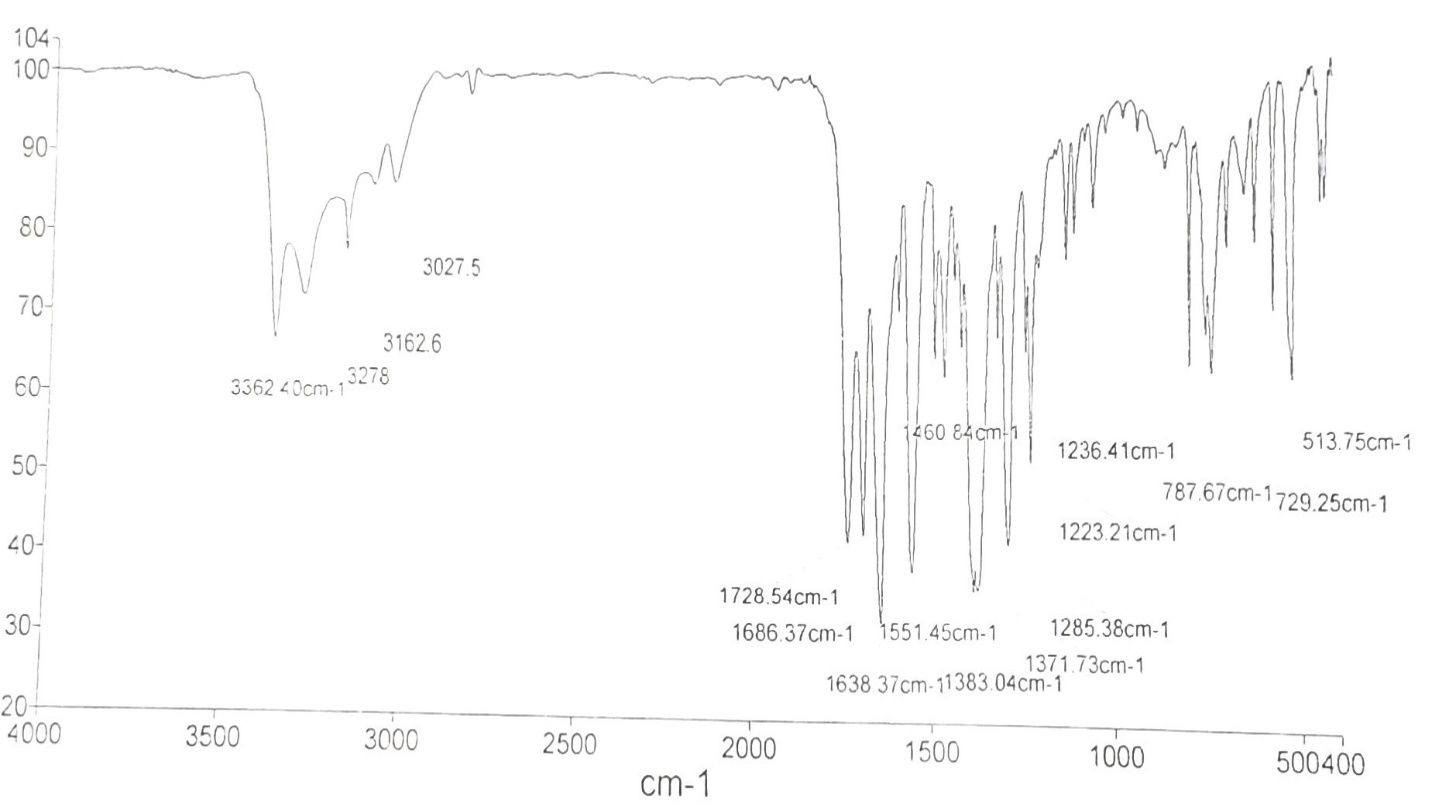


*FT-IR spectrum of 3-(1H-indol-3-yl)-4-(4-methoxyphenyl)-1,4,8,9-tetrahydro-5H-pyrazolo[4',3':5,6]pyrido[2,3-d]pyrimidine-5,7(6H)-dione (b4).*


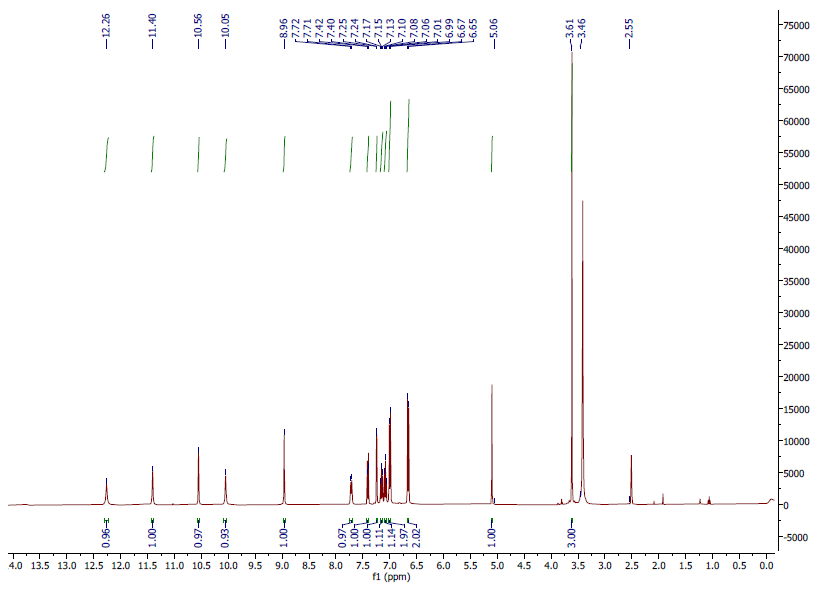


*^1^H-NMR spectrum of 3-(1H-indol-3-yl)-4-(4-methoxyphenyl)-1,4,8,9-tetrahydro-5H-pyrazolo[4',3':5,6]pyrido[2,3-d]pyrimidine-5,7(6H)-dione (b4).*


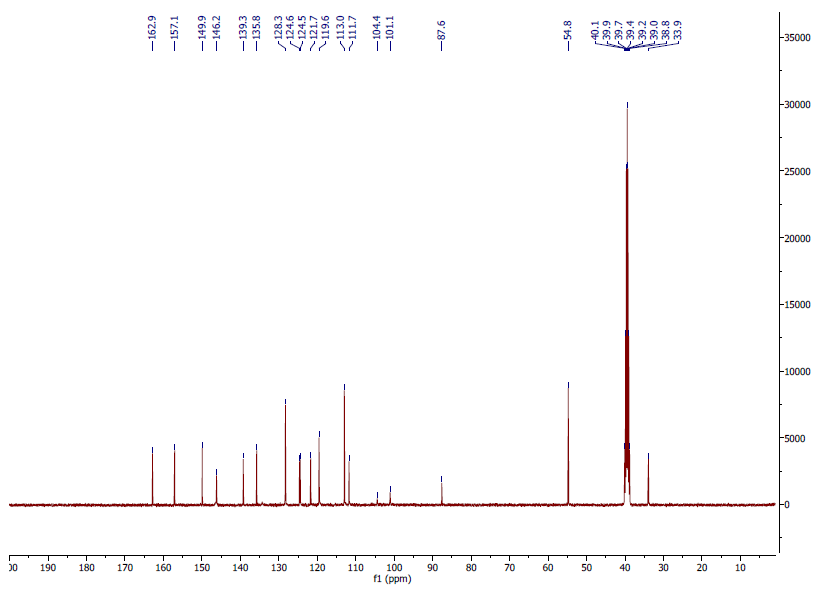


*^13^C-NMR spectrum of3-(1H-indol-3-yl)-4-(4-methoxyphenyl)-1,4,8,9-tetrahydro-5H-pyrazolo[4',3':5,6]pyrido[2,3-d]pyrimidine-5,7(6H)-dione (b4).*


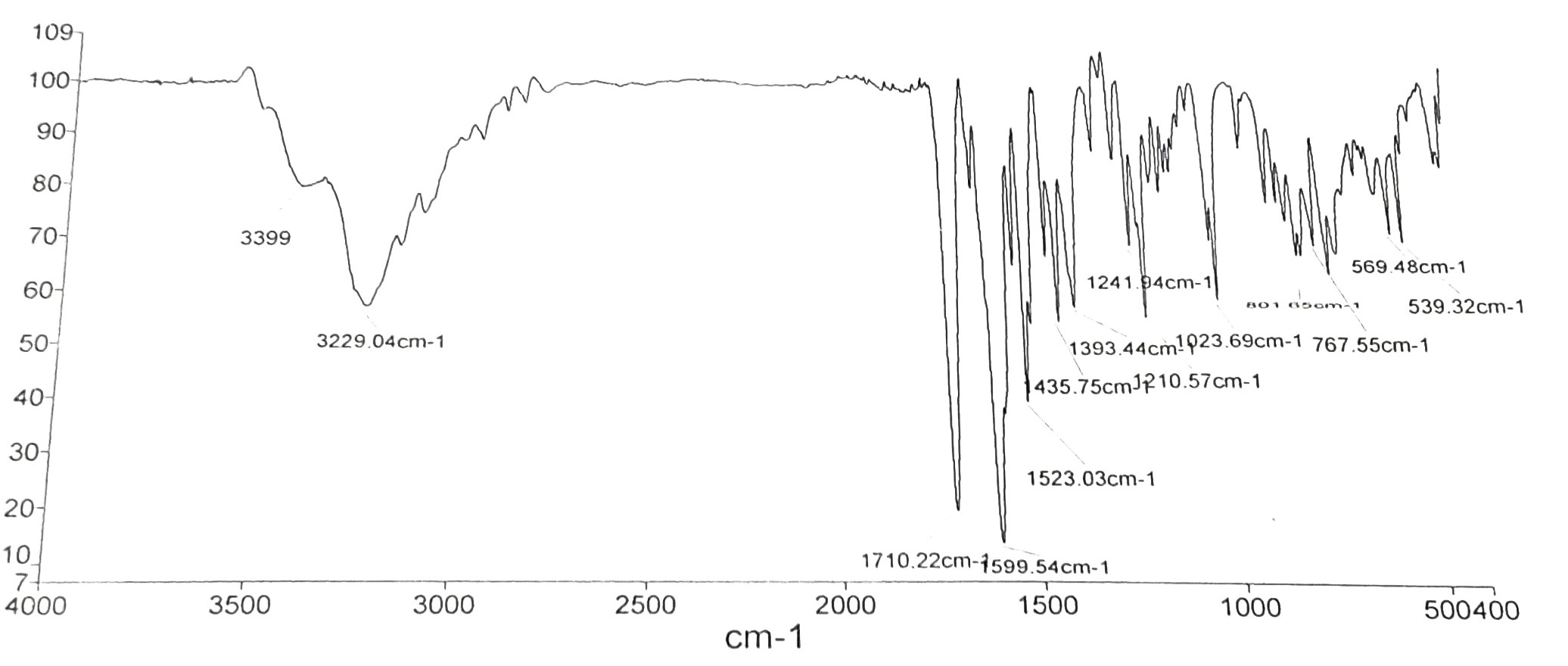
*FT-IR spectrum of 4-(4-fluorophenyl)-3-(1H-indol-3-yl)-1,4,8,9-tetrahydro-5H-pyrazolo[4',3':5,6]pyrido[2,3-d]pyrimidine-5,7(6H)-dione (b5).*


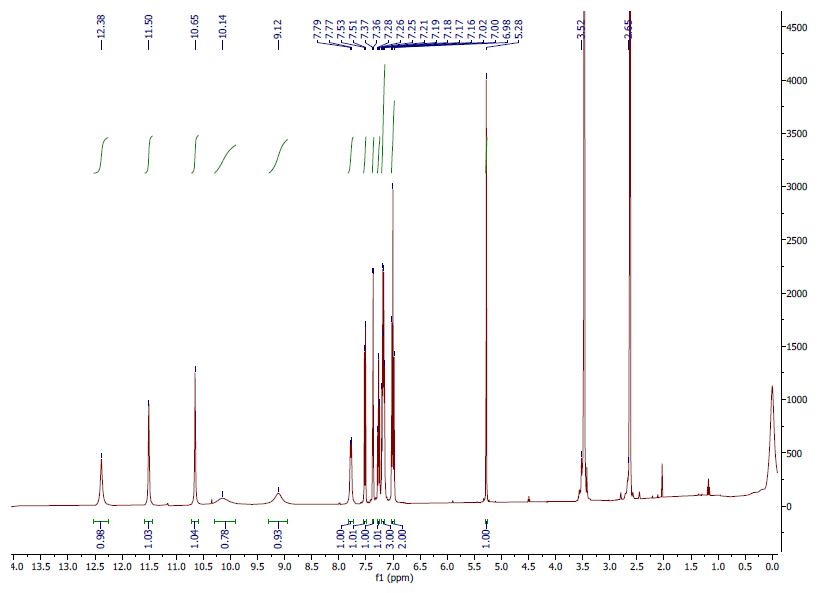


*^1^H-NMR spectrum of 4-(4-fluorophenyl)-3-(1H-indol-3-yl)-1,4,8,9-tetrahydro-5H-pyrazolo[4',3':5,6]pyrido[2,3-d]pyrimidine-5,7(6H)-dione (b5).*


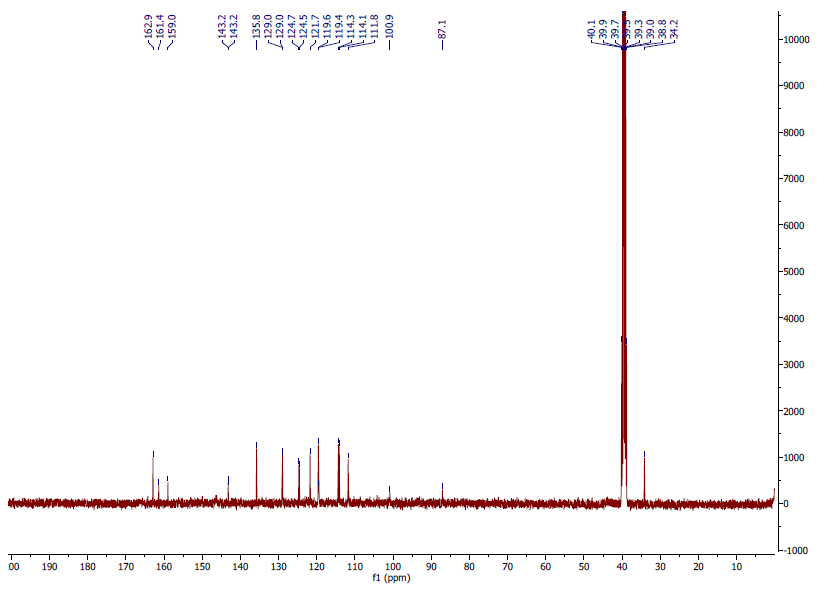


*^13^C-NMR spectrum of4-(4-fluorophenyl)-3-(1H-indol-3-yl)-1,4,8,9-tetrahydro-5H-pyrazolo[4',3':5,6]pyrido[2,3-d]pyrimidine-5,7(6H)-dione (b5).*


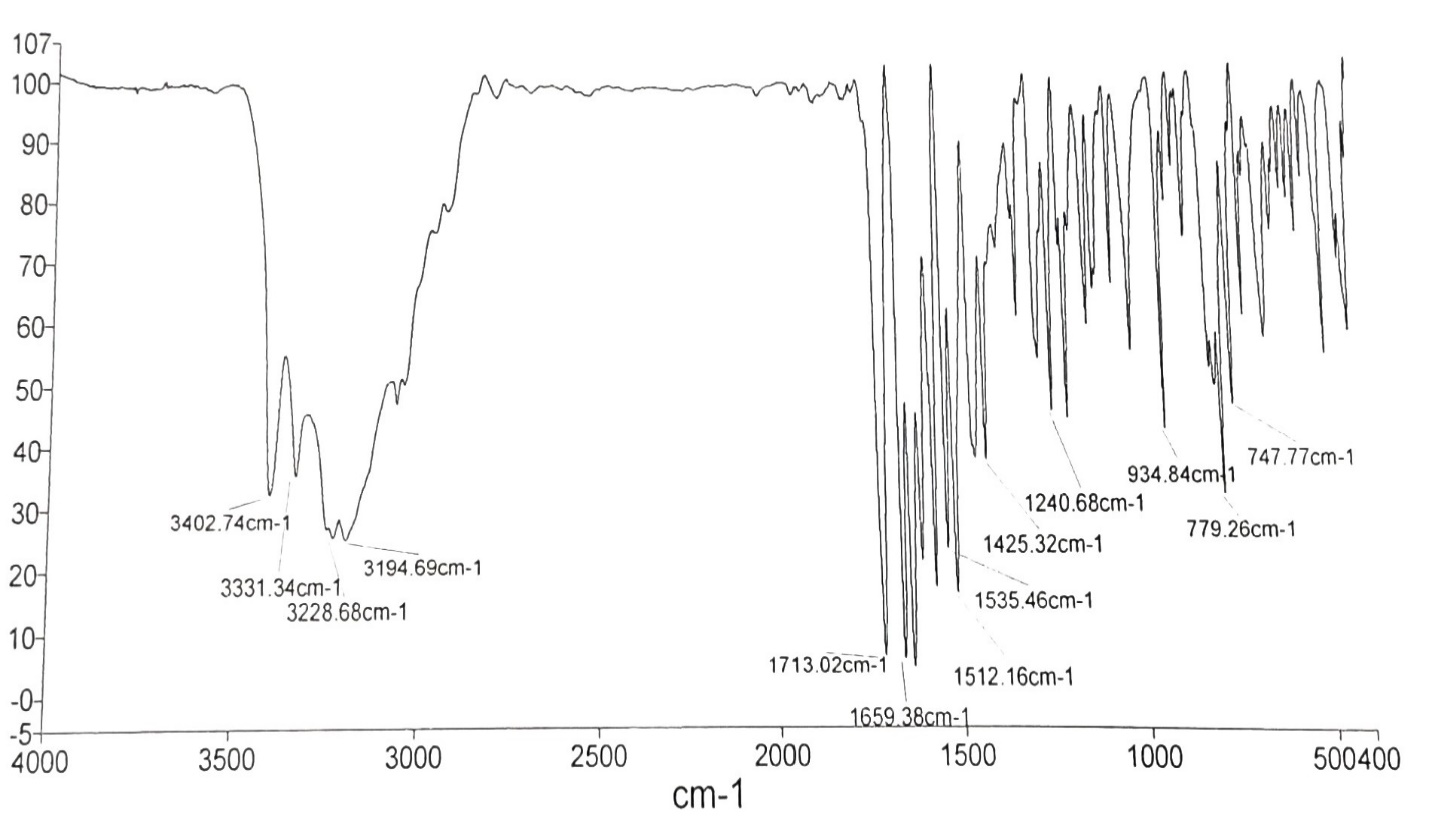


*FT-IR spectrum of 3-(1H-indol-3-yl)-4-(m-tolyl)-1,4,8,9-tetrahydro-5H-pyrazolo[4',3':5,6]pyrido[2,3-d]pyrimidine-5,7(6H)-dione (b6).*


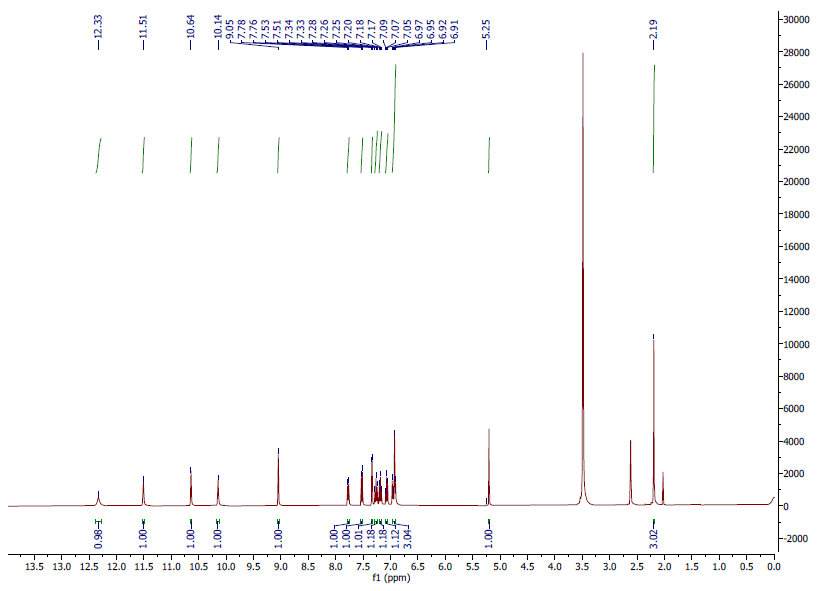


*^1^H-NMR spectrum of 3-(1H-indol-3-yl)-4-(m-tolyl)-1,4,8,9-tetrahydro-5H-pyrazolo[4',3':5,6]pyrido[2,3-d]pyrimidine-5,7(6H)-dione (b6).*


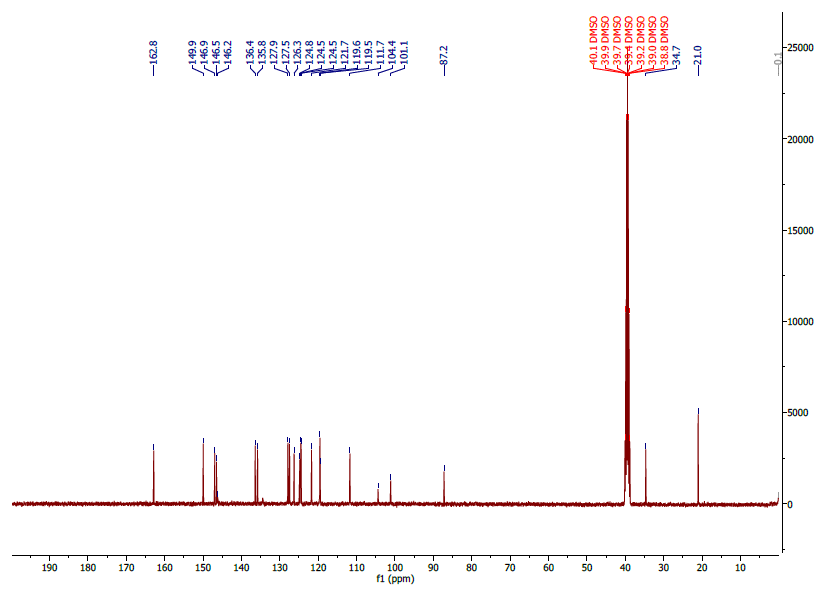


*^13^C-NMR spectrum of3-(1H-indol-3-yl)-4-(m-tolyl)-1,4,8,9-tetrahydro-5H-pyrazolo[4',3':5,6]pyrido[2,3-d]pyrimidine-5,7(6H)-dione (b6).*


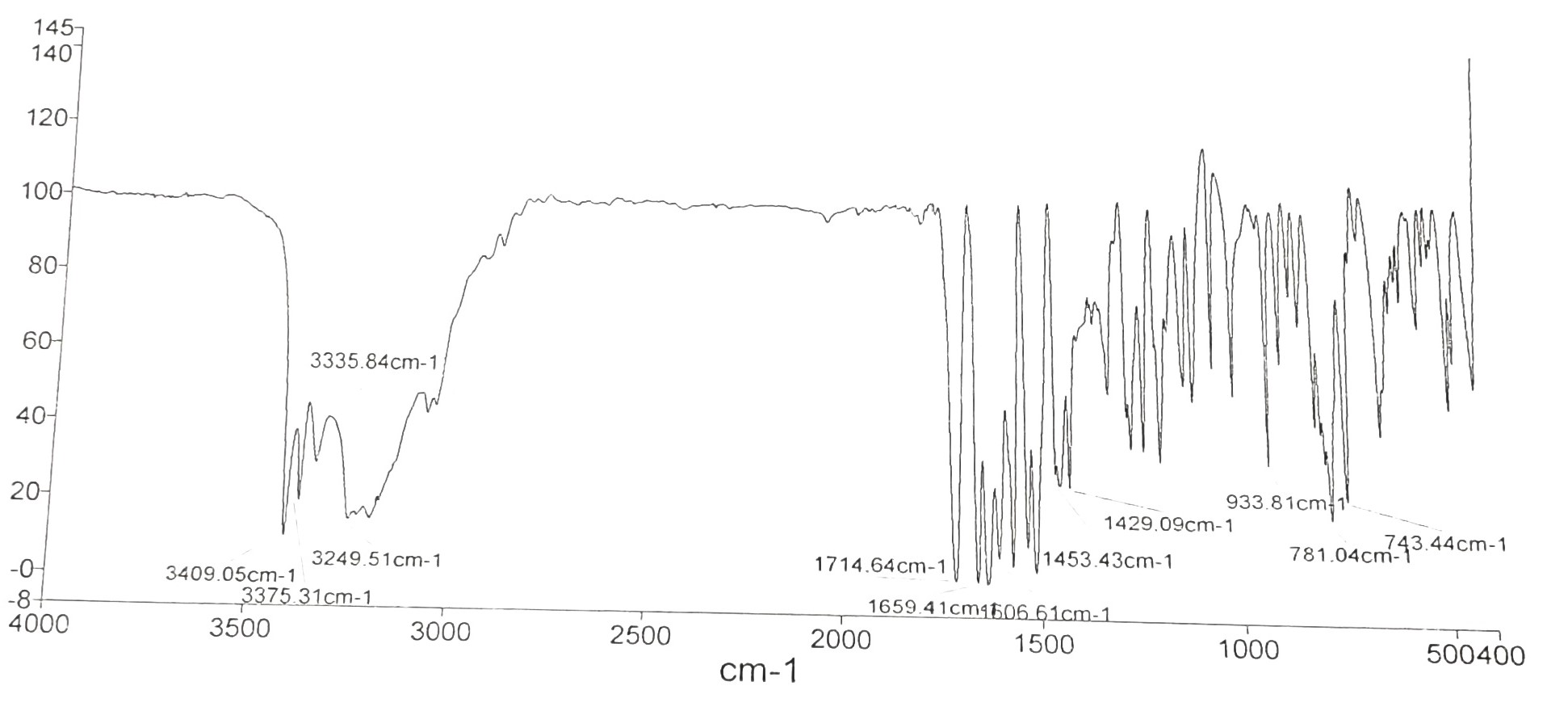


*FT-IR spectrum of 3-(1H-indol-3-yl)-4-(naphthalen-2-yl)-1,4,8,9-tetrahydro-5H-pyrazolo[4',3':5,6]pyrido[2,3-d]pyrimidine-5,7(6H)-dione (b7).*


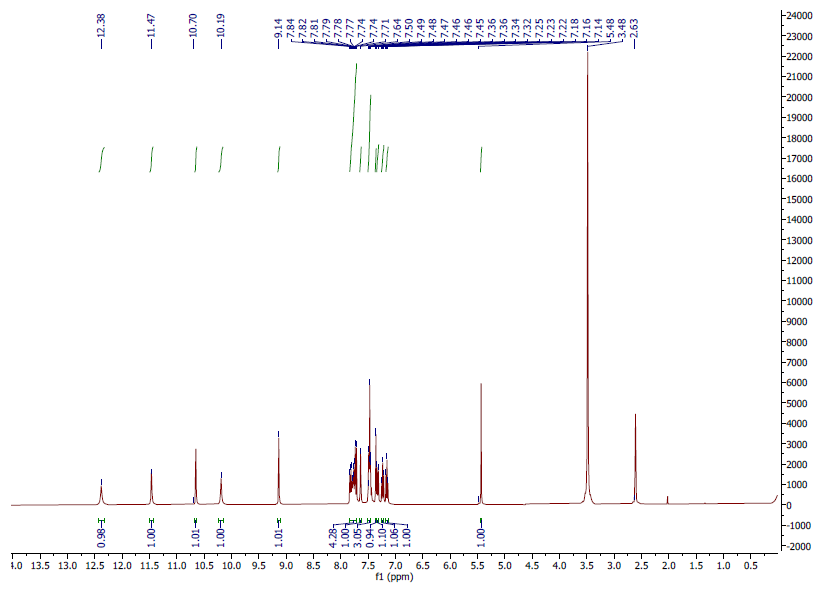


*^1^H-NMR spectrum of 3-(1H-indol-3-yl)-4-(naphthalen-2-yl)-1,4,8,9-tetrahydro-5H-pyrazolo[4',3':5,6]pyrido[2,3-d]pyrimidine-5,7(6H)-dione (b7).*


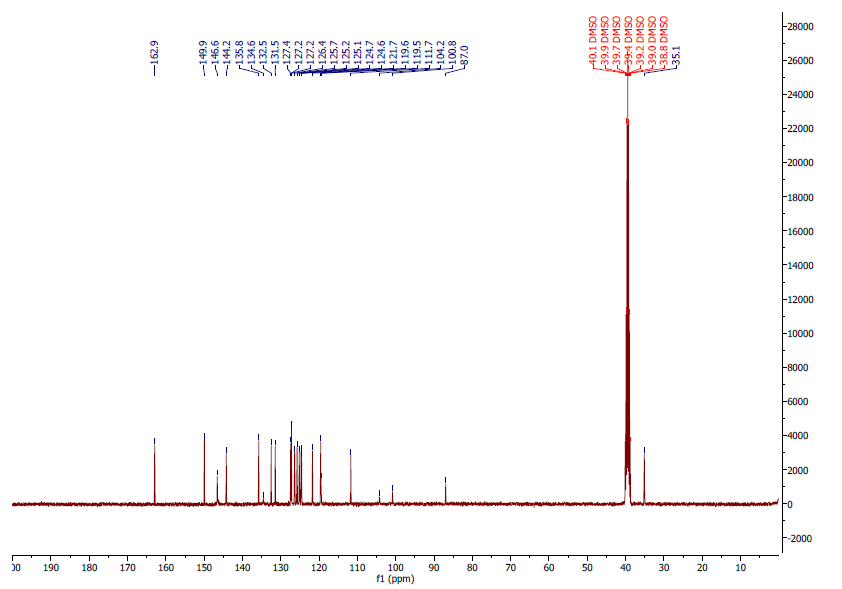


*^13^C-NMR spectrum of3-(1H-indol-3-yl)-4-(naphthalen-2-yl)-1,4,8,9-tetrahydro-5H-pyrazolo[4',3':5,6]pyrido[2,3-d]pyrimidine-5,7(6H)-dione (b7).*


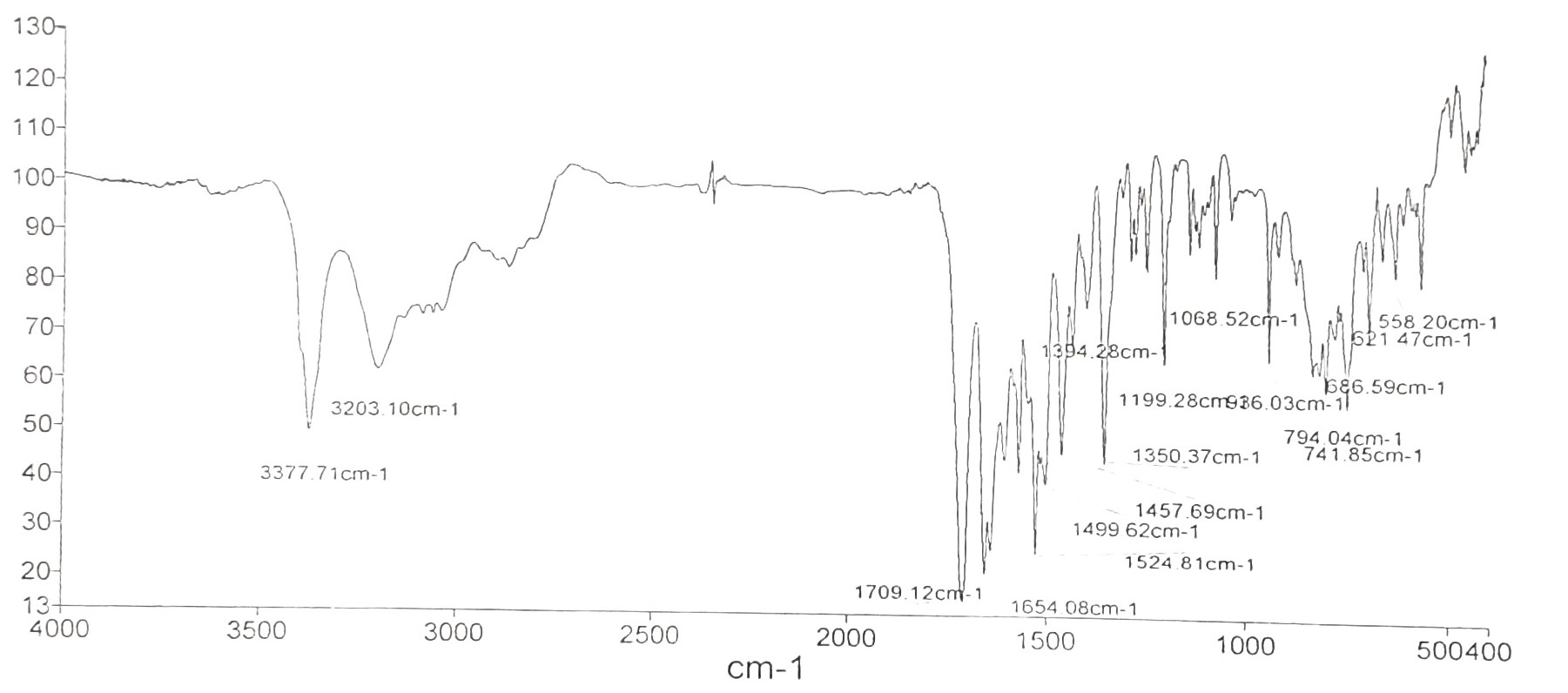


*FT-IR spectrum of 3-(1H-indol-3-yl)-4-(3-nitrophenyl)-1,4,8,9-tetrahydro-5H-pyrazolo[4',3':5,6]pyrido[2,3-d]pyrimidine-5,7(6H)-dione (b8).*


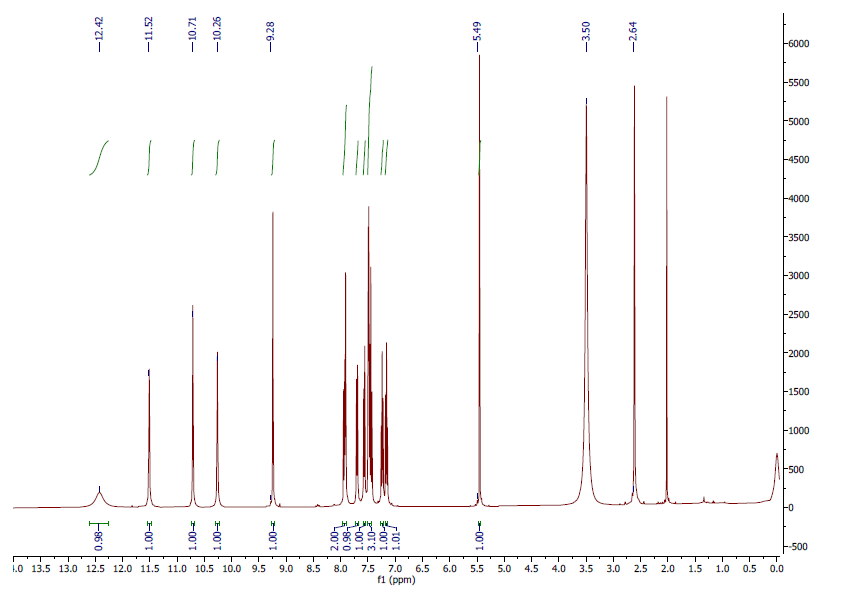


*^1^H-NMR spectrum of 3-(1H-indol-3-yl)-4-(3-nitrophenyl)-1,4,8,9-tetrahydro-5H-pyrazolo[4',3':5,6]pyrido[2,3-d]pyrimidine-5,7(6H)-dione (b8).*

*
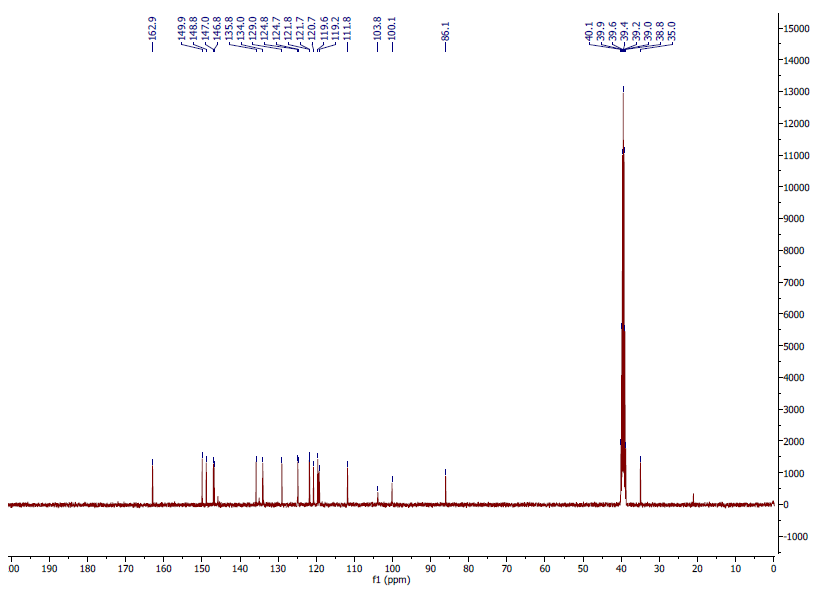
*

*^13^C-NMR spectrum of3-(1H-indol-3-yl)-4-(3-nitrophenyl)-1,4,8,9-tetrahydro-5H-pyrazolo[4',3':5,6]pyrido[2,3-d]pyrimidine-5,7(6H)-dione (b8).*


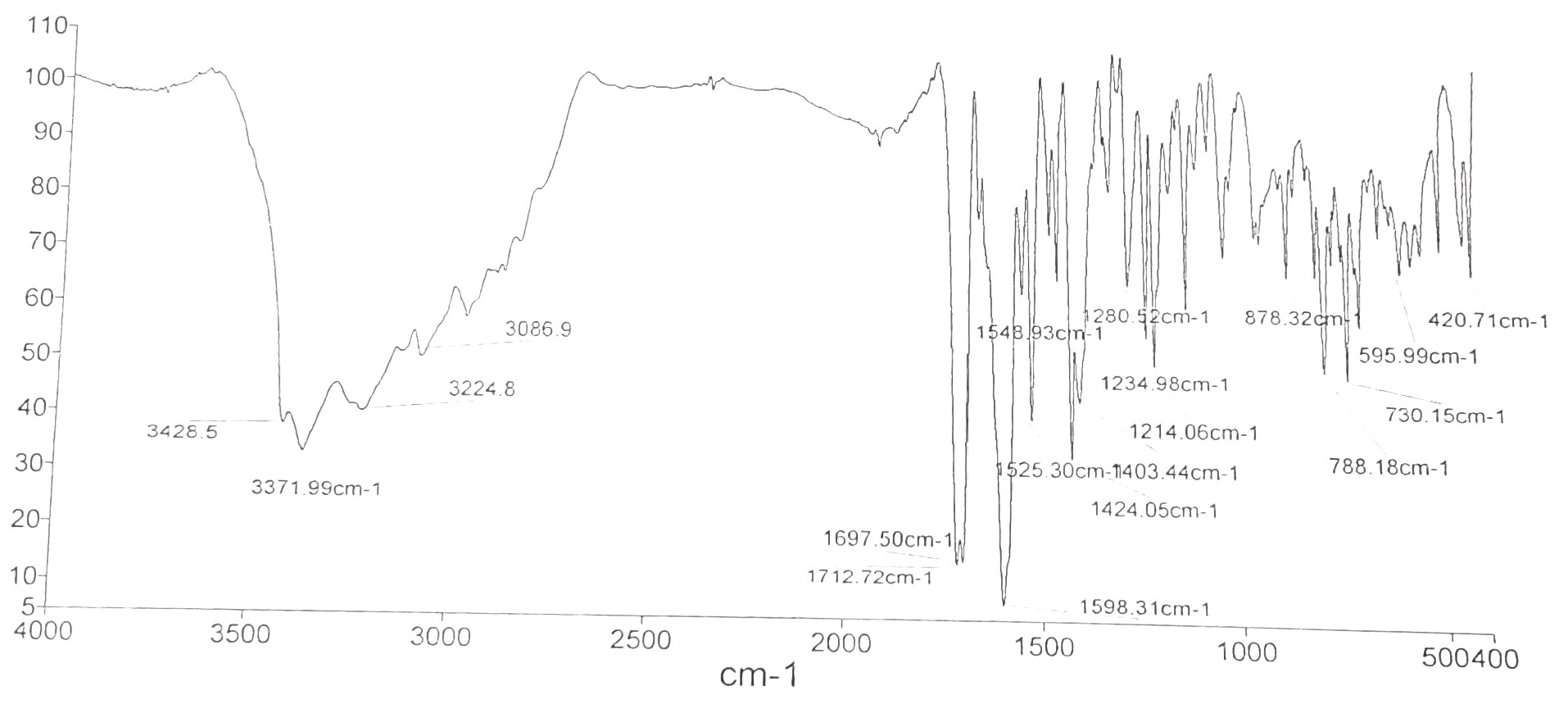


*FT-IR spectrum of 4-(3-hydroxyphenyl)-3-(1H-indol-3-yl)-1,4,8,9-tetrahydro-5H-pyrazolo[4',3':5,6]pyrido[2,3-d]pyrimidine-5,7(6H)-dione (b9).*


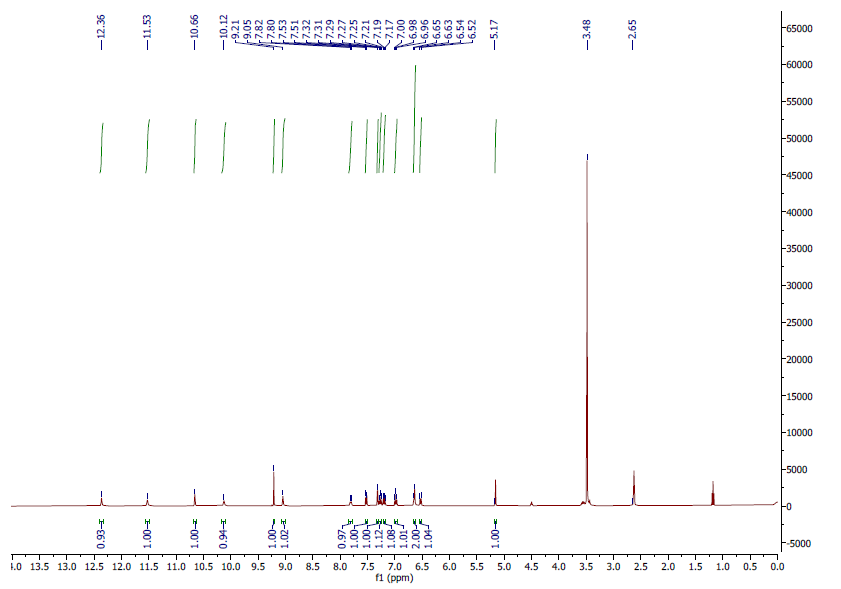


*^1^H-NMR spectrum of 4-(3-hydroxyphenyl)-3-(1H-indol-3-yl)-1,4,8,9-tetrahydro-5H-pyrazolo[4',3':5,6]pyrido[2,3-d]pyrimidine-5,7(6H)-dione (b9).*


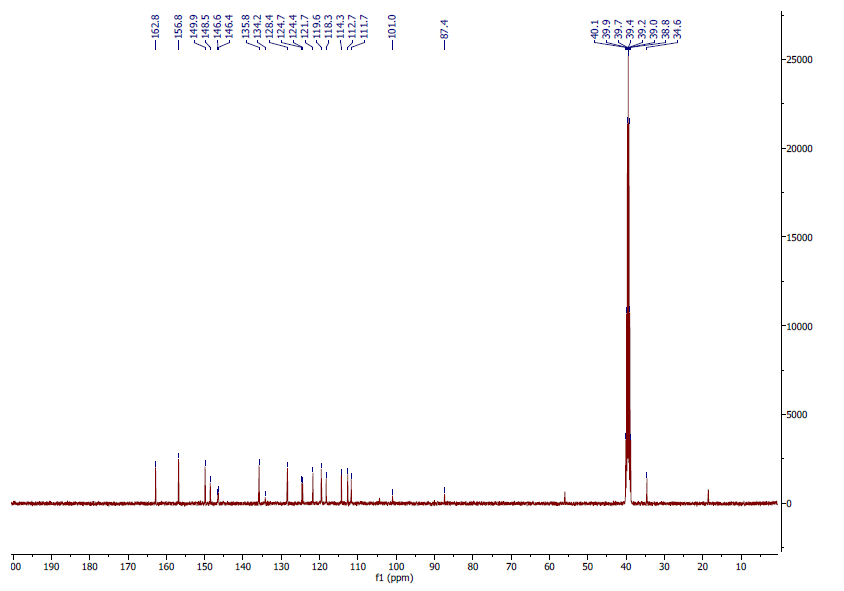


*^13^C-NMR spectrum of4-(3-hydroxyphenyl)-3-(1H-indol-3-yl)-1,4,8,9-tetrahydro-5H-pyrazolo[4',3':5,6]pyrido[2,3-d]pyrimidine-5,7(6H)-dione (b9).*


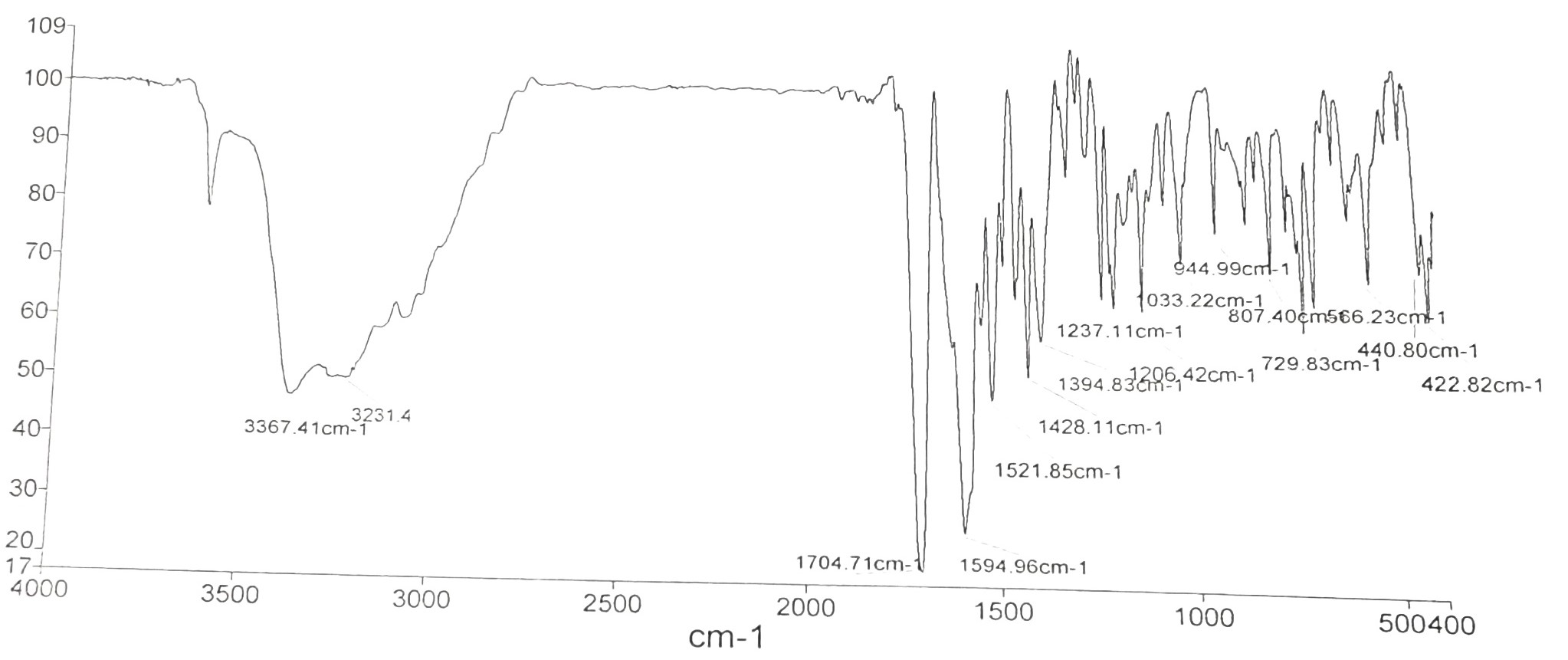


*FT-IR spectrum of 3-(1H-indol-3-yl)-4-phenyl-1,4,8,9-tetrahydro-5H-pyrazolo[4',3':5,6]pyrido[2,3-d]pyrimidine-5,7(6H)-dione (b10)*


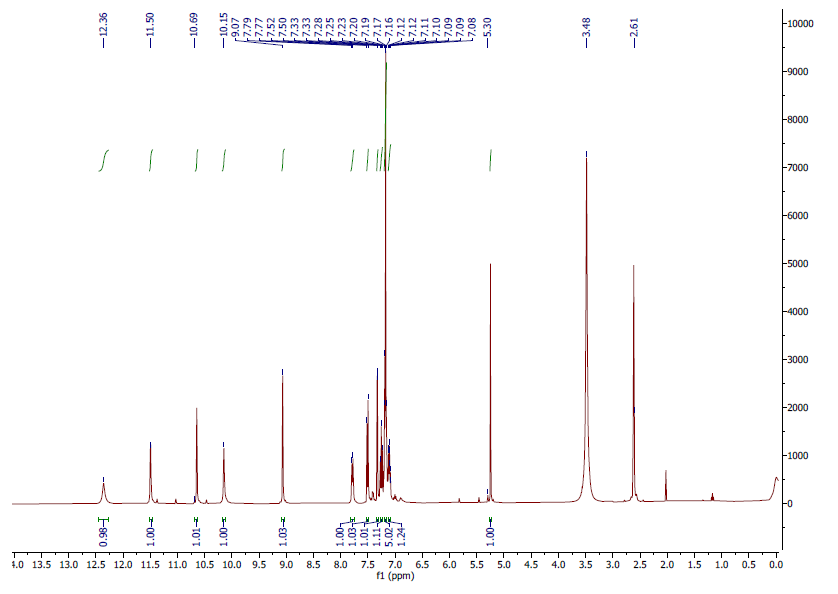


*^1^H-NMR spectrum of 3-(1H-indol-3-yl)-4-phenyl-1,4,8,9-tetrahydro-5H-pyrazolo[4',3':5,6]pyrido[2,3-d]pyrimidine-5,7(6H)-dione (b10).*


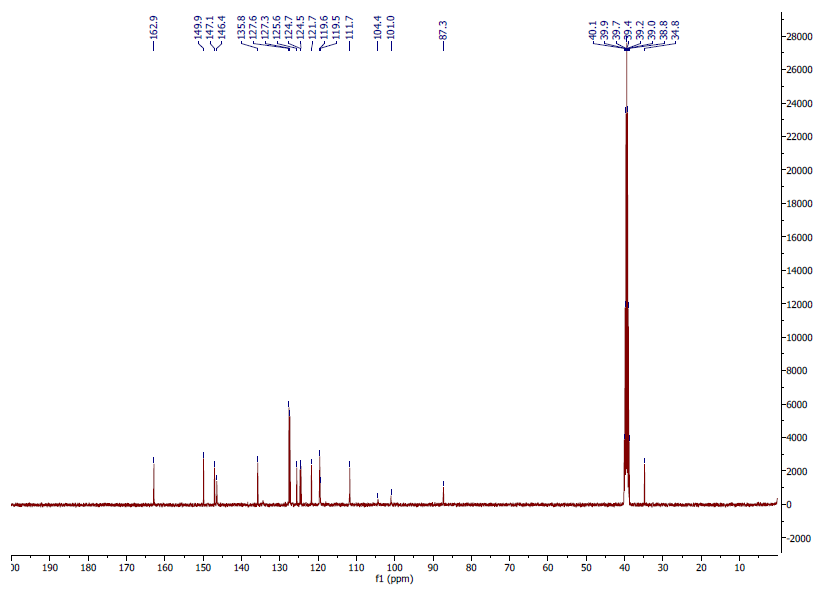


*^13^C-NMR spectrum of 3-(1H-indol-3-yl)-4-phenyl-1,4,8,9-tetrahydro-5H-pyrazolo[4',3':5,6]pyrido[2,3-d]pyrimidine-5,7(6H)-dione (b10).*


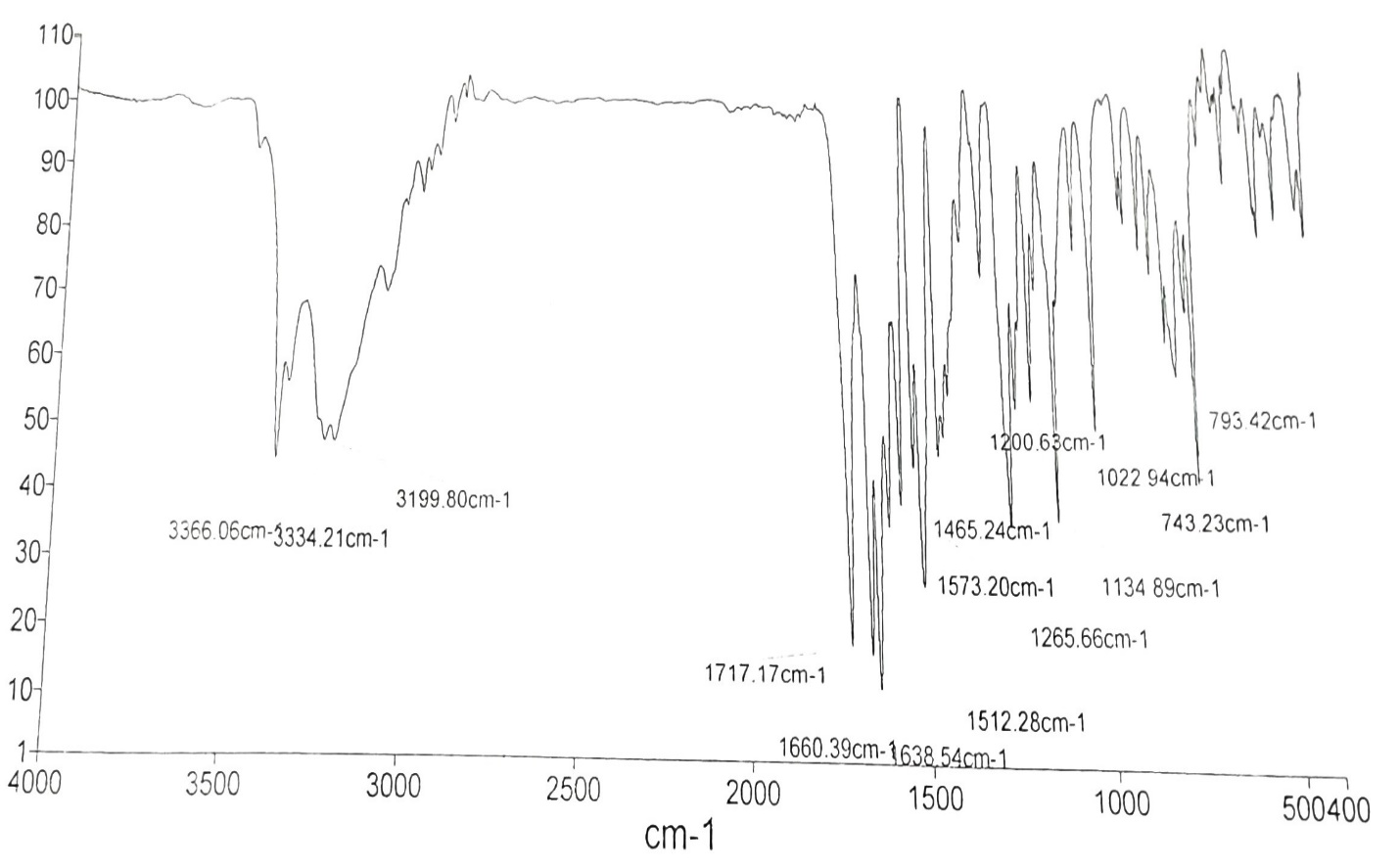


*FT-IR spectrum of 4-(2,3-dihydroxyphenyl)-3-(1H-indol-3-yl)-1,4,8,9-tetrahydro-5H-pyrazolo[4',3':5,6]pyrido[2,3-d]pyrimidine-5,7(6H)-dione (b11).*


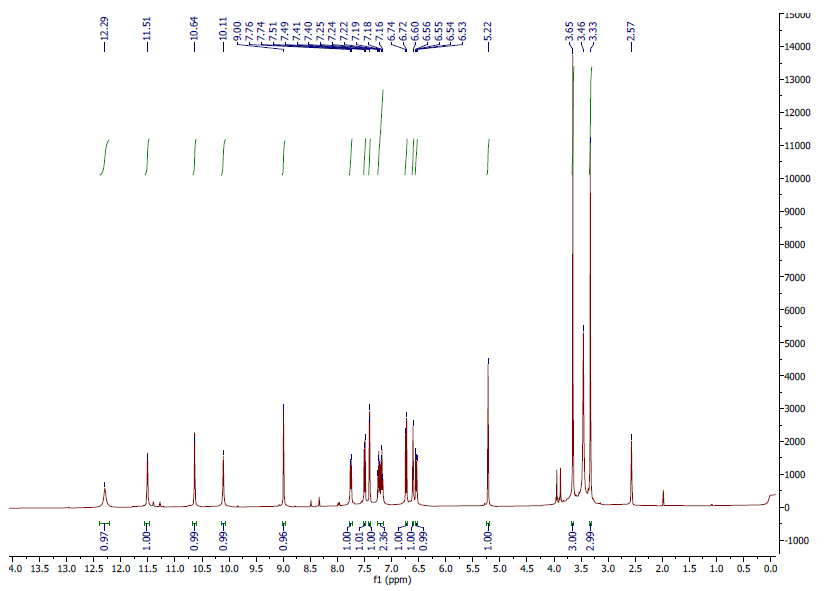


*^1^H-NMR spectrum of 4-(2,3-dihydroxyphenyl)-3-(1H-indol-3-yl)-1,4,8,9-tetrahydro-5H-pyrazolo[4',3':5,6]pyrido[2,3-d]pyrimidine-5,7(6H)-dione (b11).*


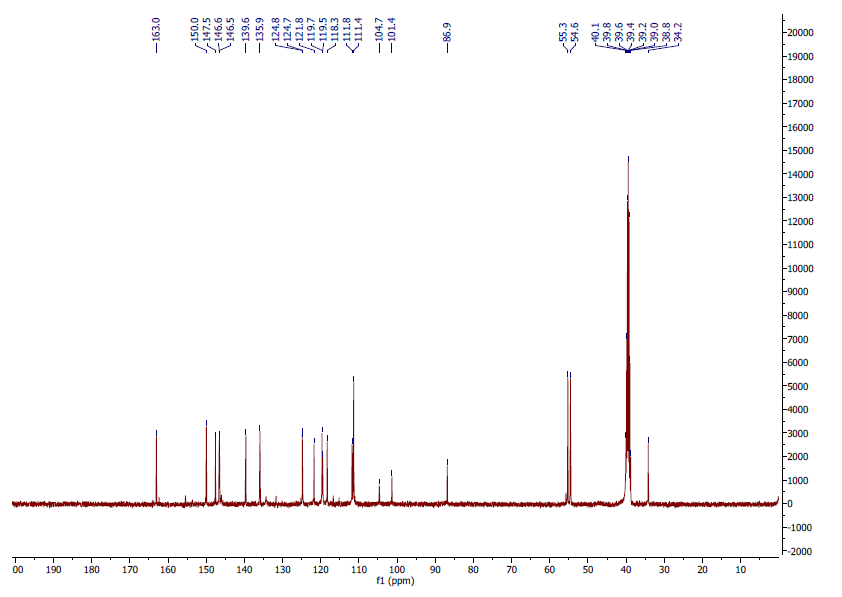


*^13^C-NMR spectrum of4-(2,3-dihydroxyphenyl)-3-(1H-indol-3-yl)-1,4,8,9-tetrahydro-5H-pyrazolo[4',3':5,6]pyrido[2,3-d]pyrimidine-5,7(6H)-dione (b11).*


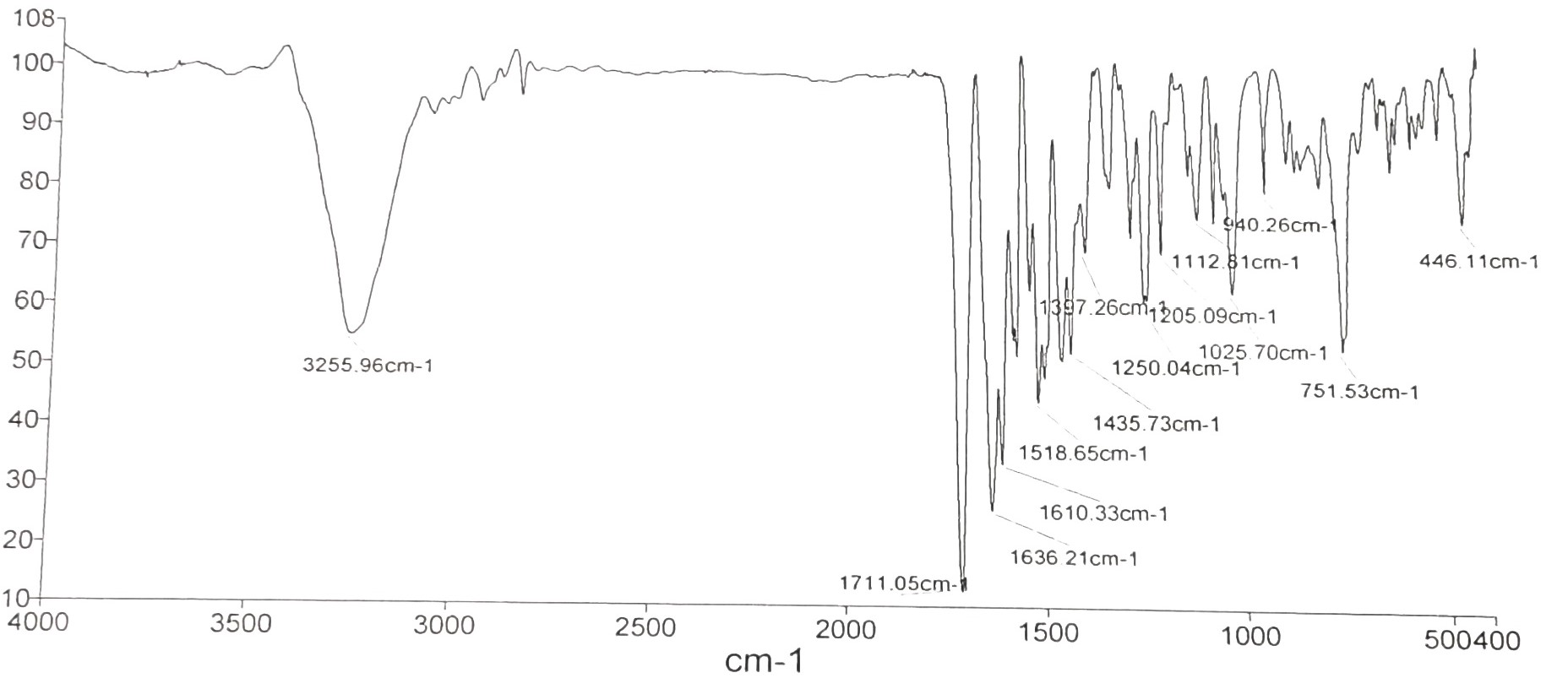


*FT-IR spectrum of 3-(1H-indol-3-yl)-4-(2-methoxyphenyl)-1,4,8,9-tetrahydro-5H-pyrazolo[4',3':5,6]pyrido[2,3-d]pyrimidine-5,7(6H)-dione (b12).*


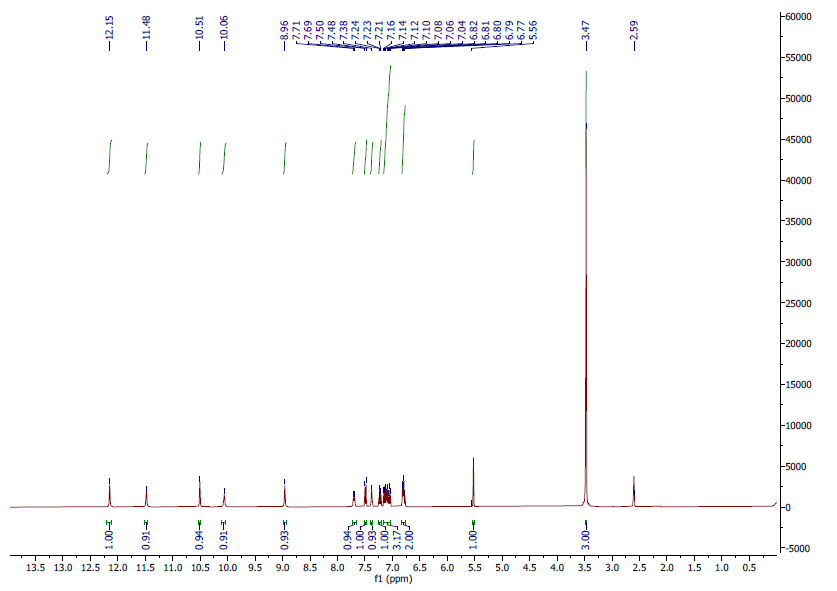


*^1^H-NMR spectrum of 3-(1H-indol-3-yl)-4-(2-methoxyphenyl)-1,4,8,9-tetrahydro-5H-pyrazolo[4',3':5,6]pyrido[2,3-d]pyrimidine-5,7(6H)-dione (b12).*


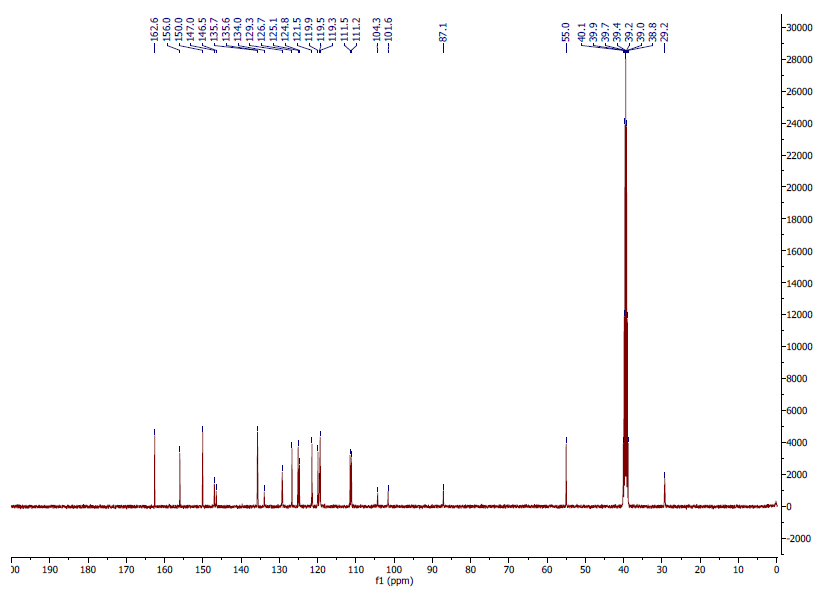


*^13^C-NMR spectrum of3-(1H-indol-3-yl)-4-(2-methoxyphenyl)-1,4,8,9-tetrahydro-5H-pyrazolo[4',3':5,6]pyrido[2,3-d]pyrimidine-5,7(6H)-dione (b12).*


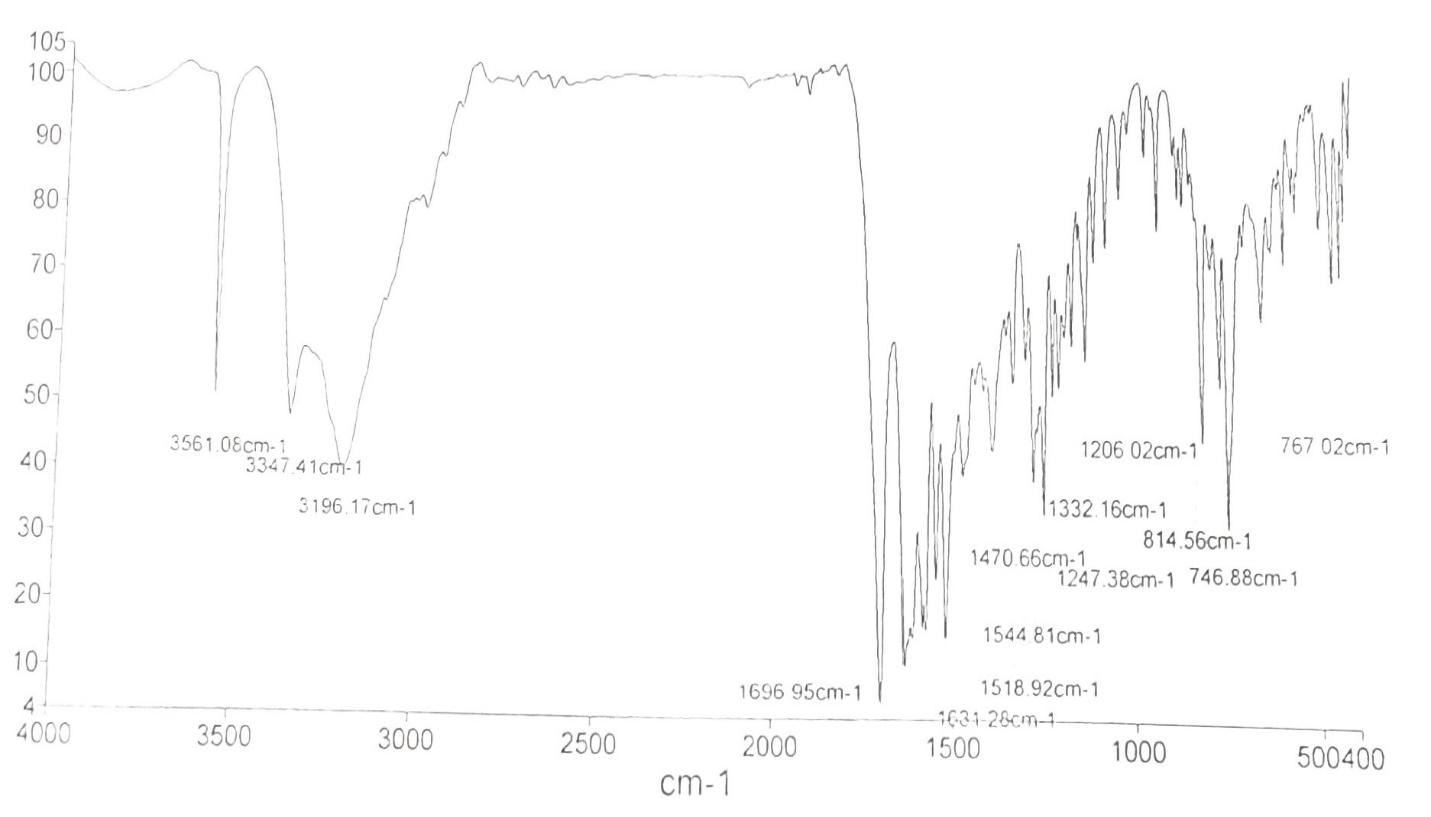


*FT-IR spectrum of 4-(2,3-dihydroxyphenyl)-3-(1H-indol-3-yl)-1,4,8,9-tetrahydro-5H-pyrazolo[4',3':5,6]pyrido[2,3-d]pyrimidine-5,7(6H)-dione (b13).*


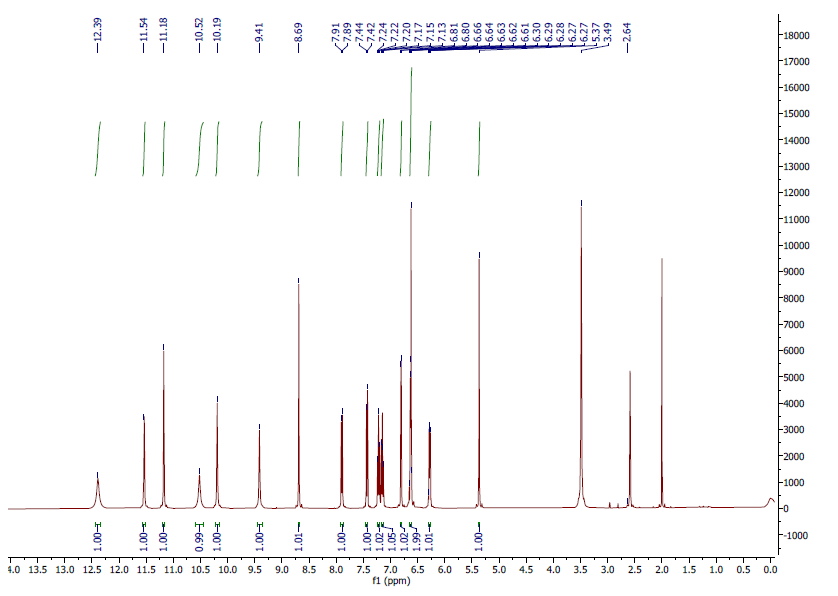


*^1^H-NMR spectrum of 4-(2,3-dihydroxyphenyl)-3-(1H-indol-3-yl)-1,4,8,9-tetrahydro-5H-pyrazolo[4',3':5,6]pyrido[2,3-d]pyrimidine-5,7(6H)-dione (b13).*


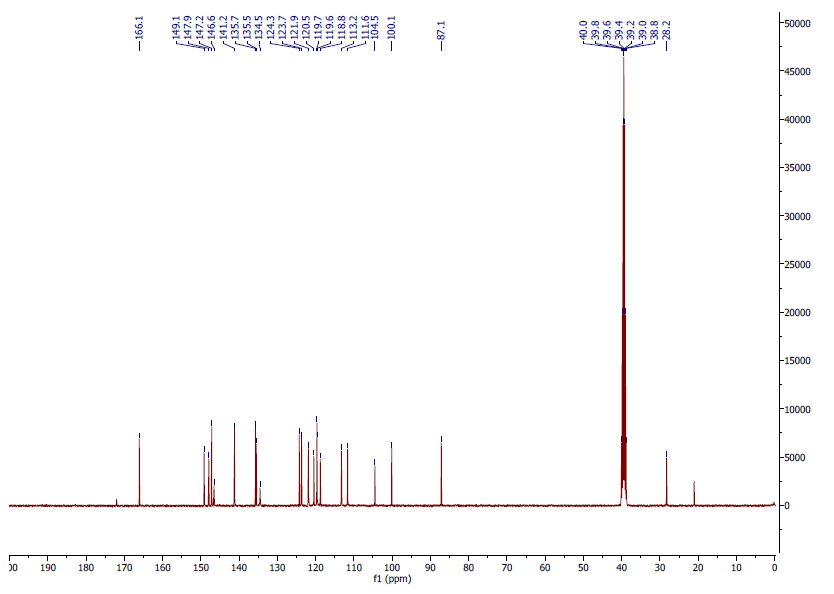


*^13^C-NMR spectrum of4-(2,3-dihydroxyphenyl)-3-(1H-indol-3-yl)-1,4,8,9-tetrahydro-5H-pyrazolo[4',3':5,6]pyrido[2,3-d]pyrimidine-5,7(6H)-dione (b13).*


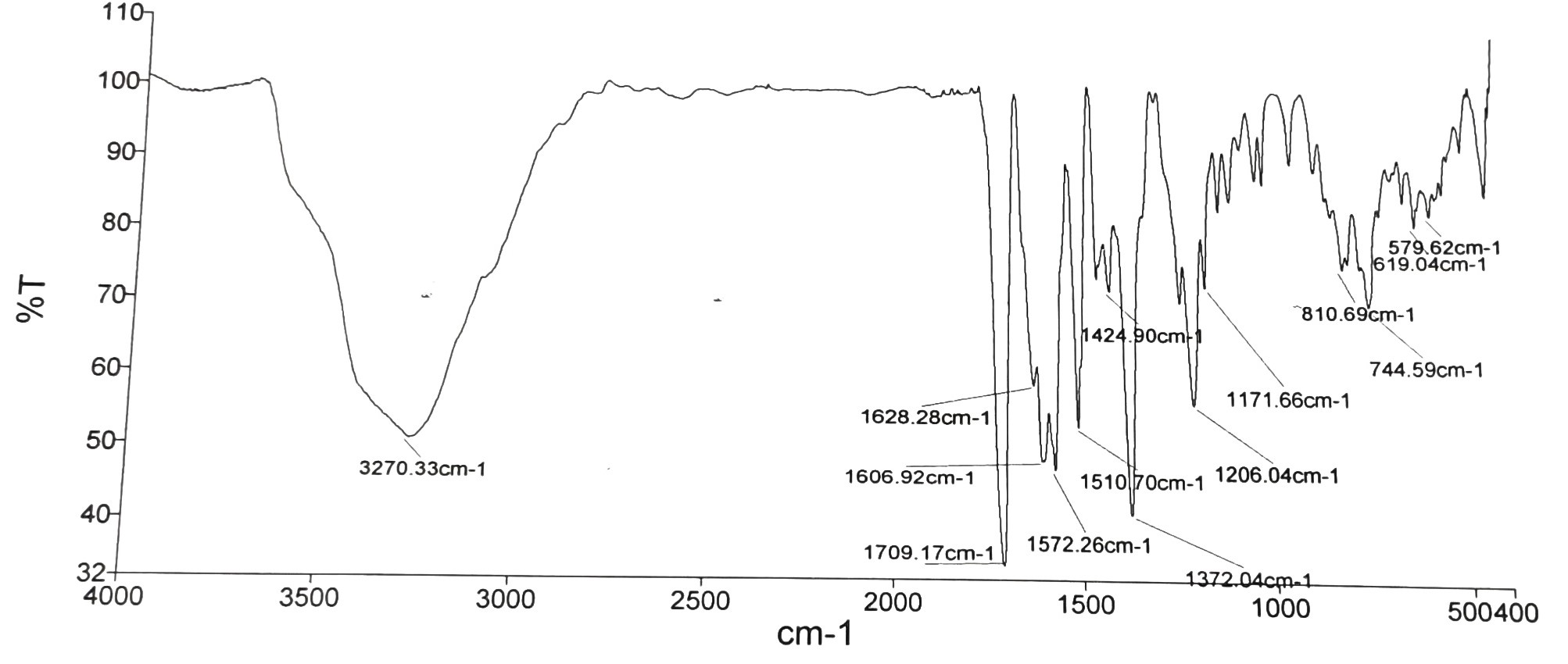


*FT-IR spectrum of 4,4'-(1,4-phenylene)bis(3-(1H-indol-3-yl)-1,4,8,9-tetrahydro-5H-pyrazolo[4',3':5,6]pyrido[2,3-d]pyrimidine-5,7(6H)-dione) (b14).*


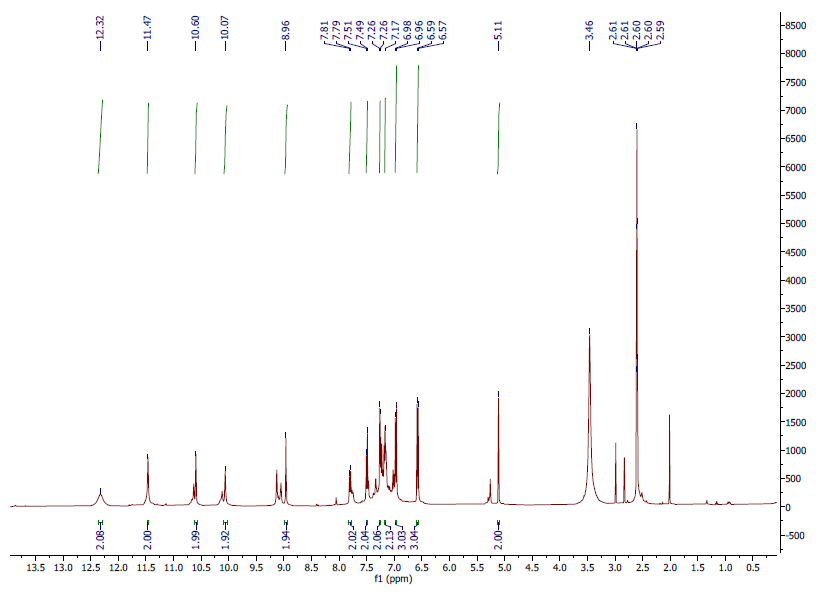


*^1^H-NMR spectrum of 4,4'-(1,4-phenylene)bis(3-(1H-indol-3-yl)-1,4,8,9-tetrahydro-5H-pyrazolo[4',3':5,6]pyrido[2,3-d]pyrimidine-5,7(6H)-dione) (b14).*


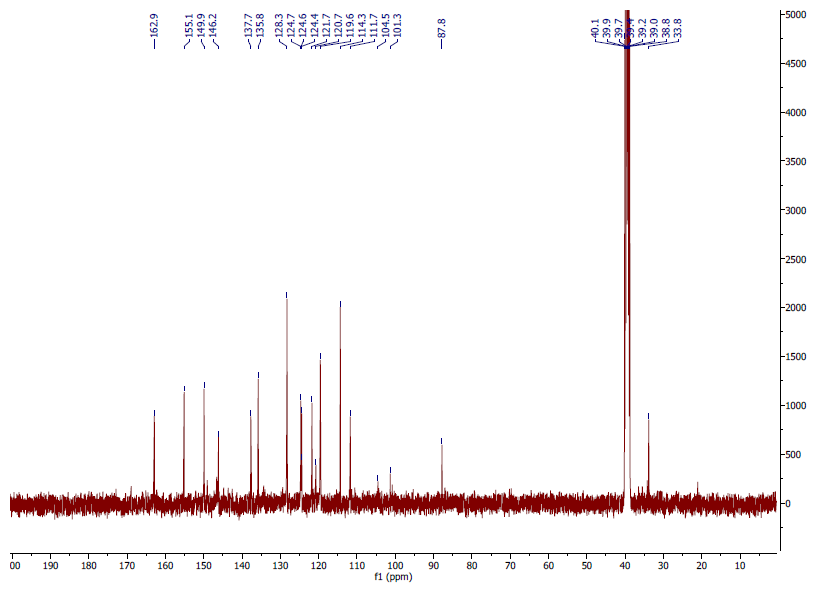


*^13^C-NMR spectrum of 4,4'-(1,4-phenylene)bis(3-(1H-indol-3-yl)-1,4,8,9-tetrahydro-5H-pyrazolo[4',3':5,6]pyrido[2,3-d]pyrimidine-5,7(6H)-dione) (b14).*


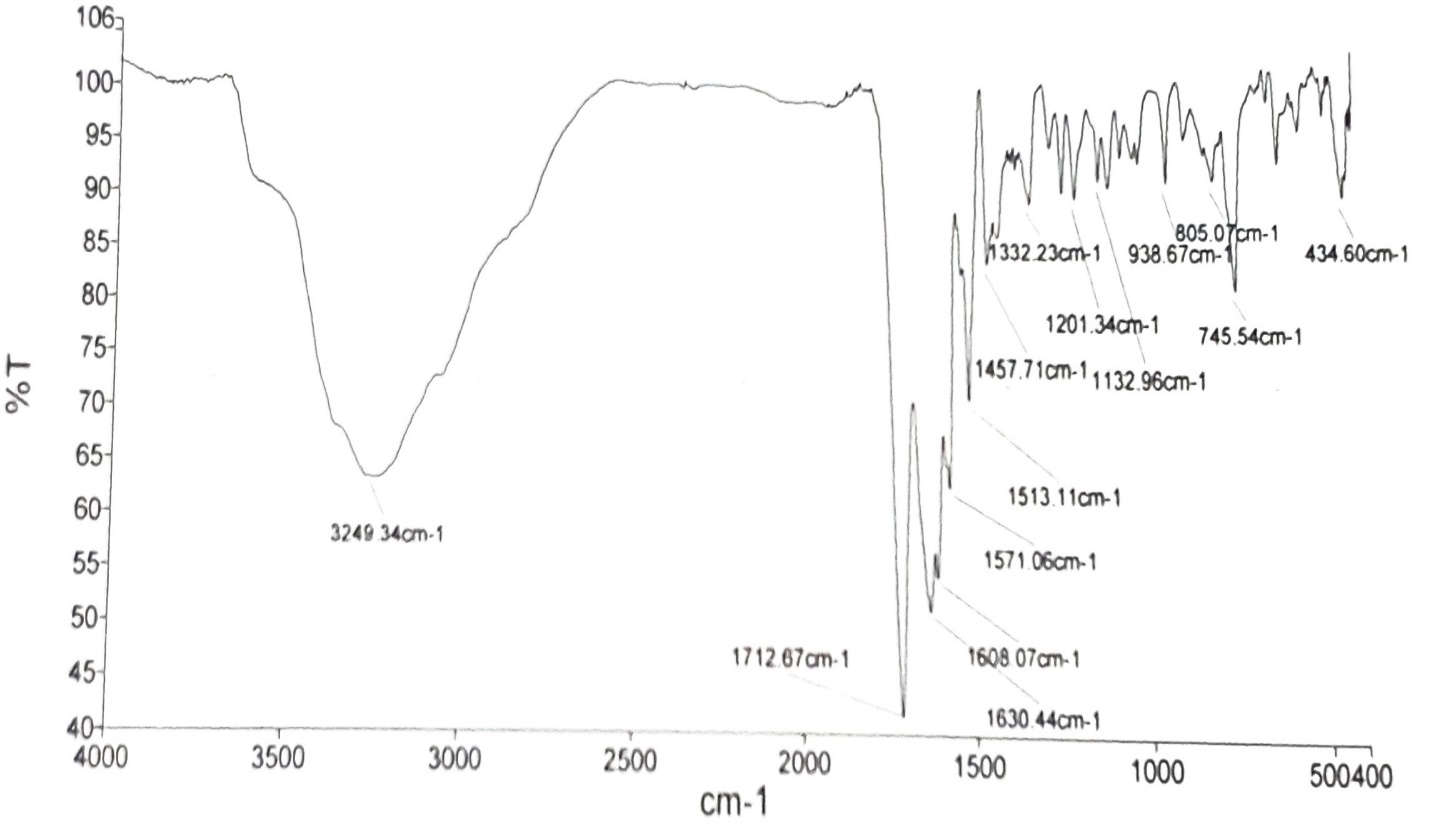


*FT-IR spectrum of 4,4',4''-(((1,3,5-triazine-2,4,6-triyl)tris(oxy))tris(benzene-4,1-diyl))tris(3-(1H-indol-3-yl)-1,4,8,9-tetrahydro-5H-pyrazolo[4',3':5,6]pyrido[2,3-d]pyrimidine-5,7(6H)-dione) (b15).*


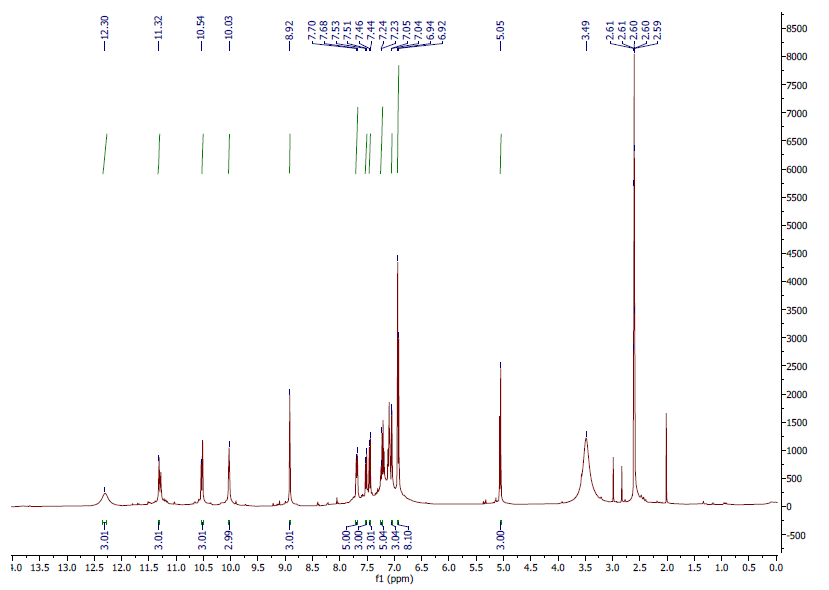


*^1^H-NMR spectrum of 4,4',4''-(((1,3,5-triazine-2,4,6-triyl)tris(oxy))tris(benzene-4,1-diyl))tris(3-(1H-indol-3-yl)-1,4,8,9-tetrahydro-5H-pyrazolo[4',3':5,6]pyrido[2,3-d]pyrimidine-5,7(6H)-dione) (b15.)*


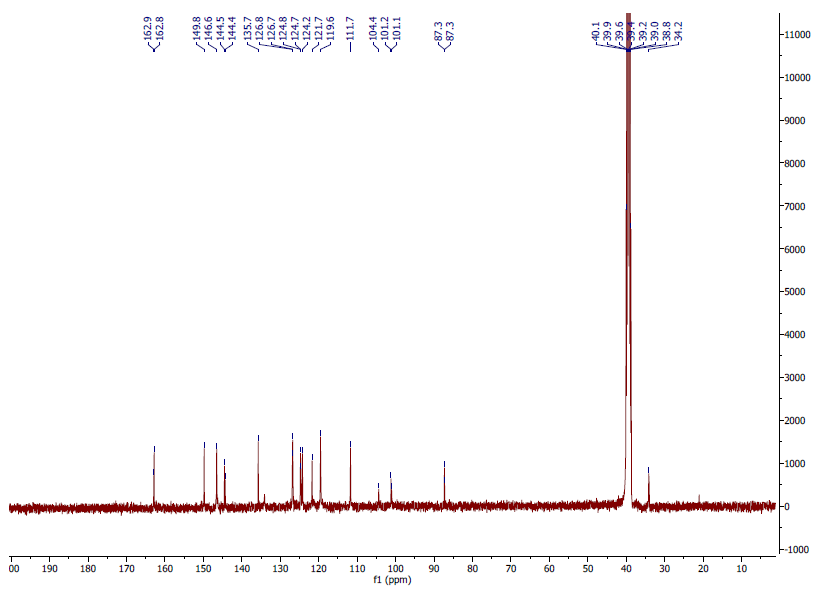


*^13^C-NMR spectrum of 4,4',4''-(((1,3,5-triazine-2,4,6-triyl)tris(oxy))tris(benzene-4,1-diyl))tris(3-(1H-indol-3-yl)-1,4,8,9-tetrahydro-5H-pyrazolo[4',3':5,6]pyrido[2,3-d]pyrimidine-5,7(6H)-dione) (b15).*


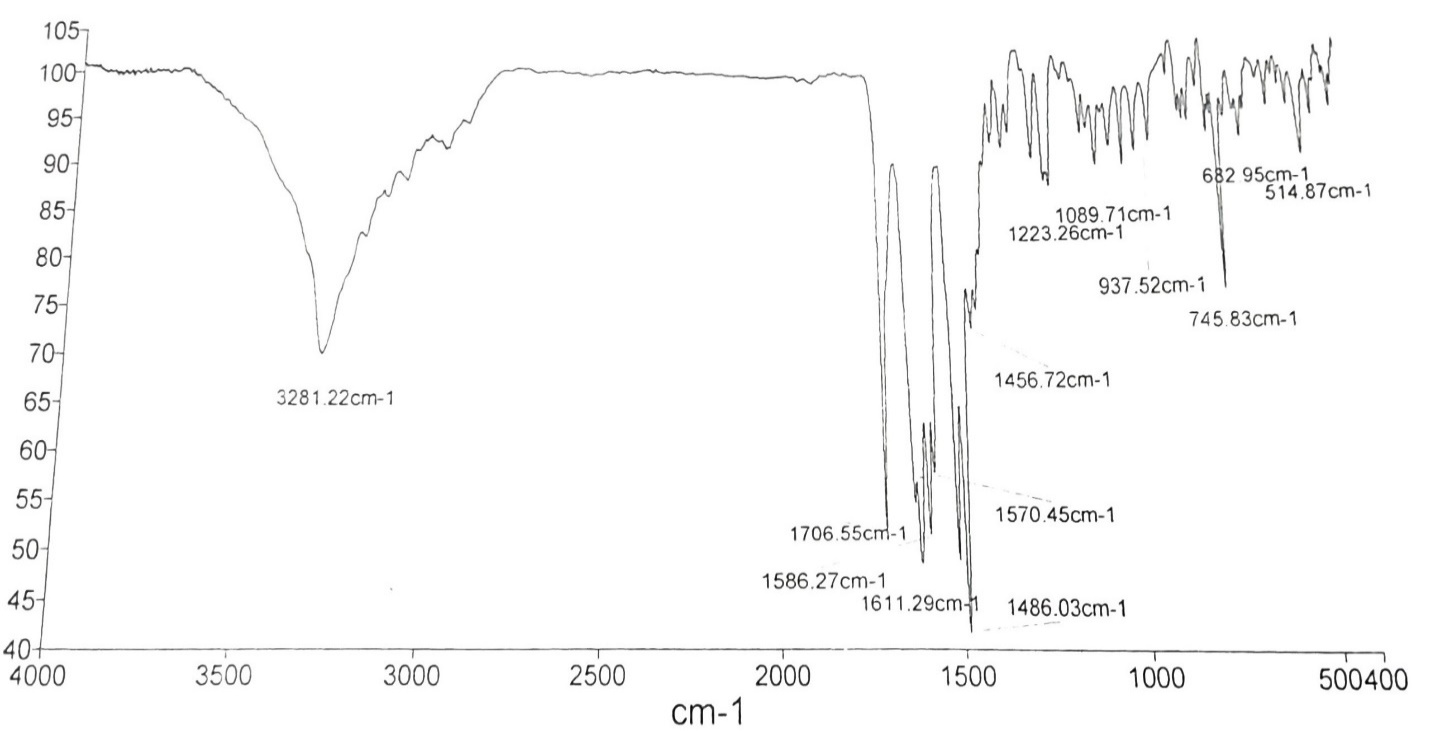


*FT-IR spectrum of 4-(4-chlorophenyl)-3-(1H-indol-3-yl)-6,8-dimethyl-1,4,8,9-tetrahydro-5H-pyrazolo[4',3':5,6]pyrido[2,3-d]pyrimidine-5,7(6H)-dione (c1).*


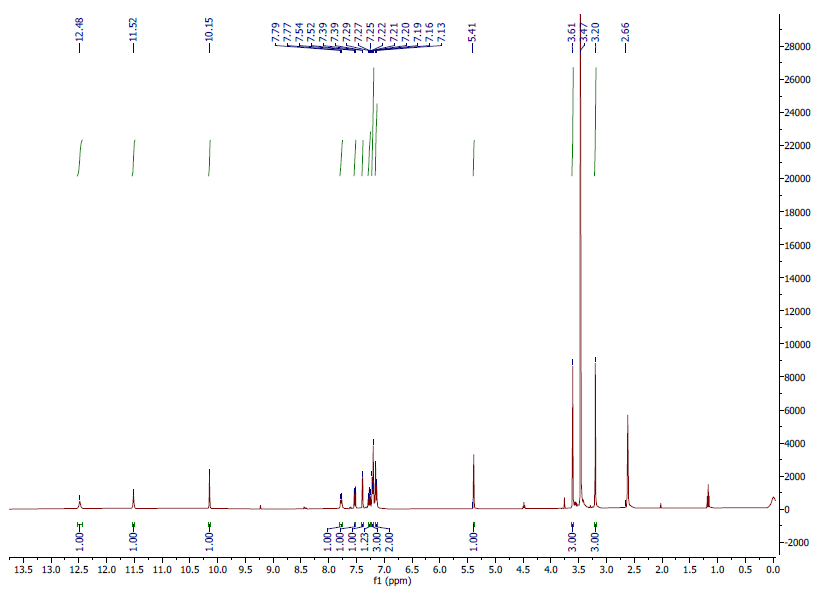


*^1^H-NMR spectrum of 4-(4-chlorophenyl)-3-(1H-indol-3-yl)-6,8-dimethyl-1,4,8,9-tetrahydro-5H-pyrazolo[4',3':5,6]pyrido[2,3-d]pyrimidine-5,7(6H)-dione (c1).*


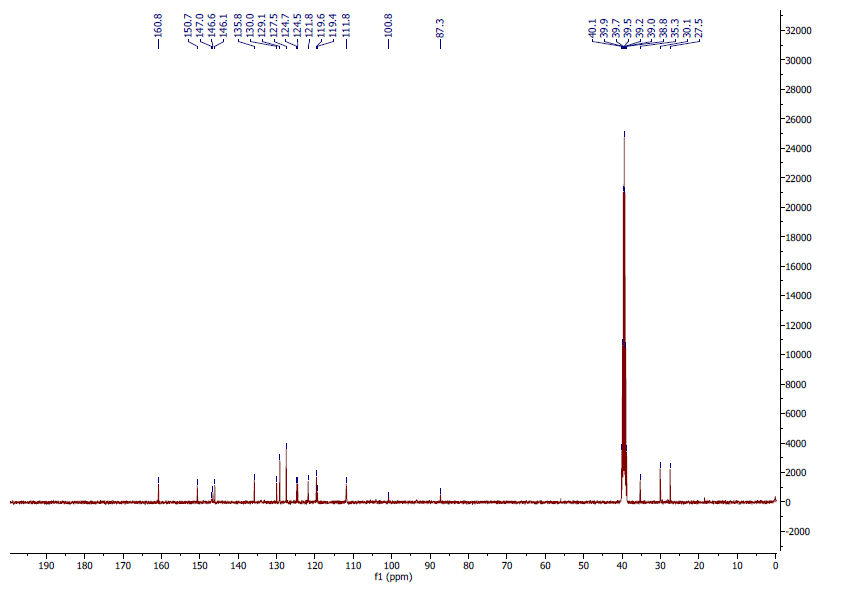


*^13^C-NMR spectrum of 4-(4-chlorophenyl)-3-(1H-indol-3-yl)-6,8-dimethyl-1,4,8,9-tetrahydro-5H-pyrazolo[4',3':5,6]pyrido[2,3-d]pyrimidine-5,7(6H)-dione (c1).*


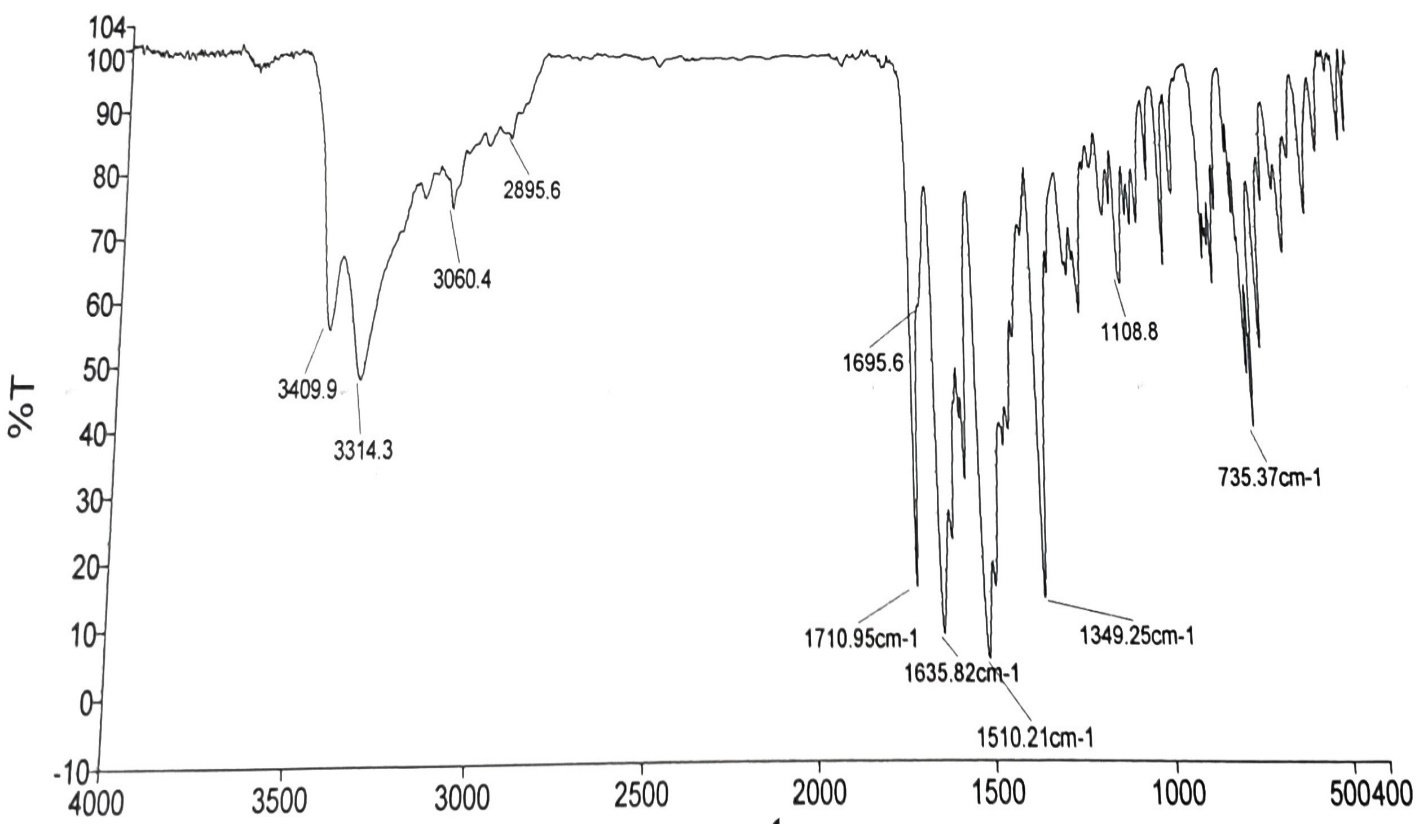


*FT-IR spectrum of 3-(1H-indol-3-yl)-6,8-dimethyl-4-(4-nitrophenyl)-1,4,8,9-tetrahydro-5H-pyrazolo[4',3':5,6]pyrido[2,3-d]pyrimidine-5,7(6H)-dione (c2).*


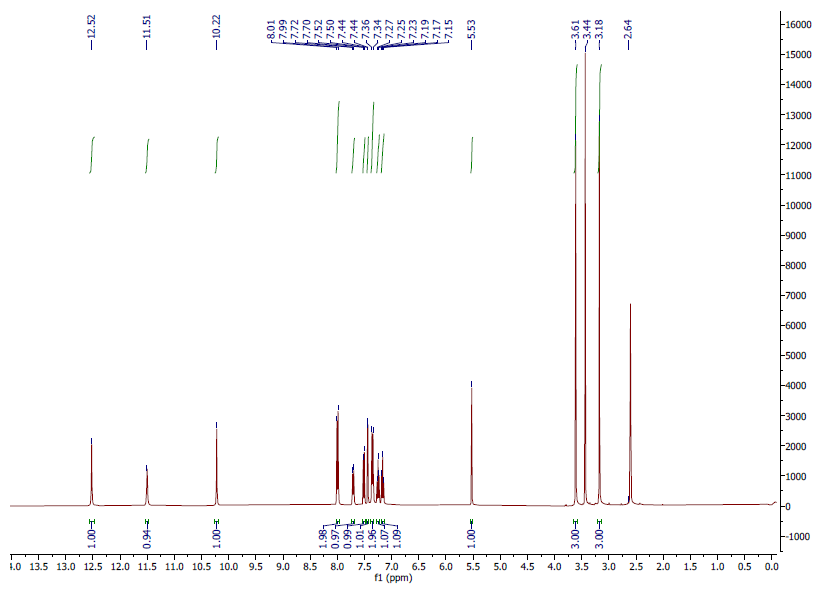


*^1^H-NMR spectrum of 3-(1H-indol-3-yl)-6,8-dimethyl-4-(4-nitrophenyl)-1,4,8,9-tetrahydro-5H-pyrazolo[4',3':5,6]pyrido[2,3-d]pyrimidine-5,7(6H)-dione (c2).*


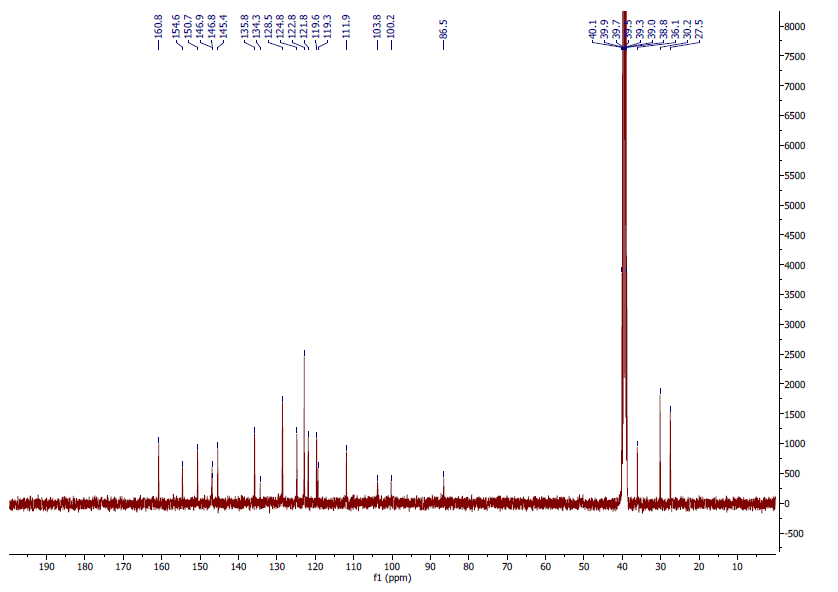


*^13^C-NMR spectrum of 3-(1H-indol-3-yl)-6,8-dimethyl-4-(4-nitrophenyl)-1,4,8,9-tetrahydro-5H-pyrazolo[4',3':5,6]pyrido[2,3-d]pyrimidine-5,7(6H)-dione (c2).*


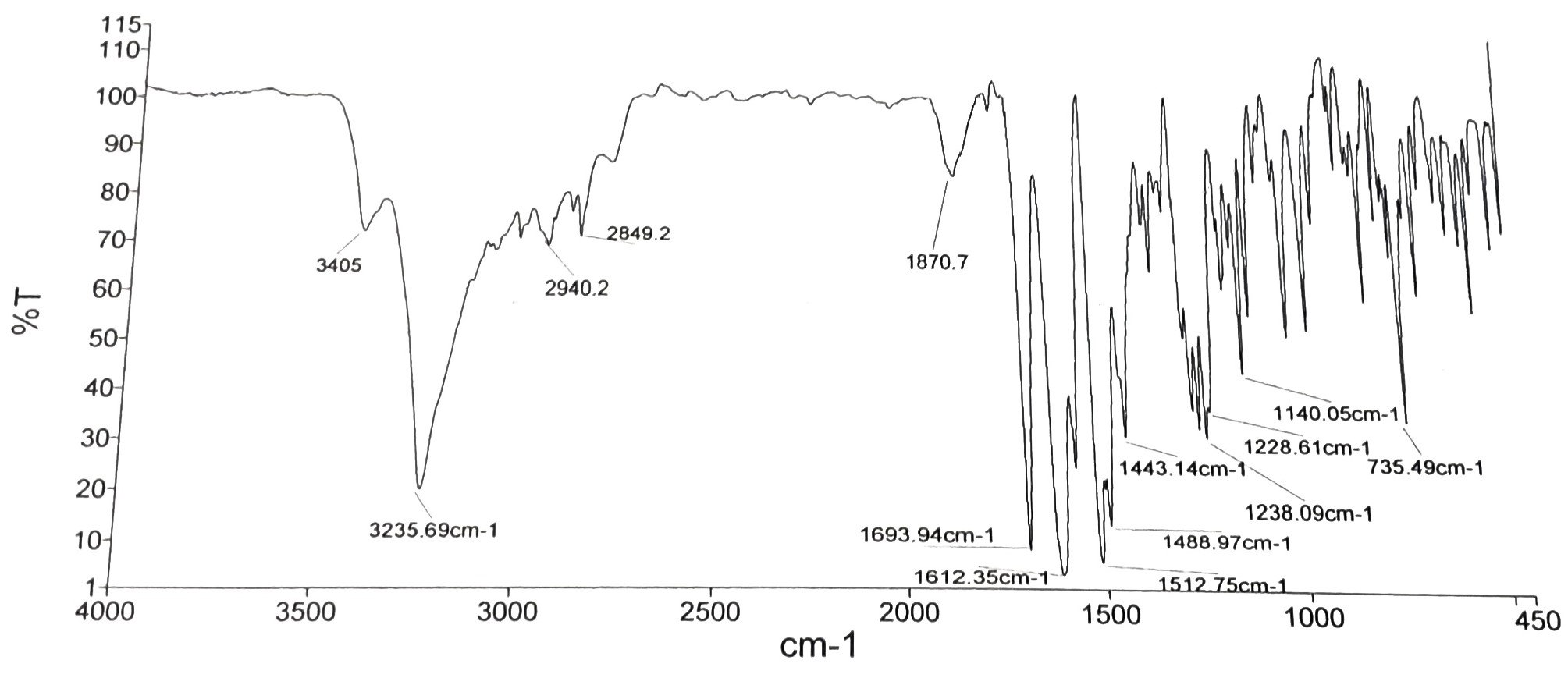


*FT-IR spectrum of* *3-(1H-indol-3-yl)-6,8-dimethyl-4-(m-tolyl)-1,4,8,9-tetrahydro-5H-pyrazolo[4',3':5,6]pyrido[2,3-d]pyrimidine-5,7(6H)-dione (c3).*

*
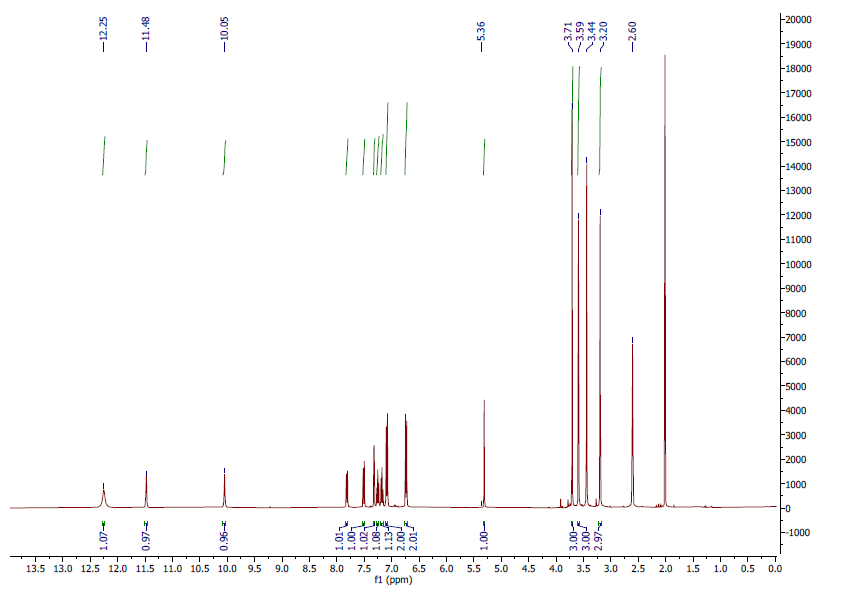
*

*^1^H-NMR spectrum of 3-(1H-indol-3-yl)-6,8-dimethyl-4-(m-tolyl)-1,4,8,9-tetrahydro-5H-pyrazolo[4',3':5,6]pyrido[2,3-d]pyrimidine-5,7(6H)-dione (c3).*

*
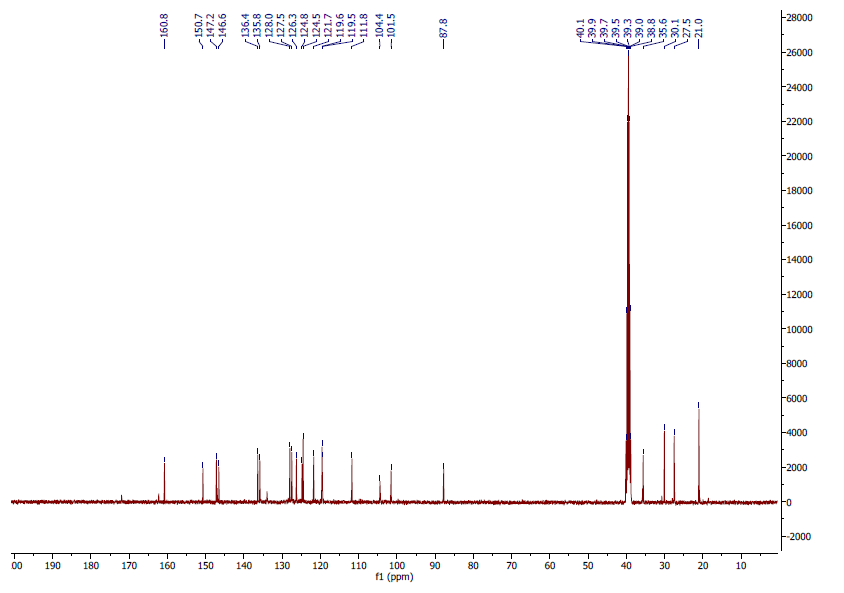
*

*^13^C-NMR spectrum of3-(1H-indol-3-yl)-6,8-dimethyl-4-(m-tolyl)-1,4,8,9-tetrahydro-5H-pyrazolo[4',3':5,6]pyrido[2,3-d]pyrimidine-5,7(6H)-dione (c3).*


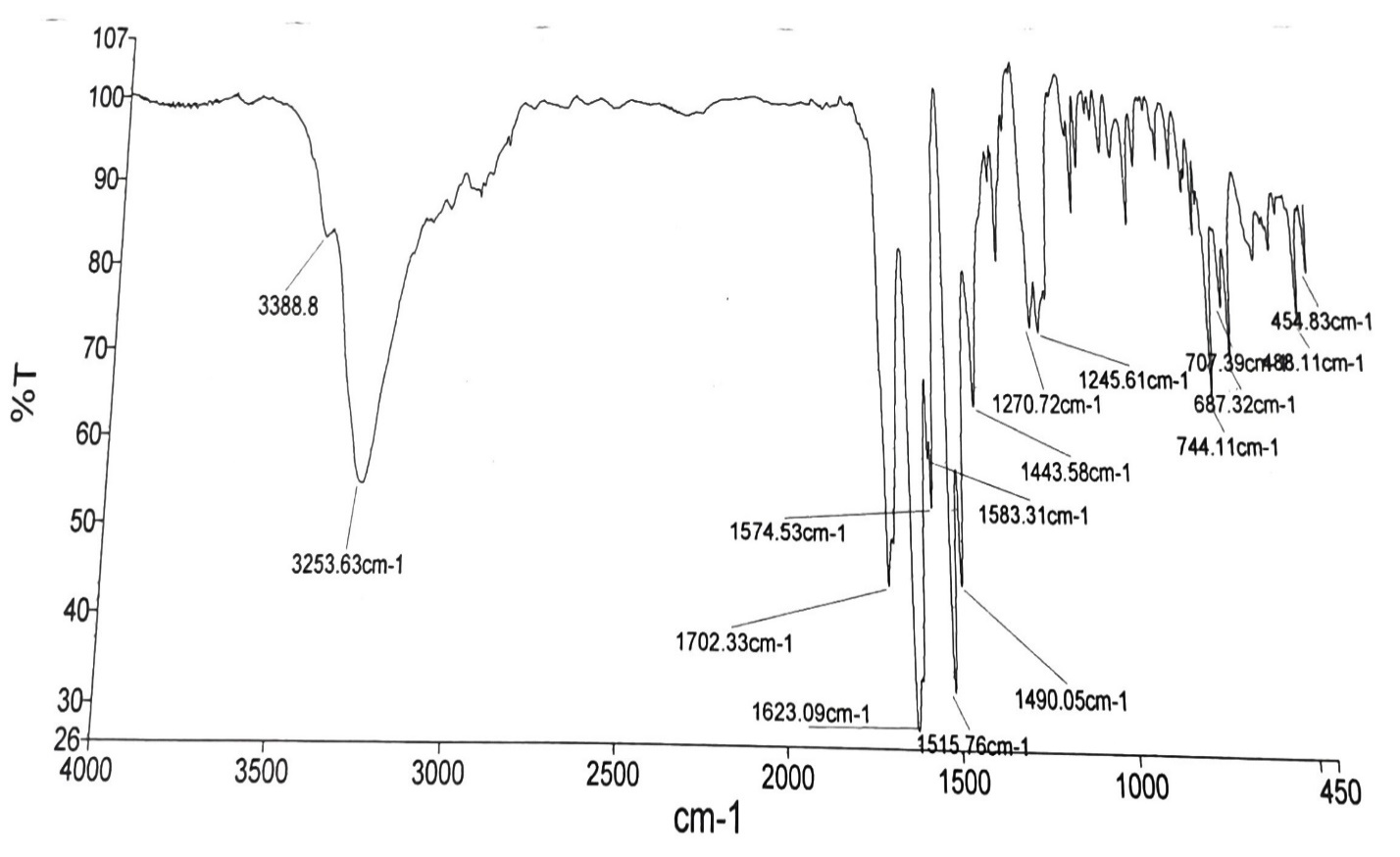


*FT-IR spectrum of* *3-(1H-indol-3-yl)-4-(4-methoxyphenyl)-6,8-dimethyl-1,4,8,9-tetrahydro-5H-pyrazolo[4',3':5,6]pyrido[2,3-d]pyrimidine-5,7(6H)-dione (c4).*


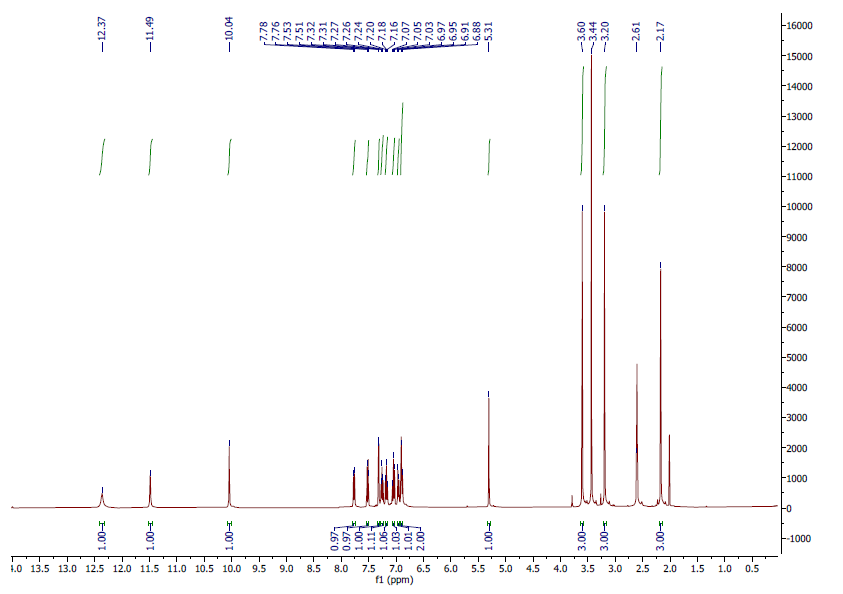


*^1^H-NMR spectrum of 3-(1H-indol-3-yl)-4-(4-methoxyphenyl)-6,8-dimethyl-1,4,8,9-tetrahydro-5H-pyrazolo[4',3':5,6]pyrido[2,3-d]pyrimidine-5,7(6H)-dione (c4).*

*
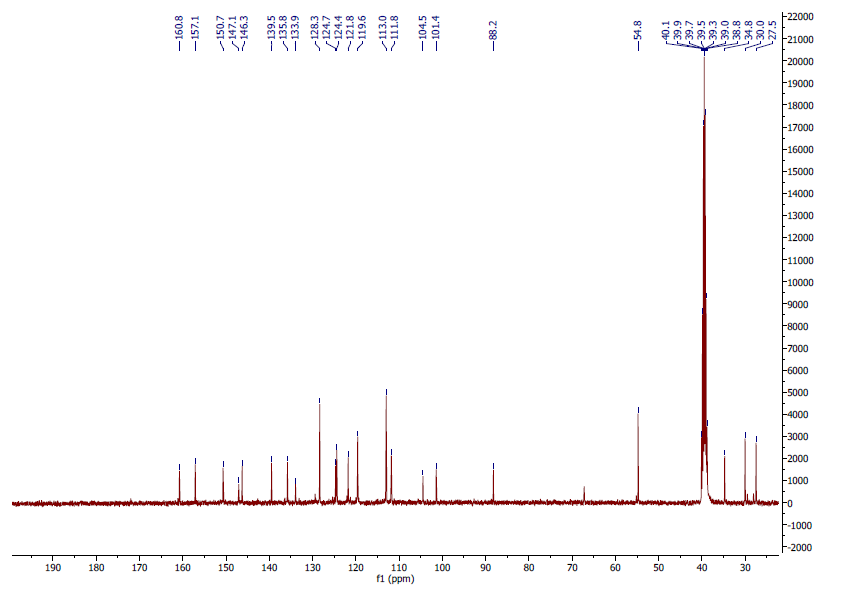
*

*^13^C-NMR spectrum of 3-(1H-indol-3-yl)-4-(4-methoxyphenyl)-6,8-dimethyl-1,4,8,9-tetrahydro-5H-pyrazolo[4',3':5,6]pyrido[2,3-d]pyrimidine-5,7(6H)-dione (c4).*


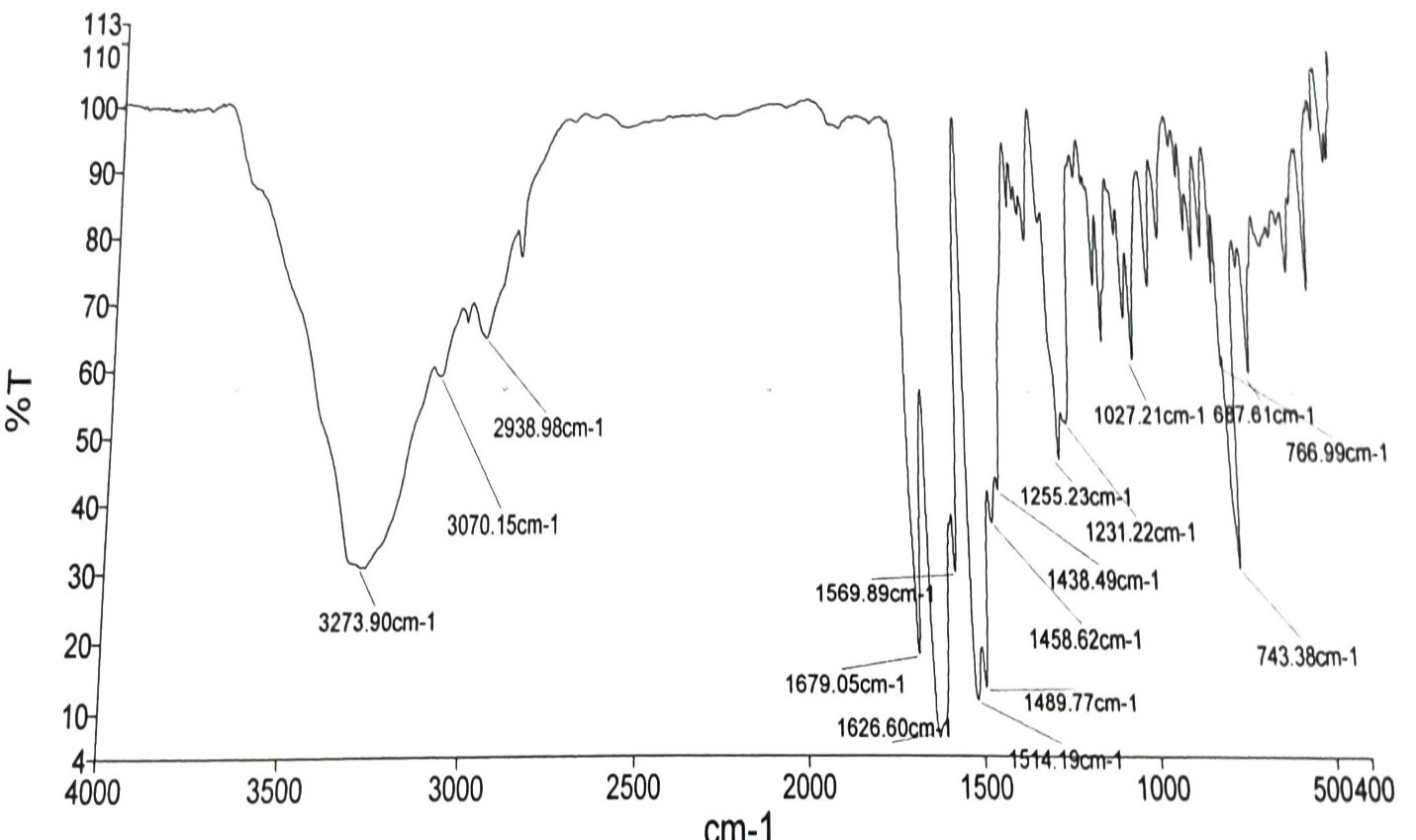


*FT-IR spectrum of 3-(1H-indol-3-yl)-4-(2-methoxyphenyl)-6,8-dimethyl-1,4,8,9-tetrahydro-5H-pyrazolo[4',3':5,6]pyrido[2,3-d]pyrimidine-5,7(6H)-dione (c5).*

*
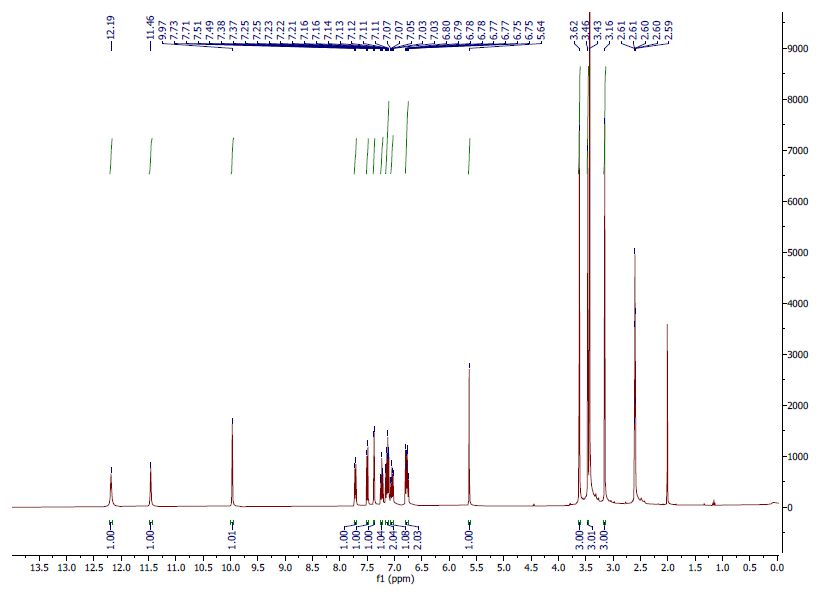
*

*^1^H-NMR spectrum of 3-(1H-indol-3-yl)-4-(2-methoxyphenyl)-6,8-dimethyl-1,4,8,9-tetrahydro-5H-pyrazolo[4',3':5,6]pyrido[2,3-d]pyrimidine-5,7(6H)-dione (c5).*


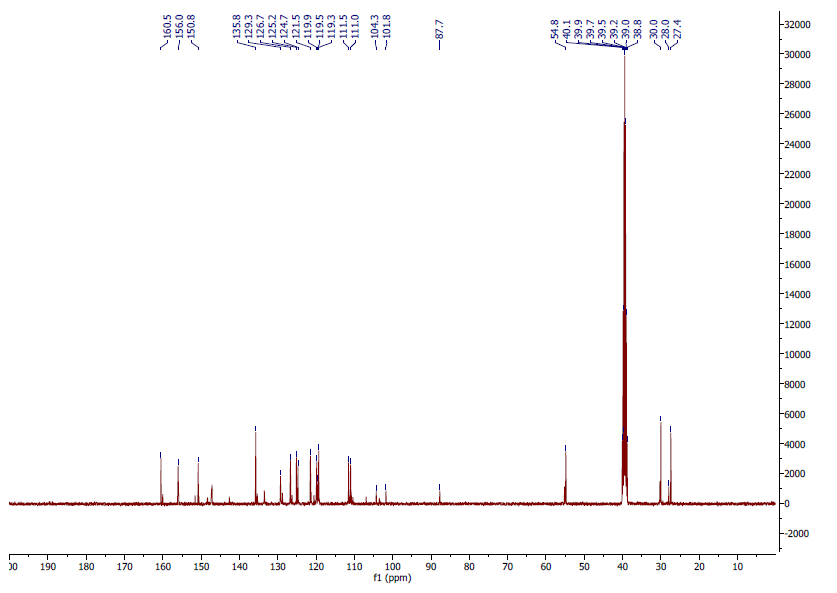


*^13^C-NMR spectrum of3-(1H-indol-3-yl)-4-(2-methoxyphenyl)-6,8-dimethyl-1,4,8,9-tetrahydro-5H-pyrazolo[4',3':5,6]pyrido[2,3-d]pyrimidine-5,7(6H)-dione (c5).*


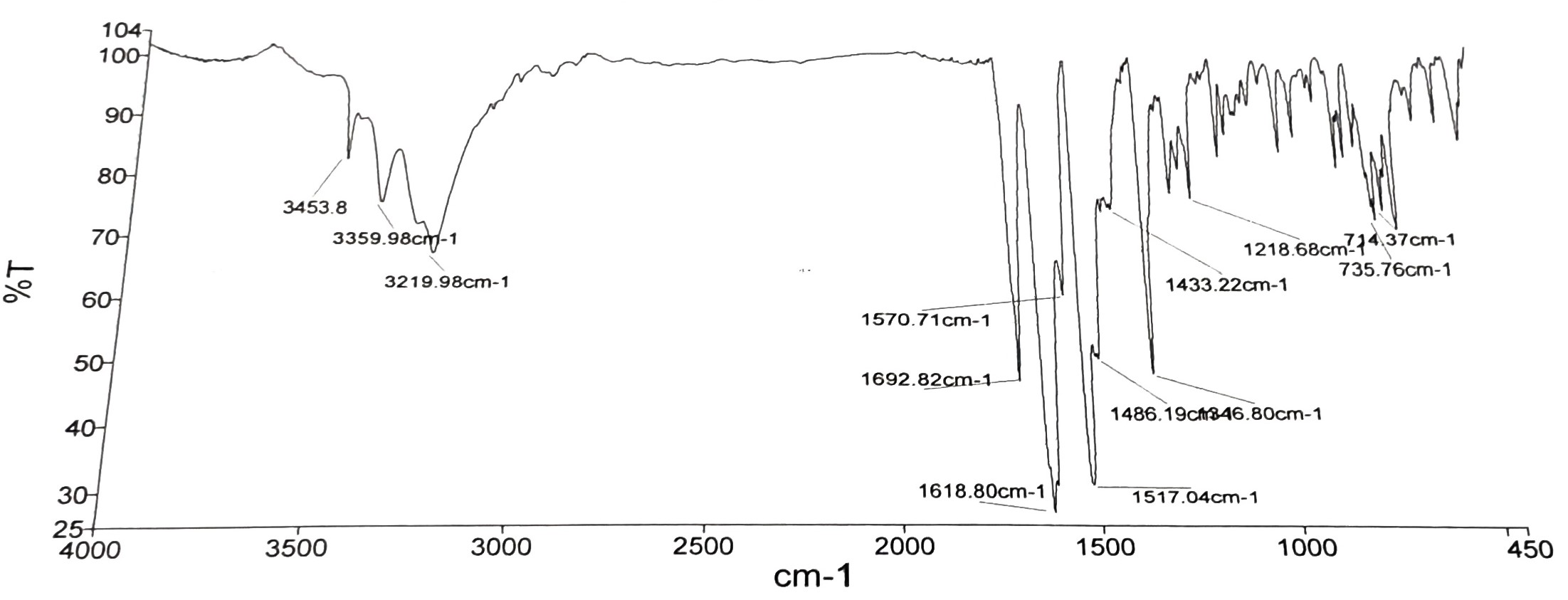


*FT-IR spectrum of 3-(1H-indol-3-yl)-6,8-dimethyl-4-(3-nitrophenyl)-1,4,8,9-tetrahydro-5H-pyrazolo[4',3':5,6]pyrido[2,3-d]pyrimidine-5,7(6H)-dione (c6).*


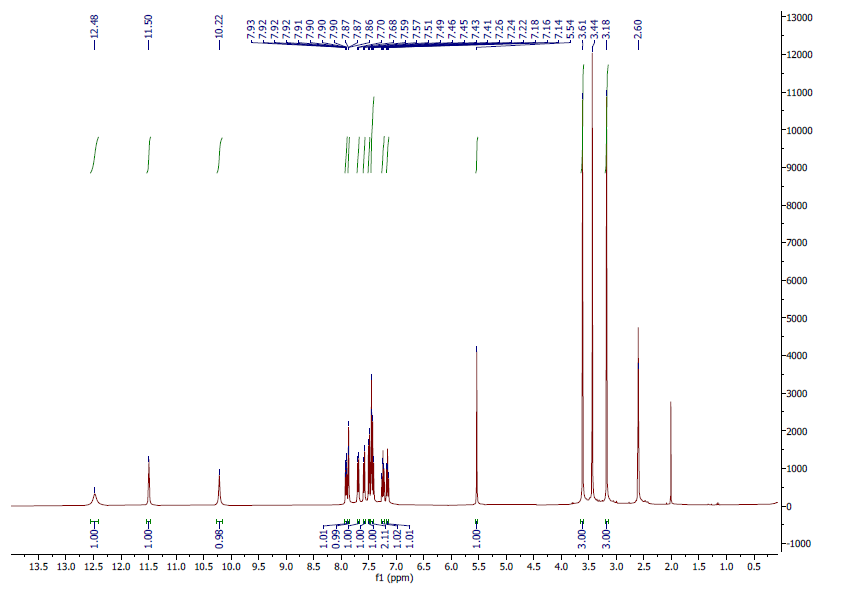


*^1^H-NMR spectrum of 3-(1H-indol-3-yl)-6,8-dimethyl-4-(3-nitrophenyl)-1,4,8,9-tetrahydro-5H-pyrazolo[4',3':5,6]pyrido[2,3-d]pyrimidine-5,7(6H)-dione (c6).*


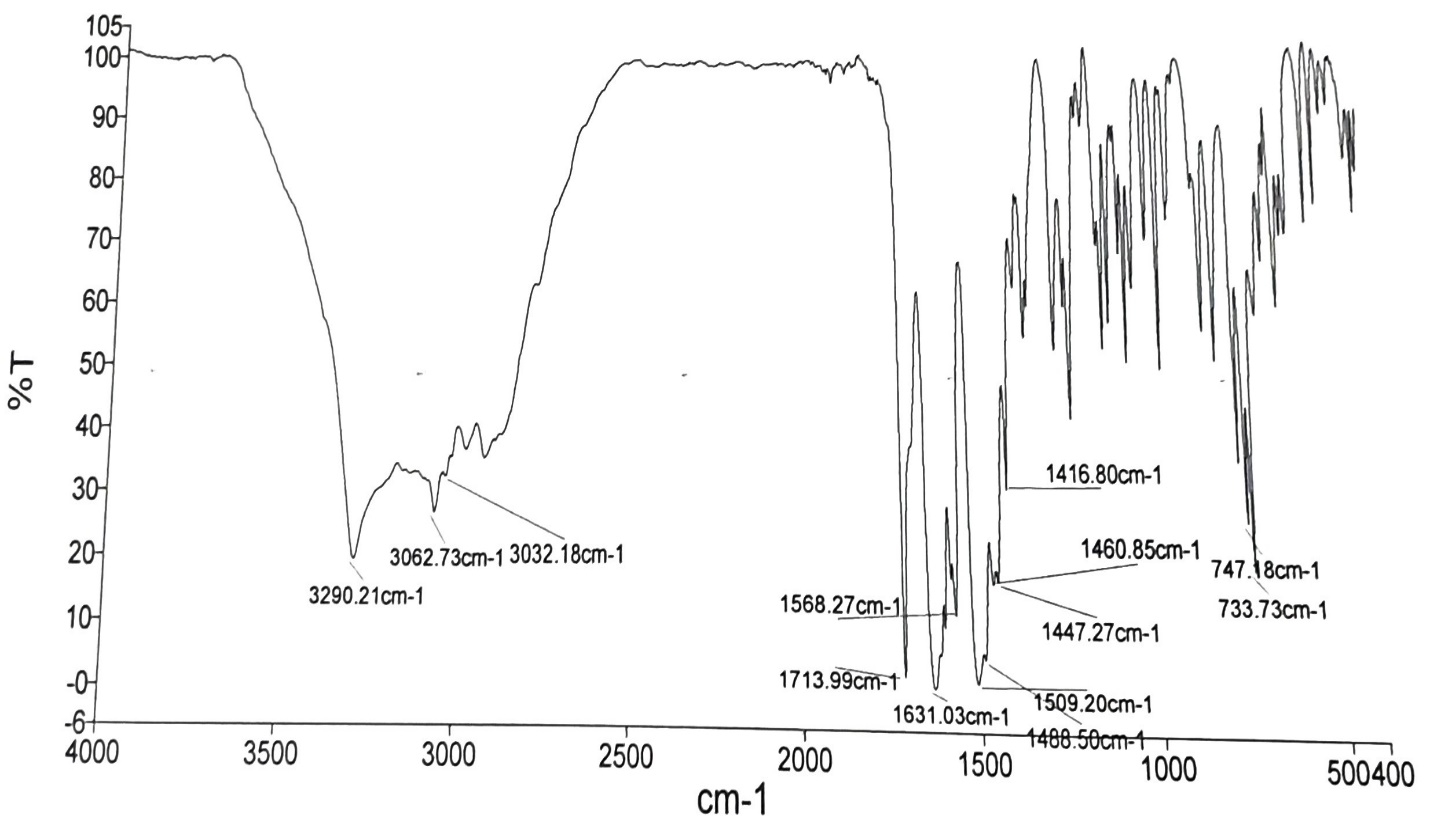


*FT-IR spectrum of 3-(1H-indol-3-yl)-6,8-dimethyl-4-(pyridin-3-yl)-1,4,8,9-tetrahydro-5H-pyrazolo[4',3':5,6]pyrido[2,3-d]pyrimidine-5,7(6H)-dione (c7).*


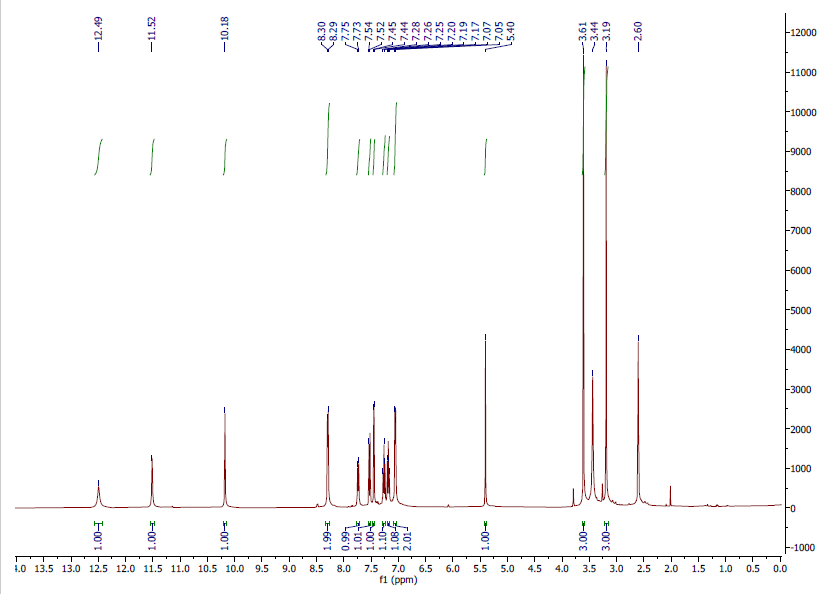


*^1^H-NMR spectrum of 3-(1H-indol-3-yl)-6,8-dimethyl-4-(pyridin-3-yl)-1,4,8,9-tetrahydro-5H-pyrazolo[4',3':5,6]pyrido[2,3-d]pyrimidine-5,7(6H)-dione (c7).*


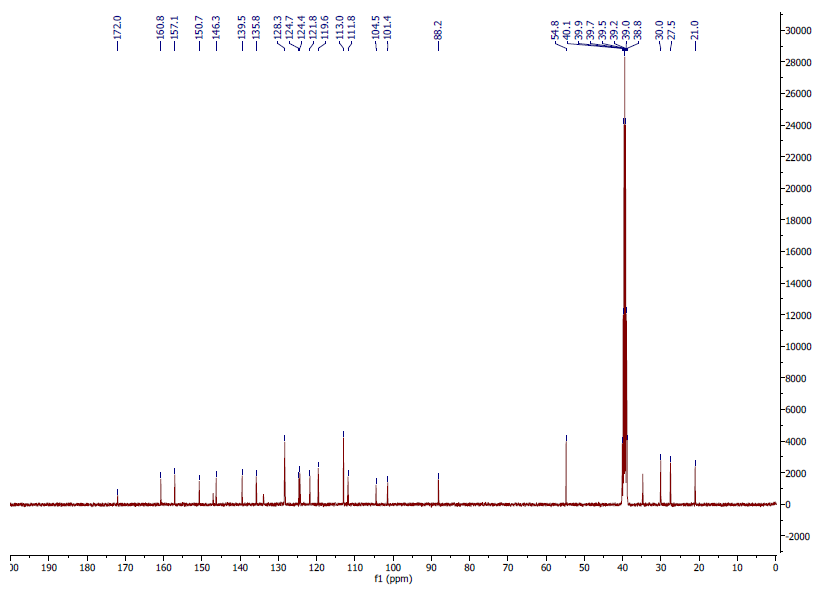


*^13^C-NMR spectrum of3-(1H-indol-3-yl)-6,8-dimethyl-4-(pyridin-3-yl)-1,4,8,9-tetrahydro-5H-pyrazolo[4',3':5,6]pyrido[2,3-d]pyrimidine-5,7(6H)-dione (c7).*
